# Isothiourea-Catalyzed Enantioselective α-Alkylation of Esters via 1,6-Conjugate Addition to *para*-Quinone Methides

**DOI:** 10.3390/molecules26216333

**Published:** 2021-10-20

**Authors:** Jude N. Arokianathar, Will C. Hartley, Calum McLaughlin, Mark D. Greenhalgh, Darren Stead, Sean Ng, Alexandra M. Z. Slawin, Andrew D. Smith

**Affiliations:** 1 EaStCHEM, School of Chemistry, University of St Andrews, North Haugh, St Andrews KY16 9ST, UK; jude1991@hotmail.co.uk (J.N.A.); whartley@iciq.es (W.C.H.); mclaughl@uni-muenster.de (C.M.); Mark.Greenhalgh@warwick.ac.uk (M.D.G.); amzs@st-andrews.ac.uk (A.M.Z.S.); 2 Department of Chemistry, University of Warwick, Coventry CV4 7AL, UK; 3 AstraZeneca, Oncology R&D, Research & Early Development, Darwin Building, 310, Cambridge Science Park, Milton Road, Cambridge CB4 0WG, UK; Darren.Stead@astrazeneca.com; 4 Syngenta, Jealott’s Hill International Research Centre, Bracknell RG42 6EY, UK; sean.ng@syngenta.com

**Keywords:** isothiourea, ammonium enolate, aryloxide, quinone methide, ester functionalization, 1,6-conjugate addition

## Abstract

The isothiourea-catalyzed enantioselective 1,6-conjugate addition of *para*-nitrophenyl esters to 2,6-disubstituted *para*-quinone methides is reported. *para*-Nitrophenoxide, generated in situ from initial *N*-acylation of the isothiourea by the *para*-nitrophenyl ester, is proposed to facilitate catalyst turnover in this transformation. A range of *para*-nitrophenyl ester products can be isolated, or derivatized in situ by addition of benzylamine to give amides at up to 99% yield. Although low diastereocontrol is observed, the diastereoisomeric ester products are separable and formed with high enantiocontrol (up to 94:6 er).

## 1. Introduction

Quinone methides (QMs) are electrophilic compounds composed of a cyclohexadiene core bearing a carbonyl either *ortho* or *para* to an exocyclic alkylidene unit [1,2]. Due to their electrophilicity [3,4,5,6], QMs have been used in a variety of biological and medicinal processes [1,2,7], are present within natural products and pharmaceuticals [1,2,8,9,10,11], and have been applied as electrophiles in a variety of synthetic reactions [1,2,12,13,14,15,16,17,18]. While *ortho*-QMs have been used extensively in enantioselective catalysis [19], particularly as components in formal [4+2] cycloaddition reactions, the use of *para*-QMs has only recently received increased attention [19,20,21,22,23]. The majority of enantioselective organocatalytic methods that involve *para*-QMs have utilized Brønsted acid [24,25,26,27,28,29,30,31,32,33] or hydrogen bonding catalysts [34,35,36,37,38], with only a relatively small number of examples using Lewis base catalysis [39,40,41,42,43,44,45,46,47,48,49,50,51].

**Scheme 1:**
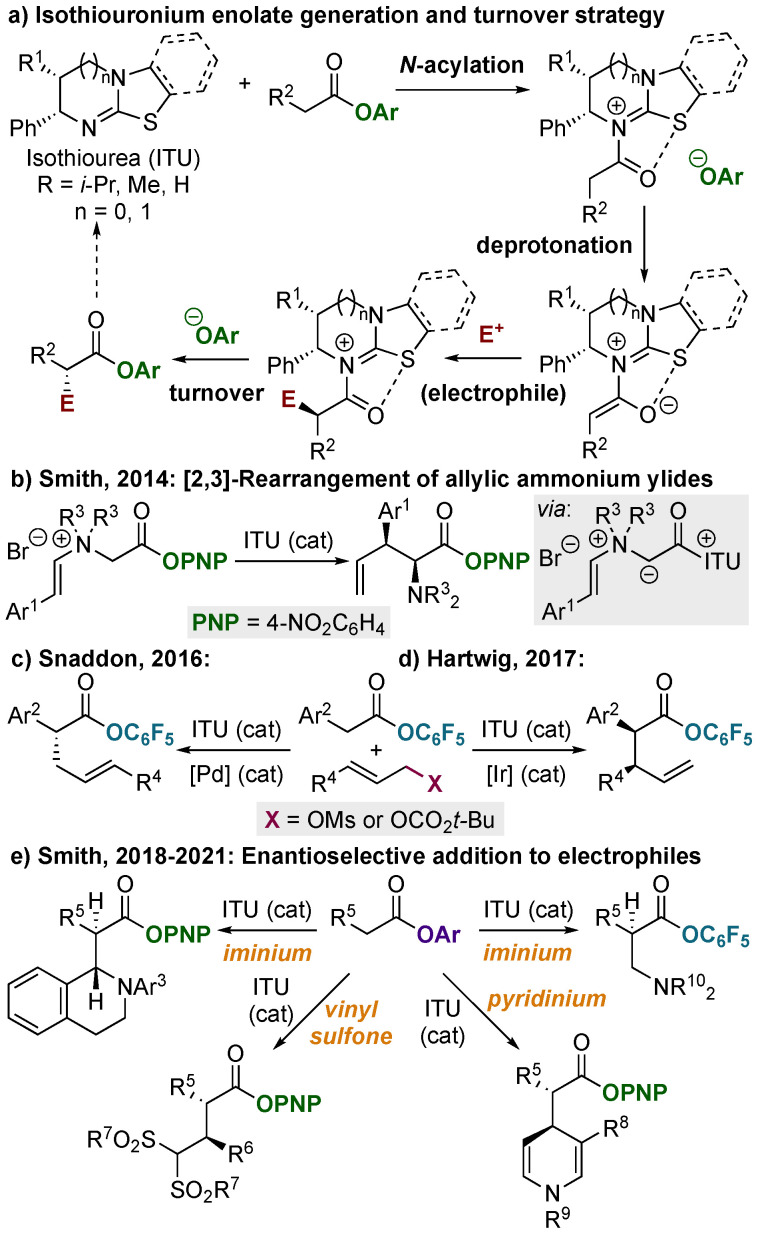
Isothiourea-catalyzed enantioselective processes using aryloxide-facilitated catalyst turnover.

C(1)-Ammonium enolate intermediates [52,53,54,55], generated by the reaction of a tertiary amine Lewis base catalyst with a ketene, anhydride or acyl imidazole [56], have found widespread application for the synthesis of heterocyclic scaffolds in high yield and with excellent enantiocontrol. Traditionally these approaches have been limited by the requirement for the electrophilic reaction partner to contain a latent nucleophilic site to facilitate catalyst turnover. This conceptual obstacle has resulted in catalysis via C(1)-ammonium enolates being mostly applied for formal cycloaddition reactions. More recently, aryl esters have emerged as alternative C(1)-ammonium enolate precursors [55,57,58]. Significantly, following acylation of the tertiary amine catalyst by the aryl ester, a nucleophilic aryloxide is liberated, which may be exploited again in the catalytic cycle to facilitate ammonium enolate formation and catalyst turnover (Scheme 1a) [55,59]. This strategy offers a potentially general solution to allow the expansion of electrophile scope within catalytic processes using C(1)-ammonium enolate intermediates. In 2014, we applied this concept for the isothiourea-catalyzed[2,3]-rearrangement of allylic ammonium ylides (Scheme 1b) [60,61,62,63,64]. More recently, this approach has been used by Snaddon (Scheme 1c) [65,66,67,68,69,70,71,72], Hartwig (Scheme 1d) [73] and Gong [74,75] for co-operative isothiourea/transition metal-catalyzed α-functionalization of pentafluorophenyl esters. In both cases, an isothiourea-derived C(1)-ammonium enolate is intercepted by an electrophilic transition metal complex to affect an allylation or benzylation reaction. We have expanded the scope of electrophiles applicable within this catalyst turnover strategy to include iminium ions generated under either photoredox conditions or Brønsted acid catalysis, as well as bis-sulfone Michael acceptors, and pyridinium salts (Scheme 1e) [76,77,78,79]. Recently, Waser also reported an elegant example of this turnover strategy for the enantioselective α-chlorination of pentafluorophenyl esters [80], while Zheng and co-workers reported a related approach using diphenyl methanol as an external turnover reagent for the fluorination of carboxylic acids [81,82]. A significant challenge within this area is the identification of electrophilic reaction partners that react with the catalytically-generated C(1)-ammonium enolate, but are compatible with the nucleophilic tertiary amine catalyst and aryloxide, which is essential for catalyst turnover. Building upon this conceptual platform, it was envisaged that *para*-QMs may be suitable electrophiles to apply in formal 1,6-conjugate additions.

## 2. Results

### 2.1. Reaction Optimization

Initial studies focused on the isothiourea-catalyzed 1,6-conjugate addition of *para*-nitrophenyl (PNP) ester **1** to 2,6-di-*tert*-butyl *para*-QM **5** (Table 1). Benzylamine was added at the end of the reaction to convert the PNP ester product to the corresponding amide. Based on our previous experience, the amide was expected to be more stable to chromatographic purification. Using tetramisole HCl **6** (20 mol%) as the catalyst, *i*-Pr_2_NEt as the base and MeCN as the solvent gave a 55:45 ratio of diastereoisomeric amide products **7** and **8** in high yield (82%) and with excellent enantioselectivity (**7**: 97:3 er; **8**: 94:6 er) (Entry 1). Chromatographic separation of the diastereoisomers was not possible, however, the enantioenrichment of both **7** and **8** could be reliably determined by chiral stationary phase (CSP)-HPLC analysis of the mixture. A control reaction in the absence of the catalyst showed no conversion (Entry 2). The use of six alternative solvents was investigated (PhMe, CH_2_Cl_2_, CHCl_3_, THF, 1,4-dioxane and DMF), (see the Supporting Information for details) however, only the use of DMF provided any conversion to the product, indicating that solvent polarity may be significant for the success of this transformation. A control reaction in DMF in the absence of the catalyst, however, also led to comparable conversion to the product, consistent with the operation of a competitive Brønsted base-promoted reaction (see the Supporting Information for details). Taking MeCN as the optimal solvent, the use of eight different organic and inorganic bases was investigated (see the Supporting Information for details). Of those tested, Et_3_N provided an improved yield of 98%, whilst maintaining comparable diastereo- and enantioselectivity (Entry 3). The use of alternative aryl esters was next probed, with pentafluorophenyl ester **2** and bis(trifluoromethyl)phenyl ester **3** giving amide products **7** and **8** in high yield, but with lower enantioselectivity than when using PNP ester **1** (Entries 4 and 5). The use of 2,4,6-trichlorophenyl ester **4** resulted in only 31% yield (Entry 6), which is consistent with previous studies in this field [65,72,76,79], and most likely reflects the increased steric hindrance of the aryloxide attenuating its nucleophilicity. Finally, using PNP ester **1**, the catalyst loading could be reduced to 5 mol% with only a small drop in stereoselectivity (Entry 7), while heating the reaction to 40 °C provided a slight improvement in yield (Entry 8). The reaction could also be performed in the absence of a base (Entry 9), however, slightly lower yield was obtained and therefore, during investigation of the substrate scope, Et_3_N was routinely used as an auxiliary base.

### 2.2. Reaction Scope and Limitations

**Scheme 2:**
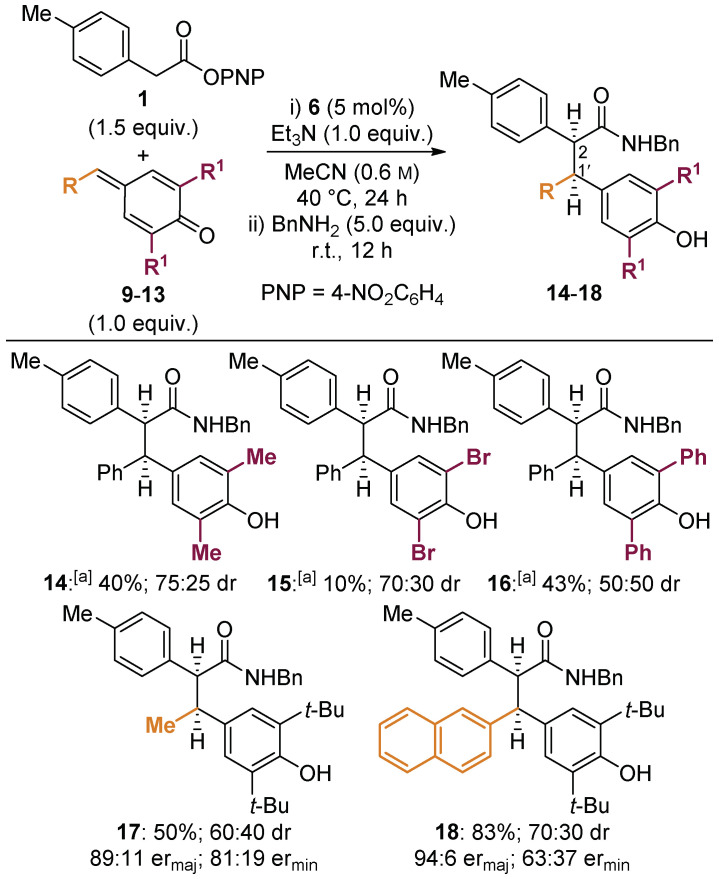
Scope: Variation of *para*-quinone methide—0.25 mmol scale; only the structure of the major diastereoisomer is shown; isolated yield is a mixture of diastereoisomers; dr determined by ^1^H NMR spectroscopic analysis of the crude reaction product mixture; er determined by CSP-HPLC analysis. ^[a]^ Product not isolable: conversion based on ^1^H NMR analysis of the crude reaction product mixture; ers could not be determined.

Due to the low diastereoselectivity observed using 2,6-di-*tert*-butyl *para*-QM **5**, the alternative use of 2,6-disubstituted *para*-QMs were investigated (Scheme 2). 2,6-Dimethyl, dibromo and diphenyl *para*-QMs **9**–**11** bearing a phenyl substituent at the exocyclic olefin were applied under optimized conditions. In all cases significantly lower conversion was observed (≤43%), and the amide products **14**–**16** were difficult to isolate. Based on analysis of the crude reaction mixture by ^1^H NMR spectroscopy, the 2,6-dimethyl and dibromo-substituted analogues **14** and **15** were obtained with marginally improved diastereoselectivity (~70:30 dr), indicating that alternative substituents in these positions could prove beneficial if the products were isolable. Next, variation of the exocyclic substituent was probed. Incorporation of a methyl group at this position provided no improvement in dr, but the amide product **17** was isolated in a 50% yield and with moderate enantioenrichment for both diastereoisomers. Finally, incorporation of a 2-naphthyl substituent at this position was well tolerated, with amide **18** obtained in an 83% yield, 70:30 dr and excellent enantiocontrol (94:6 er) for the major diastereoisomer.

**Scheme 3:**
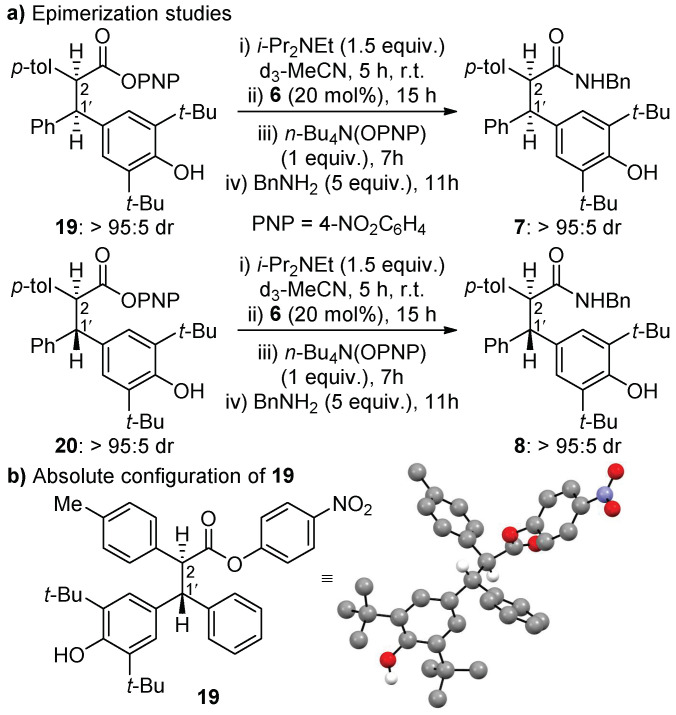
Control studies and confirmation of absolute configuration of products. The majority of hydrogen atoms are omitted for clarity within X-ray crystal structure representation of **19**.

Although structural variation of the *para*-QM provided marginal improvements in dr, the use of 2,6-di-*tert*-butyl *para*-QM **5** was considered most convenient for further investigations due to its stability and ease of synthesis, and the higher yields of product obtained from catalysis. To investigate if the dr obtained in these reactions was a manifestation of a kinetic or thermodynamic preference, isolation of diastereoisomeric PNP esters **19** and **20** and resubjection to catalysis conditions was attempted. Although the diastereoisomeric amides **7** and **8** had proved difficult to separate, the corresponding PNP esters **19** and **20** were chromatographically separable and displayed high stability. Epimerization studies were conducted using each diastereoisomer through sequential treatment with *i*-Pr_2_NEt, (*S*)-TM HCl **6**, *para*-nitrophenoxide and benzylamine (Scheme 3). These experiments were followed by in situ ^1^H NMR spectroscopic analysis and revealed no epimerization in either case. This indicates the dr obtained in the catalytic reaction most likely reflects the inherent diastereoselectivity of the transformation. Following separation of the diastereoisomers, the absolute configuration of the major diastereoisomer could also be confirmed as (2*S*,1′*R*) by single crystal X-ray crystallographic analysis [83,84]. Based on literature precedent, the (*S*)-configuration at C(2) was expected to be generated under catalyst-control, and therefore the absolute configuration of the minor diastereoisomer was predicted to be (2*S*,1′*S*).

**Scheme 4:**
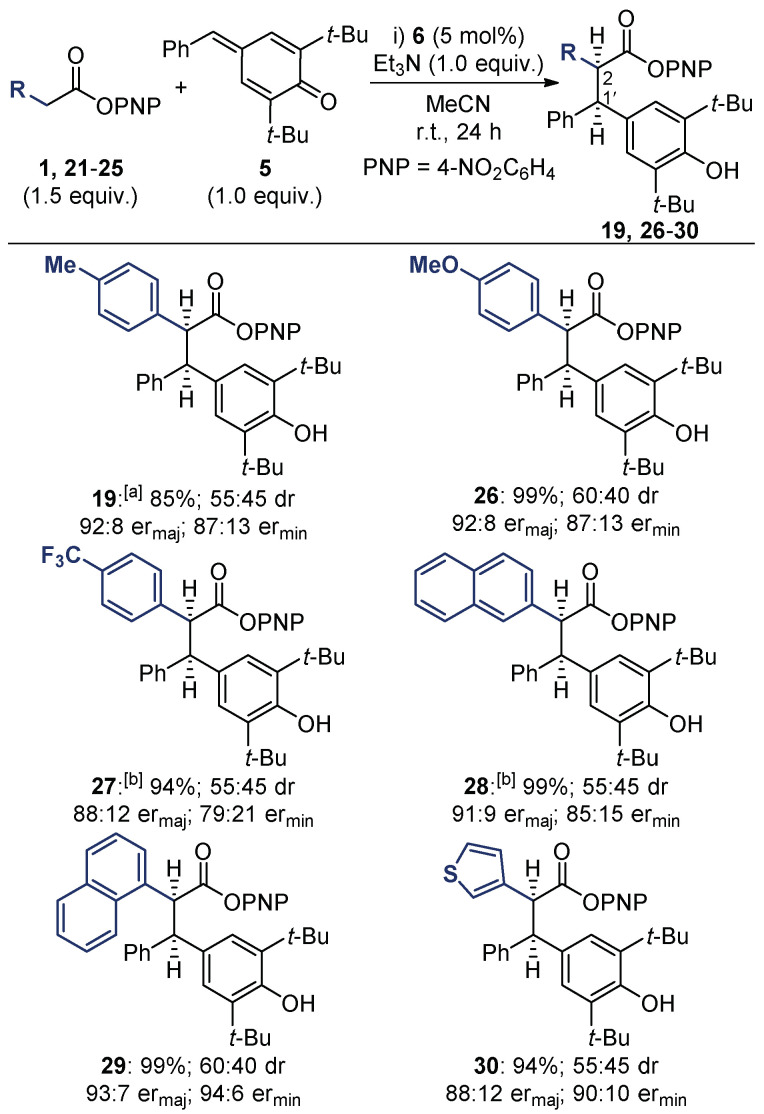
Scope: Variation of *para*-nitrophenyl ester—0.25 mmol scale; only the structure of the major diastereoisomer is shown; isolated yields are given for the combination of diastereoisomers; dr determined by ^1^H NMR spectroscopic analysis of the crude reaction product mixture; er determined by CSP-HPLC analysis. ^[a]^ 1.25 mmol scale. ^[b]^ Conducted at r.t., in the absence of Et_3_N, and using free base of **6**.

Having established the stability of the PNP ester products and demonstrated the potential to separate the diastereoisomers by column chromatography, the scope of the catalytic transformation was investigated through variation of the PNP ester substrate. In each case, the PNP ester product diastereoisomers were at least partially separable, enabling unambiguous characterization. To test the applicability of the procedure, *p*-tolyl-substituted PNP ester product **19** was prepared on a larger scale (1.25 mmol) (Scheme 4). A combined 85% yield of both diastereoisomers was obtained, with comparable stereoselectivity to that observed when the reaction was conducted on an analytical scale (Table 1, Entry 7). The generality of the procedure was further probed using five electronically- and sterically-differentiated PNP esters. Introduction of an electron-donating 4-methoxy substituent was well tolerated, with PNP ester **26** obtained in quantitative yield, 60:40 dr and with high enantioselectivity for both diastereoisomers. Under the optimized conditions, the introduction of an electron-withdrawing 4-trifluoromethyl group resulted in low enantioselectivity (**27**: 63:37 er), which was attributed to a competitive Brønsted base-promoted background reaction. Consistent with this hypothesis, repeating the reaction in the absence of Et_3_N, and using the free base of the isothiourea catalyst **6**, provided PNP ester product **27** in 94% yield and significantly improved enantioselectivity (88:12 er_maj_; 79:21 er_min_). A similar effect was observed when using a 2-naphthyl-substituted PNP ester, with optimal enantioselectivity obtained in the absence of an auxiliary base (**28**: 91:9 er_maj_; 85:15 er_min_). Introduction of a sterically-imposing 1-naphthyl or a heteroaromatic thienyl substituent was also tolerated, with **29** and **30** obtained in excellent yield and with high enantioselectivity.

### 2.3. Proposed Mechanism

**Scheme 5:**
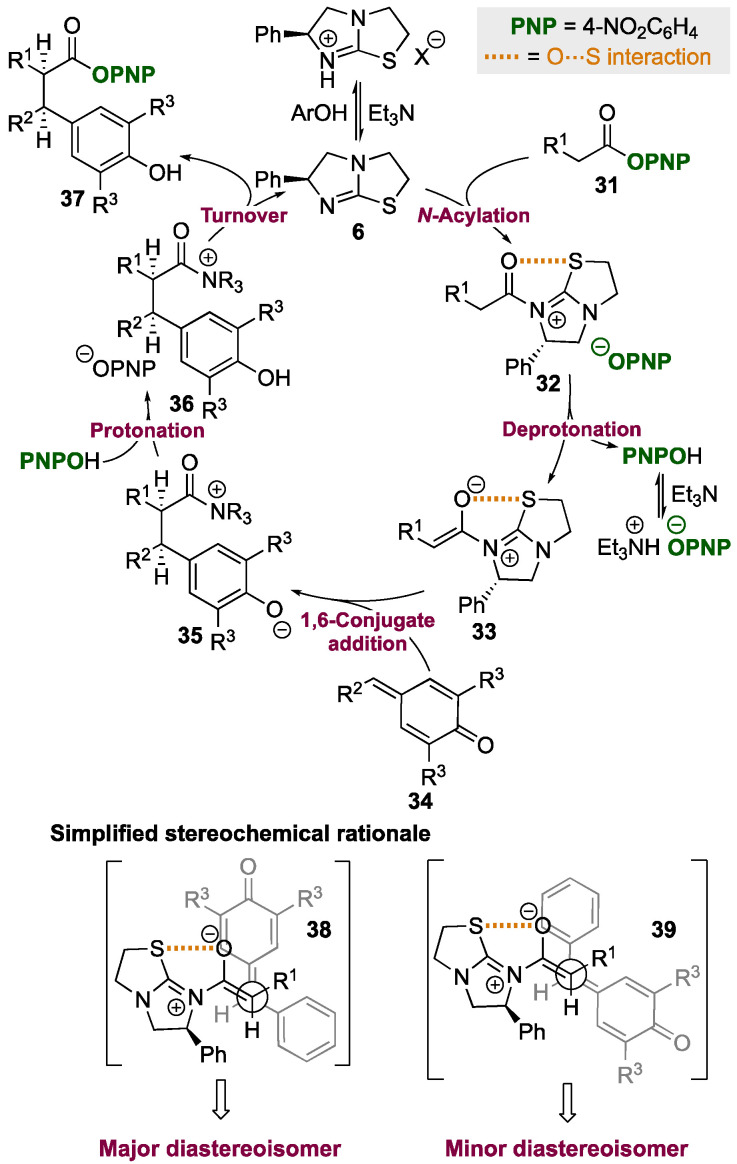
Proposed mechanism (only the pathway for the formation of the major diastereoisomer is shown) and transition state assemblies. ArOH corresponds to either PNPOH or the reaction product.

The mechanism of this transformation is proposed to begin with *N*-acylation of the free base isothiourea catalyst **6** by PNP ester **31** to generate the corresponding acyl ammonium *para*-nitrophenoxide ion pair **32** (Scheme 5). Subsequent deprotonation leads to (*Z*)-ammonium enolate **33**. Based on previous mechanistic studies [78,85], and the catalytic activity observed in the absence of an auxiliary base (Table 1, Entry 9), deprotonation is likely to be affected by the *para*-nitrophenoxide counterion. 1,6-Conjugate addition of ammonium enolate **33** to *para*-QM electrophile **34**, followed by protonation, gives acyl ammonium intermediate **35**. Finally, regeneration of catalyst **6**, and concurrent release of product **37**, is proposed to be facilitated by *para*-nitrophenoxide [59,60,61,62,63,64,65,66,67,68,69,70,71,72,73,74,75,76,77,78,79,86,87]. Although not essential for reactivity, the addition of Et_3_N as an auxiliary base may be beneficial as a proton shuttle, and to maintain the isothiourea catalyst in its non-protonated form **6** [77,86]. The enantioselectivity of the transformation indicates the C–C bond forming event takes place on the *Si*-face of the ammonium enolate. This selectivity can be rationalized through preferential formation of the (*Z*)-ammonium enolate [76,77,78,79,85], which is conformationally-restricted by an intramolecular 1,5-O⋯S interaction [61,88,89,90,91,92,93,94,95,96,97,98,99,100,101,102,103,104] and results in the phenyl stereodirecting group of the catalyst blocking the enolate *Re*-face. The observed poor diastereoselectivity can be tentatively rationalized by a simple stereochemical model that assumes a favored, open pre-transition state assembly where steric interactions are minimized about the forming C–C bond. Minimal differentiation between the aryl- and quinone substituents of the *para*-QM quinone leads to the two transition state assemblies **38** and **39** that give the major and minor diastereoisomers, respectively.

## 3. Materials and Methods

### 3.1. General Procedure for the Enantioselective 1,6-Addition

In a flame-dried vial, the requisite *para*-quinone methide (1.0 equiv.), aryl ester (1.5 equiv.), (*S*)-TM HCl (5 mol%), Et_3_N (1.0 equiv.) and anhydrous MeCN (0.6 M) was added and stirred at r.t. for 24 h. The reaction was then quenched with benzylamine (5.0 equiv.) and stirred at r.t. for a further 12 h before being concentrated in vacuo. The residue was diluted with EtOAc (20 mL) and washed successively with 10% citric acid (20 mL × 1), aqueous NaOH (20 mL × 3) and brine (20 mL × 1). The organic layer was extracted, dried over MgSO_4_ and the filtrate was concentrated in vacuo. The crude material was purified by flash silica column chromatography to give the desired product.

### 3.2. Representative Synthesis and Characterization of Compounds ***7*** and ***8*** (Entry 8)

Following the general procedure above, 4-benzylidene-2,6-di-*tert*-butylcyclohexa-2,5-dien-1-one **5** (74 mg, 0.25 mmol), 4’-nitrophenyl 2-(*p*-tolyl)acetate **1** (102 mg, 0.375 mmol), (*S*)-TM HCl **6** (3 mg, 5 mol%) and Et_3_N (35 μL, 0.25 mmol) were dissolved in anhydrous MeCN (0.42 mL). The reaction mixture was stirred at 40 °C for 24 h before being quenched with benzylamine (137 μL, 1.25 mmol) at r.t. to give a crude mixture containing the title compound in 60:40 dr. The mixture was purified by flash silica column chromatography (petroleum ether/EtOAc, 85:15) to afford diastereoisomers **7** and **8** (60:40 dr) (132 mg, 99%) as a pale yellow solid.

mp 126–128 °C; ${\left[\alpha \right]}_{D}^{20}$ +10.0 (*c* 1.0, CHCl_3_); IR ν_max_ (film)/cm^−1^ 3638 (O−H) 3304 (N−H), 2955 (C−H), 1645 (C=O); HRMS (ESI^+^) C_37_H_43_NO_2_ ([M + H]^+^), found 534.3359, requires 534.3367 (−1.4 ppm).

*Data for major diastereoisomer* (7): Chiral HPLC analysis, Chiralpak AD-H (10% *i*-PrOH/hexane, flow rate 1.5 mLmin^−1^, 211 nm, 40 °C), t_R_ 8.5 min and 29.3 min, 92:8 er; ^1^H NMR (500 MHz, CDCl_3_) δ_H_: 1.42 (18H, s, (C(3″″)C(C*H*_3_)_3_, C(5″″)C(C*H*_3_)_3_), 2.24 (3H, s, C(4″)C*H*_3_), 3.90–4.05 (2H, m, C(2)*H*, C*H_A_*Ph), 4.44 (1H, dd, *J* 15.0, 6.8, C*H_B_*Ph), 4.82 (1H, d, *J* 11.7, C(1’)*H*), 5.14 (1H, s, O*H*), 5.55 (1H, t, *J* 5.6, N*H*), 6.71–6.76 (2H, m, *Ar*), 6.96–7.05 (2H, m, *Ar*), 7.09–7.15 (2H, m, *Ar*), 7.15–7.22 (4H, m, *Ar*), 7.23–7.30 (4H, m, C(4‴)*H*, C(2″″)*H*, C(6″″)*H, Ar*), 7.34 (1H, t, *J* 7.6, *Ar*), 7.43–7.51 (1H, m, *Ar*); ^13^C{^1^H} NMR (126 MHz, CDCl_3_) δ_C_: 21.0 (C(4″)*C*H_3_), 30.4 (C(3″″)C(*C*H_3_)_3_, C(5″″)C(*C*H_3_)_3_), 34.4 (C(3″″)*C*(CH_3_)_3_, C(5″″)*C*(CH_3_)_3_), 43.6 (*C*H_2_Ph), 54.1 (*C*(1’)), 59.5 (*C*(2)), 124.7 (*C*(2″″), *C*(6″″)), 125.8 (*C*(4‴)), 128.2 (*Ar*), 127.2 (*Ar*), 127.3 (*Ar*), 128.0 (*Ar*), 128.4 (*Ar*), 128.5 (*Ar*), 128.6 (*Ar*), 129.0 (*Ar*), 133.8 (*C*(1‴)), 135.2 (*C*(1″)), 135.6 (*C*(3″″), *C*(5″″)), 136.4 (*C*(4″)), 137.9 (*i*-*Ph*), 142.4 (*C*(1″″)), 152.4 (*C*(4″″)), 172.1 (*C*(1)).

*Selected data for minor diastereoisomer* (8): Chiral HPLC analysis, Chiralpak AD-H (10% *i*-PrOH/hexane, flow rate 1.5 mLmin^−1^, 211 nm, 40 °C), t_R_ 3.8 min and 18.6 min, 85:15 er; ^1^H NMR (500 MHz, CDCl_3_) δ_H_: 1.27 (18H, s, (C(3″″)C(C*H*_3_)_3_, C(5″″)C(C*H*_3_)_3_), 2.27 (3H, s, C(4″)C*H*_3_), 3.90–4.05 (2H, m, C(2)*H*, C*H_A_*Ph), 4.50 (1H, dd, *J* 15.0, 6.8, C*H_B_*Ph), 4.69 (1H, d, *J* 11.7, C(1’)*H*), 4.89 (1H, s, O*H*), 5.69 (1H, t, *J* 5.6, N*H*), 6.81–6.84 (2H, m, *Ar*); ^13^C{^1^H} NMR (126 MHz, CDCl_3_) δ_C_: 21.0 (C(4″)*C*H_3_), 30.2 (C(3″″)C(*C*H_3_)_3_, C(5″″)C(*C*H_3_)_3_), 34.2 (C(3″″)*C*(CH_3_)_3_, C(5″″)*C*(CH_3_)_3_), 43.4 (*C*H_2_Ph), 54.7 (*C*(1’)), 59.5 (*C*(2)), 125.3 (*C*(2″″), *C*(6″″)), 126.3 (*C*(4‴)), 127.5 (*Ar*), 128.2 (*Ar*), 128.4 (*Ar*), 128.8 (*Ar*), 132.1 (*C*(1‴)), 134.9 (*C*(3″″), *C*(5″″)), 135.5 (*C*(1″)), 136.4 (*C*(4″)), 138.2 (*i*-*Ph*), 143.6 (*C*(1″″), 151.7 (*C*(4″″)), 172.0 (*C*(1)).

## 4. Conclusions

An isothiourea-catalyzed enantioselective 1,6-conjugate addition of *para*-nitrophenyl (PNP) esters to *para*-quinone methides (QMs) has been developed. Variation of the arylacetic ester and *para*-QM substrates has provided a range of functionalized products in generally excellent yields and high enantiocontrol (up to 94:6 er). An inherent limitation of the method is that the products were routinely obtained in ~60:40 dr. This diastereoselectivity was shown to arise from kinetic control, but was relatively insensitive to changes in reaction conditions and structural variation of the substrates. Although the dr could not be improved, the diastereoisomeric PNP ester products could be separated by column chromatography. The success of this catalytic methodology is proposed to rely upon the *para*-nitrophenoxide, expelled during *N*-acylation of the catalyst, to facilitate catalyst turnover and release the product. Current work in our laboratory is focused on further applications of using in situ-generated aryloxides to promote catalyst turnover in Lewis base catalysis.

## Figures and Tables

**Table 1:**
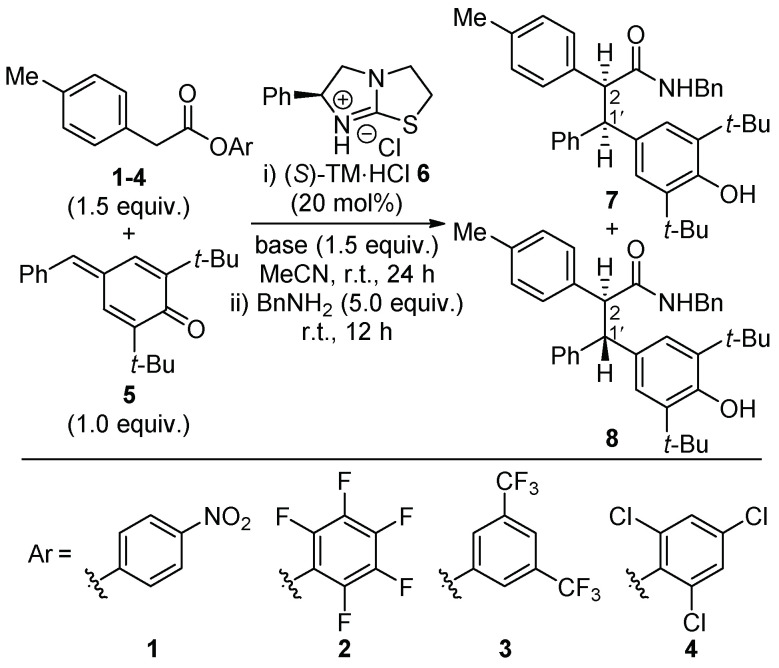
Reaction optimization.

Entry	6 (mol%)	Aryl Ester	Base	Yield (%)	dr (7:8)	er (7)	er (8)
1	20	1	*i*-Pr_2_NEt	82	55:45	97:3	94:6
2	0	1	*i*-Pr_2_NEt	0	-	-	-
3	20	1	Et_3_N	98	60:40	97:3	94:6
4	20	2	Et_3_N	99	65:35	81:19	94:6
5	20	3	Et_3_N	88	60:40	94:6	91:9
6	20	4	Et_3_N	31	55:45	84:16	71:29
7	5	1	Et_3_N	95	55:45	93:7	88:12
8 ^[a]^	5	1	Et_3_N	99	60:40	92:8	85:15
9 ^[a]^	5	1	none	90	60:40	93:7	89:11

All reactions were carried out on a 0.25 mmol scale; isolated yields are a mixture of diastereoisomers **7** and **8**; dr was determined by ^1^H NMR spectroscopic analysis of the crude reaction mixture; er was determined by CSP-HPLC analysis: **7** (2*S*,1′*R*:2*R*,1′*S*) and **8** (2*S*,1′*S*:2*R*,1′*R*). ^[a]^ Reaction performed at 40 °C.

## Data Availability

The research data underpinning this publication can be found at DOI: 10.17630/f6cf6c80-483d-4f16-bd79-80e1537513b2.

## Supplementary Materials

Full experimental procedures, characterization data, NMR spectra and HPLC chromatograms for all new compounds, as well as crystallographic data for product **19** (CCDC 1992504) are available online.

## Sample Availability

Samples of the compounds are available from the authors on request.
