# Supplementary material for: Isothiourea-Catalyzed Enantioselective α-Alkylation of Esters via 1,6-Conjugate Addition to para-Quinone Methides
Source: Molecules. 2021 Oct 20;26(21):6333. doi: 10.3390/molecules26216333 (PMC8588318; doi:10.3390/molecules26216333)
Supplement: Supplementary file 1 [file molecules-26-06333-s001.zip › molecules-1383210-supplementary.pdf]

Table of Contents

General Information S2

General Procedures S4

Synthesis of para-Quinone Methides S7

Synthesis of Aryl Esters S14

Amide Products Derived from Isothiourea Catalysis S18

PNP Ester Products from Isothiourea Catalysis S22

Additional Reaction Optimization Tables S31

Control Reactions S33

X-Ray Crystal Structure Information for ***19*** S34

References S35

HPLC Traces S36

NMR Spectra S45

General Information

Reactions involving moisture sensitive reagents were carried out in flame-dried glassware under an inert atmosphere (N_2) using standard vacuum line techniques. Anhydrous solvents (CH_2Cl_2 and THF) were obtained after passing through an alumina column (Mbraun SPS-800) and MeCN was stored over activated molecular sieves. All other solvents and commercial reagents were used as received without further purification unless otherwise stated.

Room temperature (rt) refers to 20-25 °C. Temperatures of 0 °C for overnight reactions were obtained using an immersion cooler (HAAKE EK 90). Reaction involving heating were performed using DrySyn blocks and a contact thermocouple.

In vacuo refers to the use of either a Büchi Rotavapor R-200 with a Büchi V-491 heating bath and Büchi V-800 vacuum controller, a Büchi Rotavapor R-210 with a Büchi V-491 heating bath and Büchi V-850 vacuum controller, a Heidolph Laborota 4001 with vacuum controller, an IKA RV10 rotary evaporator with a IKA HB10 heating bath and ILMVAC vacuum controller, or an IKA RV10 rotary evaporator with a IKA HB10 heating bath and Vacuubrand CVC3000 vacuum controller. Rotary evaporator condensers are fitted to Julabo FL601 Recirculating Coolers filled with ethylene glycol and set to –5 °C.

Analytical thin layer chromatography was performed on pre-coated aluminium plates (Kieselgel 60 F254 silica) and visualization was achieved using ultraviolet light (254 nm) and/or staining with aqueous $KMnO_4$ solution followed by heating. Manual column chromatography was performed in glass columns fitted with porosity 3 sintered discs over Kieselgel 60 silica using the solvent system stated.

Melting points were recorded on an Electrothermal 9100 melting point apparatus. Optical rotations were measured on a Perkin Elmer Precisely/Model-341 polarimeter operating at the sodium D line with a 100 mm path cell at 20 °C.

HPLC analysis were obtained on either a Shimadzu HPLC consisting of a DGU-20A5 degassing unit, LC-20AT liquid chromatography pump, SIL-20AHT autosampler, CMB-20A communications bus module, SPD-M20A diode array detector and a CTO-20A column oven or a Shimadzu HPLC consisting of a DGU-20A5R degassing unit, LC-20AD liquid chromatography pump, SIL-20AHT autosampler, SPD-20A UV/Vis detector and a CTO-20A column oven. Separation was achieved using either a DAICEL CHIRALCEL OD-H column or DAICEL CHIRALPAK AD-H, IB, IC and ID columns using the method stated. HPLC traces of enantiomerically enriched compounds were compared with authentic racemic spectra.

Infrared spectra were recorded on a Shimadzu IRAffinity-1 Fourier transform IR spectrophotometer fitted with a Specac Quest ATR accessory (diamond puck). Spectra were recorded of either thin films or solids, with characteristic absorption wavenumbers (ν_{max}) reported in cm^{-1}.

^{1}H, $^{13}C\{^{1}H\}$ and $^{19}F\{^{1}H\}$ NMR spectra were acquired on either a Bruker AV400 with a BBFO probe (^{1}H 400 MHz; $^{13}C\{^{1}H\}$ 101 MHz; $^{19}F\{^{1}H\}$ 376 MHz), a Bruker AVII 400 with a BBFO probe (^{1}H 400 MHz; $^{13}C\{^{1}H\}$ 101 MHz; $^{19}F\{^{1}H\}$ 376 MHz), a Bruker AVIII-HD 500 with a SmartProbe BBFO+ probe (^{1}H 500 MHz, $^{13}C\{^{1}H\}$ 126 MHz) or a Bruker AVIII 500 with a CryoProbe Prodigy BBO probe (^{1}H 500 MHz, $^{13}C\{^{1}H\}$ 126 MHz), in the deuterated solvent stated. All chemical shifts are quoted in parts per million (ppm) relative to the residual solvent peak. All coupling constants, *J*, are quoted in Hz. Multiplicities are indicated as s (singlet), d (doublet), t (triplet), q (quartet), m (multiplet), and multiples thereof. The abbreviation Ar denotes aromatic. NMR peak assignments were confirmed using 2D ^{1}H correlated spectroscopy (COSY), 2D $^{1}H–^{13}C$ heteronuclear multiple-bond correlation spectroscopy (HMBC), and 2D $^{1}H–^{13}C$ heteronuclear single quantum coherence spectroscopy (HSQC) where necessary.

Mass spectrometry (m/z) data were acquired by either electrospray ionization (ESI), nanospray ionization (NSI) or atmospheric solids analysis probe (ASAP) at either the University of St Andrews Mass Spectrometry Facility, at the EPSRC UK National Mass Spectrometry Facility at Swansea University or at AstraZeneca in Cambridge. Waters Xevo Qtof with Acquity BSM/SM/PDA/CM or Themo Orbitrap Mass Spectrometer with Accela pump and Thermo CTC and Surveyor PDA+ were used at AstraZeneca. 5 or 10 μL of the sample were injected into a Waters CSH C18 1.7μm 50 × 2.1 (at 45 ºC) column using 0.1% aqueous formic acid and 0.1% formic acid in methanol gradients.

General Procedures

General Procedure A: Preparation of 2,6-di-*tert*-butyl *para*-quinone methides

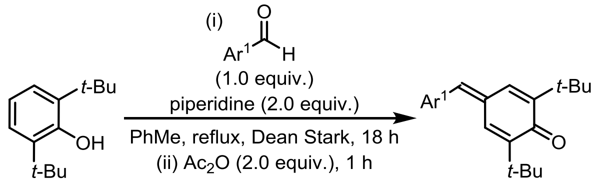

Following a literature procedure,[1] a solution of 2,6-di-*tert*-butylphenol (1.0 equiv.) and the requisite aldehyde (1.0 equiv.) in PhMe was heated to reflux in a Dean-Stark apparatus. Piperidine (2.0 equiv.) was added dropwise within 1 h. The reaction mixture was continued to reflux for 18 h before cooling the reaction mixture to below the boiling point. Acetic anhydride (2.0 equiv.) was added and stirring was continued for 1 h before the reaction mixture was poured on ice-water and extracted with EtOAc (× 3). The combined organic layers were dried over anhydrous Na_2SO_4, and the filtrate concentrated *in vacuo*. The crude material was purified by flash silica column chromatography to give the desired 2,6-di-*tert*-butyl *para*-quinone methide.

General Procedure B: Preparation of *para*-quinone methides from 4-benzylphenols

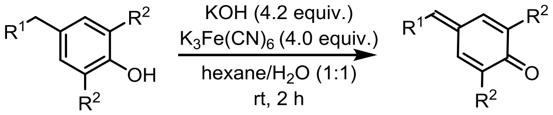

Following a literature procedure,[1] the requisite 4-benzylphenol (1.0 equiv.), KOH (4.2 equiv.) and $K_3Fe(CN)_6$ (4.0 equiv.) in hexane/H_2O (1:1) was vigorously stirred for 2 h at rt before being diluted with EtOAc (× 1) and washed with distilled water (× 4). The combined organic layers were dried over anhydrous Na_2SO_4, and the filtrate concentrated *in vacuo*. The crude material was purified by flash silica column chromatography to give the desired *para*-quinone methide.

General Procedure C: Preparation of 4-hydroxy(phenyl)methylphenols

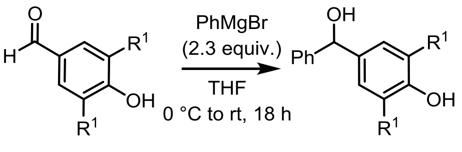

Following a literature procedure,[2] in a flame-dried two-neck round bottom flask under N_2 was added the requisite aldehyde (1.0 equiv.) and anhydrous THF. The solution was cooled

to 0 °C before the dropwise addition of PhMgBr (2.3 equiv., 3.0 M in THF). The reaction mixture was warmed to rt and stirred for 18 h before being quenched with saturated aqueous NH_4Cl at 0 °C. The aqueous layer was extracted with EtOAc (× 3) and the combined organic layers were dried over anhydrous Na_2SO_4, and the filtrate concentrated *in vacuo*. The crude material was purified by flash silica column chromatography or recrystallization to give the desired 4-hydroxy(phenyl)methylphenol.

General Procedure D: Preparation of 2,6-disubstituted *para*-quinone methides *via* sulfones

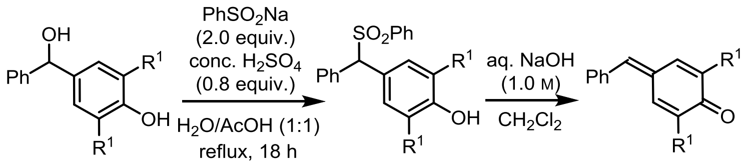

Following a literature procedure,[2] conc. H_2SO_4 (0.8 equiv.) was added to a solution of the requisite 4-hydroxy(phenyl)methylphenol (1.0 equiv.) and $PhSO_2Na$ (2.0 equiv.) in H_2O/AcOH (1:1). The reaction mixture was heated to 100 °C for 18 h before being cooled down to rt. The resulting precipitate upon addition of distilled water was filtered and the solid dried under high vacuum to give the desired sulfone. The sulfone (1.0 equiv.) was dissolved in CH_2Cl_2 (× 1) and shaken with aqueous 1 M NaOH. The organic layer and the aqueous layer washed with CH_2Cl_2 (× 2). The combined organic layers were dried over anhydrous Na_2SO_4, and the filtrate concentrated *in vacuo*. The crude material was purified by flash silica column chromatography to give the desired 2,6-disubstituted *para*-quinone methide.

General Procedure E: Preparation of activated aryl esters

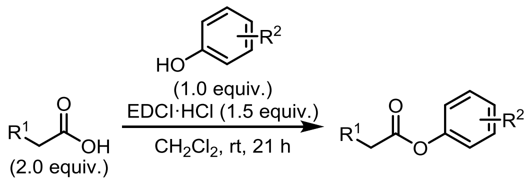

The requisite aryl/alkenyl/alkyl acetic acid (2.0 equiv.) and *N*-(3-dimethylaminopropyl)-*N′*-ethylcarbodiimide hydrochloride, EDCI·HCl (1.5 equiv.) were dissolved in CH_2Cl_2 and allowed to stir at rt for 5 min before the addition of the requisite phenol (1.0 equiv.). The reaction mixture was stirred at rt for 21 h before being diluted with CH_2Cl_2 (× 1) and washed with saturated, aqueous $NaHCO_3$ (× 2). The organic layer was extracted, dried over $MgSO_4$ and the filtrate concentrated *in vacuo*. The crude material was purified by flash silica column chromatography to give the desired activated aryl ester.

General Procedure F: Isothiourea catalysis to give amide products

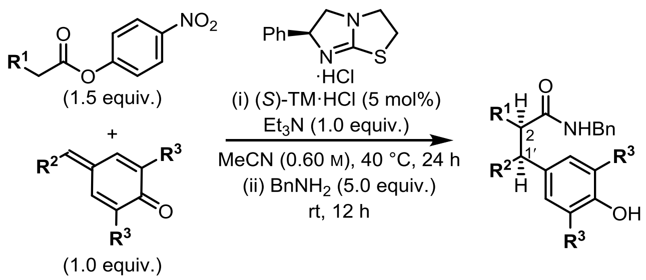

In a flame-dried vial was added the requisite *para*-quinone methide (1.0 equiv.), aryl ester (1.5 equiv.), (*S*)-TM·HCl (5 mol%), Et_3N (1.0 equiv.) and anhydrous MeCN (0.6 M) and was stirred at rt for 24 h. The reaction was then quenched with benzylamine (5.0 equiv.) and stirred at rt for a further 12 h before being concentrated *in vacuo*. The residue was diluted with EtOAc (20 mL) and washed successively with 10% citric acid (20 mL × 1), aqueous NaOH (20 mL × 3) and brine (20 mL × 1). The organic layer was extracted, dried over $MgSO_4$ and the filtrate concentrated *in vacuo*. The crude material was purified by flash silica column chromatography to give the desired product.

General Procedure G: Isothiourea catalysis to give PNP ester products

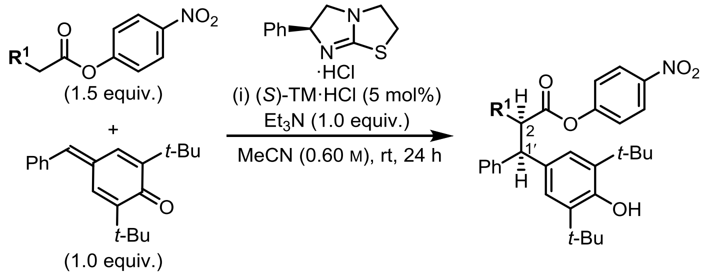

In a flame-dried vial was added the requisite *para*-quinone methide (1.0 equiv.), aryl ester (1.5 equiv.), (*S*)-TM·HCl (5 mol%), Et_3N (1.0 equiv.) and anhydrous MeCN (0.6 M) and was stirred at rt for 24 h. The crude material was purified and the diastereoisomers separated by flash silica column chromatography.

Synthesis of para-Quinone Methides

4-Benzylidene-2,6-di-*tert*-butylcyclohexa-2,5-dien-1-one (5)

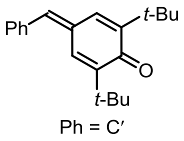

The title compound was prepared according to *General Procedure **A*** from a mixture of 2,6-di-*tert*-butylphenol (1.2 g, 5.8 mmol), benzaldehyde (0.6 mL, 5.8 mmol) and piperidine (1.2 mL, 11.6 mmol) in PhMe (50 mL) that was heated to reflux in a Dean-Stark apparatus. The reaction mixture was continued to reflux for 18 h before cooling the reaction mixture to below the boiling point. Acetic anhydride (1.1 mL, 11.6 mmol) was added and stirring was continued for 1 h before the workup was performed. The crude material that was purified by flash silica column chromatography (petroleum ether/CH_2Cl_2, 95:5) to give the title compound **5** (1.33 g, 78%) as an orange solid.

mp 52-54 °C; **^{1}H NMR** (400 MHz, $CDCl_3$) δ_H: 1.30 (9H, s, (C(2)C(C*H*$_3$)$_3$), 1.34 (9H, s, (C(6)C(C*H*$_3$)$_3$), 7.02 (1H, d, *J* 2.5, C(3)*H*), 7.19 (1H, s, C(4)=C*H*), 7.36-7.42 (1H, m, C(4′)*H*), 7.44-7.48 (4H, m, C(2')*H*, C(3')*H*, C(5')*H*, C(6')*H*), 7.53 (1H, d, *J* 2.5, C(5)*H*).

Spectroscopic data in accordance with the literature.[2]

2,6-Dimethylphenyl benzoate (S1) *and* (4-Hydroxy-3,5-dimethylphenyl)(phenyl)methanone (S2)

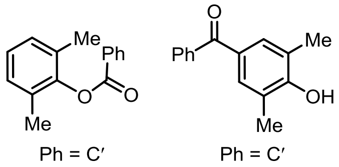

2,6-Dimethylphenol (2.45 g, 20 mmol) was dissolved in PhMe (20 mL) and benzoyl chloride (4.15 mL, 36 mmol) was added dropwise. $AlCl_3$ (3.30 g, 25 mmol) was slowly added and the reaction mixture was heated and stirred at 170 °C for 20 h. The reaction mixture was cooled to rt and then concentrated *in vacuo*. The resulting residue was purified by flash silica column chromatography (petroleum ether/EtOAc, 90:10 to 75:25) to afford predominately the *O*-acylated product **S1** as a yellow oil (3.57 g, 80%) and the desired *C*-acylated product **S2** (0.67 g, 15%) as a yellow solid.

2,6-Dimethylphenyl benzoate (S1): ^{1}H NMR (400 MHz, $CDCl_3$) δ_H: 2.21 (3H, s, C(2)C*H*$_3$), 2.22 (3H, s, C(2)C*H*$_3$), 7.10-7.14 (3H, m, C(3)*H*, C(4)*H*, C(5)*H*), 7.51-7.59 (2H, m, C(3')*H*, C(5')*H*), 7.64-7.70 (1H, m, C(4')*H*), 8.24-8.29 (2H, m, C(2')*H*, C(6')*H*).

(4-Hydroxy-3,5-dimethylphenyl)(phenyl)methanone (S2): mp 116-118 °C; **^{1}H NMR** (400 MHz, $CDCl_3$) δ_H: 2.29 (6H, s, C(2)C*H*$_3$, C(6)C*H*$_3$), 5.12 (1H, s, C(1)O*H*), 7.44-7.50 (2H, m, C(3')*H*, C(5')*H*), 7.52 (2H, s, C(3)*H*, C(5)*H*), 7.52-7.61 (1H, m, C(4')*H*), 7.72- 7.77 (2H, m, C(2)*H*, C(6)*H*).

Spectroscopic data in accordance with the literature.[3]

4-Benzyl-2,6-dimethylphenol (S3)

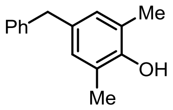

To palladium on carbon (10 wt.%, 0.12 g) under H_2 was added a solution of (4-hydroxy-3,5-dimethylphenyl)(phenyl)methanone **S2** (0.66 g, 3 mmol) in EtOH (5 mL). The reaction mixture was stirred at rt for 18 h before being filtered through a pad of Celite. The filtrate was concentrated *in vacuo* to give a yellow residue which was purified by flash silica column chromatography (petroleum ether/EtOAc, 85:25) to yield the title compound **S3** (0.40 g, 63%) as a yellow solid.

mp 48-50 °C; **^{1}H NMR** (500 MHz, $CDCl_3$) δ_H: 2.23 (6H, s, C(2)C*H*$_3$, C(6)C*H*$_3$), 3.87 (2H, C(4)C*H*$_2$), 4.50 (1H, s, C(1)O*H*), 6.83 (2H, s, C(3)*H*, C(5)*H*), 7.19-7.23 (3H, m, *Ar*), 7.27-7.33 (2H, m, *Ar*).

Spectroscopic data in accordance with the literature.[3]

4-Benzylidene-2,6-dimethylcyclohexa-2,5-dien-1-one (9)

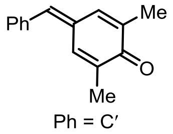

The title compound was prepared according to *General Procedure **B*** from 4-benzyl-2,6-dimethylphenol **S3** (0.40 g, 1.88 mmol), KOH (0.42 g, 7.91 mmol) and $K_3Fe(CN)_6$ (2.48 g, 7.54 mmol) in hexane (10 mL) and H_2O (10 mL). The crude material was purified by flash silica column chromatography (petroleum ether/EtOAc, 90:10) to yield the title compound **9** (72 mg, 18%) as a yellow oil.

^{1}H NMR (400 MHz, $CDCl_3$) δ_H: 2.09 (6H, s, (C(2)C*H*$_3$, (C(6)C*H*$_3$), 7.07 (1H, s C(3)*H*), 7.18 (1H, s, C(4)=C*H*Ph), 7.39-7.45 (1H, m, C(4')*H*), 7.48 (4H, d, *J* 4.5, C(2')*H*, C(3')*H*, C(5')*H*, C(6')*H*), 7.54 (1H, s, C(5)*H*).

Spectroscopic data in accordance with the literature.[3]

2,6-Dibromo-4-(hydroxy(phenyl)methyl)phenol (S4)

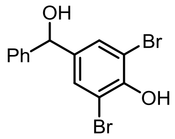

The title compound was prepared according to *General Procedure **C*** from 3,5-dibromo-4-hydroxybenzaldehyde (700 mg, 2.5 mmol) and PhMgBr (2 mL, 5.8 mmol) in anhydrous THF (13 mL). The crude material was purified by flash silica column chromatography (petroleum ether/EtOAc, 80:20) to give the title compound **S4** (769 mg, 86%) as a beige solid.

mp 130-132 °C; **^{1}H NMR** (400 MHz, $CDCl_3$) δ_H: 5.74 (1H, s, C(4)C*H*), 5.85 (1H, s, C(4)CO*H*), 7.28-7.40 (5H, m, *Ar*), 7.50 (2H, d, *J* 0.7, C(3)*H*, C(5)*H*).

Spectroscopic data in accordance with the literature.[2]

2,6-Dibromo-4-(phenyl(phenylsulfonyl)methyl)phenol (S5)

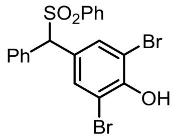

The title compound was prepared according to *General Procedure **D*** from conc. H_2SO_4 (1 mL), 2,6-dibromo-4-(hydroxy(phenyl)methyl)phenol **S4** (500 mg, 1.4 mmol), $PhSO_2Na$ (450 mg, 2.7 mmol) in H_2O (18 mL) and AcOH (18 mL). The reaction mixture was heated to 100 °C for 18 h before being cooled down to rt. The resulting precipitate upon addition of distilled water was filtered and the solid dried under high vacuum to give the title compound **S5** (562 mg, 83%) as a colourless solid.

mp 184-186 °C; **^{1}H NMR** (400 MHz, $CDCl_3$) δ_H: 5.17 (1H, s, C(4)C*H*), 5.98 (1H, s, C(1)O*H*), 7.29 (1H, s, C(3)*H*), C(5)*H*), 7.31-7.40 (2H, m, *Ar*), 7.40-7.50 (4H, m, *Ar*), 7.59 (1H, t, *J* 7.5, *Ar*), 7.62-7.69 (3H, m, *Ar*).

Spectroscopic data in accordance with the literature.[2]

4-Benzylidene-2,6-dibromocyclohexa-2,5-dien-1-one (10)

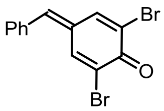

The title compound was prepared according to *General Procedure **D*** from sulfone **S5** (562 mg, 1.16 mmol). The crude material obtained after the workup was purified by flash silica column chromatography (petroleum ether/CH_2Cl_2, 95:5) to give the title compound **10** (220 mg, 56%) as a green solid.

mp > 230 °C; **^{1}H NMR** (400 MHz, $CDCl_3$) δ_H: 7.16-7.42 (6H, m, *Ar*), 7.42-7.73 (3H, m, *Ar*).

Spectroscopic data in accordance with the literature.[2]

4-Hydroxy-3,5-diphenylbenzaldehyde (S6)

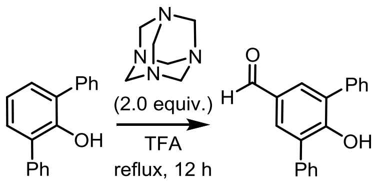

2,6-Diphenylphenol (1.10 g, 4.5 mmol), hexamethylenetetramine (1.26 g, 9.0 mmol) and TFA (4.5 mL) were mixed and heated to reflux (90 °C) for 12 h. The reaction mixture was cooled to rt and neutralised with saturated aqueous $NaHCO_3$. The aqueous layer was extracted with EtOAc (× 3) and the combined organic layers were dried over anhydrous Na_2SO_4, and the filtrate concentrated *in vacuo*. The crude material was purified by recrystallization from Et_2O to give the title compound **S6** (0.71 g, 58%) as a pale yellow solid.

mp 150-152 °C (Et_2O); **^{1}H NMR** (400 MHz, $CDCl_3$) δ_H: 5.98 (1H, s, C(4)O*H*), 5.98 (s, 1H, C(4)O*H*), 7.40-7.48 (2H, m, *Ar*), 7.50-7.54 (4H, m, *Ar*), 7.55-7.60 (4H, m, *Ar*), 7.83 (2H, s, C(2)*H*, C(6)*H*), 9.96 (1H, s, C(1)CO*H*).

Spectroscopic data in accordance with the literature.[2]

2,6-Diphenyl-4-(hydroxy(phenyl)methyl)phenol (S7)

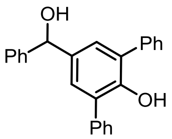

The title compound was prepared according to *General Procedure* ***C*** from 4-hydroxy-3,5-diphenylbenzaldehyde **S6** (686 mg, 2.5 mmol) and PhMgBr (2 mL, 5.8 mmol) in anhydrous THF (13 mL). The crude material was purified by flash silica column chromatography (petroleum ether/EtOAc, 80:20) to give the title compound **S**7 (500 mg, 57%) as a pale yellow oil.

^{1}H NMR (400 MHz, $CDCl_3$) δ_H: 5.43 (1H, s, C(4)O*H*), 5.90 (1H, s, C(4)C*H*), 7.23-7.34 (3H, m, C(3)*H*, C(5)*H*, *Ar*), 7.34-7.44 (4H, m, *Ar*), 7.45-7.53 (6H, m, *Ar*), 7.52-7.61 (4H, m, *Ar*).

Spectroscopic data in accordance with the literature.[2]

5'-(Phenyl(phenylsulfonyl)methyl)-[1,1':3',1''-terphenyl]-2'-ol (S8)

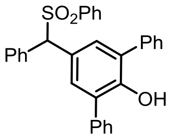

The title compound was prepared according to *General Procedure* ***D*** from conc. H_2SO_4 (1 mL), 2,6-diphenyl-4-(hydroxy(phenyl)methyl)phenol **S7** (300 mg, 0.85 mmol), $PhSO_2Na$ (272 mg, 1.64 mmol) in H_2O (11 mL) and AcOH (11 mL). The reaction mixture was heated to 100 °C for 18 h before being cooled down to rt. The resulting precipitate upon addition of distilled water was filtered and the solid dried under high vacuum to yield the title compound **S8** (305 mg, 75%) as a pink solid.

mp 150-152 °C; **^{1}H NMR** (400 MHz, $CDCl_3$) δ_H: 5.32 (1H, s, C(4)C*H*), 7.28- 7.38 (3H, m, *Ar*), 7.39-7.53 (14H, m, *Ar*), 7.54-7.64 (3H, m, *Ar*), 7.70 (2H, d, *J* 7.7, *Ar*).

Spectroscopic data in accordance with the literature.[2]

5'-Benzylidene-[1,1':3',1''-terphenyl]-2'(5'H)-one (11)

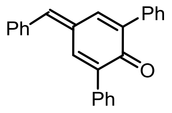

The title compound was prepared according to *General Procedure* ***D*** from sulfone **S8** (334 mg, 0.70 mmol). The crude material obtained after the workup was purified by flash silica

column chromatography (petroleum ether/CH_2Cl_2, 95:5) to give the title compound **11** (68 mg, 29%) as a yellow solid.

mp 120-122 °C; **^{1}H NMR**(400 MHz, $CDCl_3$) δ_H: 7.40-7.53 (11H, m, *Ar*), 7.55-7.76 (6H, m, *Ar*), 7.92 (1H, d, *J* 2.5, C(5)*H*).

Spectroscopic data in accordance with the literature.[2]

2,6-Di-*tert*-butyl-4-ethylidenecyclohexa-2,5-dien-1-one (12)

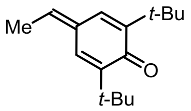

The title compound was prepared according to *General Procedure* ***B*** from 2,6-di-tert-butyl-4-ethylphenol (1.80 g, 7.7 mmol), KOH (1.73 g, 30.9 mmol) and $K_3Fe(CN)_6$ (10.14 g, 30.8 mmol) in hexane (28 mL) and H_2O (28 mL). The crude material was purified by flash silica column chromatography (petroleum ether/EtOAc, 90:10) to yield the title compound **12** (1.50 g, 84%) as a green solid.

mp 68-70 °C; **^{1}H NMR** (400 MHz, $CDCl_3$) δ_H: 1.22 (9H, s, (C(2)C(C*H*$_3$)$_3$), 1.25 (9H, s, (C(6)C(C*H*$_3$)$_3$), 2.04 (3H, d, *J* 7.7, C(4)=CHC*H*$_3$), 6.34 (1H, q, *J* 7.7, C(3)*H*), 6.78 (1H, d, *J* 2.5, C(4)=C*H*CH$_3$), 7.24 (1H, d, *J* 2.5, C(5)*H*).

Spectroscopic data in accordance with the literature.[3]

2,6-Di-tert-butyl-4-(naphthalen-2-ylmethylene)cyclohexa-2,5-dien-1-one (13)

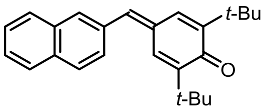

The title compound was prepared according to *General Procedure* ***A*** from a mixture of 2,6-di-*tert*-butylphenol (1.2 g, 5.8 mmol), 2-naphthaldehyde (0.91 g, 5.8 mmol) and piperidine (1.2 mL, 11.6 mmol) in PhMe (50 mL) that was heated to reflux in a Dean-Stark apparatus. The reaction mixture was continued to reflux for 18 h before cooling the reaction mixture to below the boiling point. Acetic anhydride (1.1 mL, 11.6 mmol) was added and stirring was continued for 1 h before the workup was performed. The crude material that was purified by flash silica column chromatography (petroleum ether/CH_2Cl_2, 95:5) to give the title compound **13** (1.45 g, 73%) as an orange solid.

mp 76-78 °C; **^{1}H NMR** (400 MHz, $CDCl_3$) δ_H: 1.34 (9H, s, (C(2)C(CH_3)$_3$), 1.38 (9H, s, (C(6)C(CH_3)$_3$), 7.11 (1H, d, *J* 2.3, C(3)*H*), 7.37 (1H, s, C(4)=C*H*), 7.54-7.62 (3H, m, *Ar*), 7.67 (1H, d, *J* 2.3, C(5)*H*), 7.86-7.98 (4H, m, *Ar*).

Spectroscopic data in accordance with the literature.[2]

Synthesis of Aryl Esters

4'-Nitrophenyl 2-(*p*-tolyl)acetate (1)

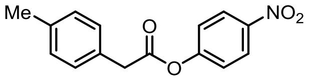

The title compound was prepared according to *General Procedure* **E** from *p*-tolyl acetic acid (1.25 g, 8.63 mmol), EDCI·HCl (1.20 g, 6.47 mmol) and 4-nitrophenol (0.60 g, 4.31 mmol) in CH_2Cl_2 (14 mL) to give crude material that was purified by flash silica column chromatography (hexane/EtOAc, 85:15) to give **1** (0.93 g, 79%) as a colourless crystalline solid.

mp 60-62 °C; **^{1}H NMR** (400 MHz, $CDCl_3$) δ_H: 2.42 (3H, s, C*H₃*), 3.92 (2H, s, C(2)*H₂*), 7.25 (2H, d, *J* 7.9, C(3'')*H*, C(5'')*H*), 7.29-7.35 (4H, m, C(2')*H*, C(6')*H*, C(3'')*H*, C(5'')*H*), 8.30 (2H, d, *J* 9.2, C(3')*H*, C(5')*H*).

Spectroscopic data in accordance with the literature.[4]

Perfluorophenyl 2-(*p*-tolyl)acetate (2)

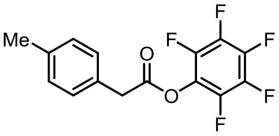

The title compound was prepared according to *General Procedure* **E** from *p*-tolyl acetic acid (10.1 g, 67.0 mmol), EDCI·HCl (9.63 g, 50.3 mmol) and 2,3,4,5,6-pentafluorophenol (6.17 g, 33.5 mmol) in CH_2Cl_2 (56 mL) to give crude material that was purified by flash silica column chromatography (CH_2Cl_2) to give **2** (6.88 g, 65%) as a colourless solid.

mp 27-29 °C; **^{1}H NMR** (400 MHz, $CDCl_3$) δ_H: 2.40 (3H, s, CH_3), 3.96 (2H, s, CH_2), 7.22 (2H, d, *J* 7.9, C(3')*H*, C(5')*H*), 7.28 (2H, d, *J* 8.1, C(2')*H*, C(6')*H*); **^{19}F{^{1}H} NMR** (376 MHz, $CDCl_3$) δ_F: –162.5 - –162.3 (m), –157.9 (t, *J* 21.7), –152.7 - –152.6 (m).

Spectroscopic data in accordance with the literature.[5]

3',5'-Bis(trifluoromethyl)phenyl 2-(*p*-tolyl)acetate (3)

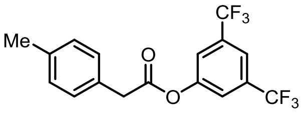

The title compound was prepared according to *General Procedure* **E** from *p*-tolyl acetic acid (2.50 g, 16.7 mmol), EDCI·HCl (2.40 g, 12.5 mmol) and 3,5-bis(trifluoromethyl)phenol (1.92

g, 8.33 mmol) in CH_2Cl_2 (30 mL) to give crude material that was purified by flash silica column chromatography (hexane/EtOAc, 95:5) to give **3** (2.84 g, 94%) as a colourless solid.

mp 57-58 °C; **^{1}H NMR** (500 MHz, $CDCl_3$) δ_H: 2.40 (3H, s, C*H$_3$*), 3.90 (2H, s, C(2)*H$_2$*), 7.24 (2H, d, *J* 7.9, C(2")*H*, C(6")*H*), 7.30 (2H, d, *J* 7.9, C(3")*H*, C(5")*H*), 7.61 (2H, d, *J* 1.8, *Ar*), 7.78 (1H, s, *Ar*).

Spectroscopic data in accordance with the literature.[4]

2',4',6'-Trichlorophenyl 2-(*p*-tolyl)acetate (4)

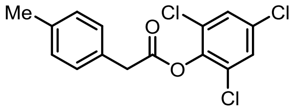

The title compound was prepared according to *General Procedure* ***E*** from *p*-tolyl acetic acid (2.50 g, 16.7 mmol), EDCI·HCl (2.40 g, 12.5 mmol) and trichlorophenol (1.60 g, 8.33 mmol) in CH_2Cl_2 (29 mL) to give crude material that was purified by flash silica column chromatography (hexane/EtOAc, 95:5) to give **4** (2.47 g, 91%) as a colourless solid.

mp 49-51 °C; **^{1}H NMR** (500 MHz, $CDCl_3$) δ_H: 2.39 (3H, s, C*H$_3$*), 3.96 (2H, s, C(2)*H$_2$*), 7.21 (2H, d, *J* 7.8, C(3")*H*, C(5")*H*), 7.32 (2H, d, *J* 7.8, C(2")*H*, C(6")*H*), 7.38 (2H, s, *Ar*).

Spectroscopic data in accordance with the literature.[4]

4'-Nitrophenyl 2-(4"-methoxyphenyl)acetate (21)

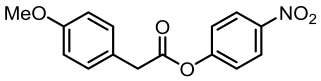

The title compound was prepared according to *General Procedure* ***E*** from 2-(4-methoxyphenyl)acetic acid (1.55 g, 9.34 mmol), EDCI·HCl (1.30 g, 7.01 mmol) and 4-nitrophenol (0.65 g, 4.67 mmol) in CH_2Cl_2 (14 mL) to give crude material that was purified by flash silica column chromatography (hexane/EtOAc, 85:15) to give **21** (0.93 g, 69%) as a pale yellow crystalline solid.

mp 88-90 °C; **^{1}H NMR** (400 MHz, $CDCl_3$) δ_H: 3.84 (3H, s, C*H$_3$*), 3.86 (2H, s, C(2)*H$_2$*), 6.94 (2H, d, *J* 8.8, C(3")*H*, C(5")*H*), 7.28 (2H, *J* 9.2, C(2')*H*, C(6')*H*), 7.31 (2H, d, *J* 8.8, C(2")*H*, C(6")*H*), 8.27 (2H, d, *J* 9.2, C(3')*H*, C(5')*H*).

Spectroscopic data in accordance with the literature.[4]

4'-Nitrophenyl 2-(4''-(trifluoromethyl)phenyl)acetate (22)

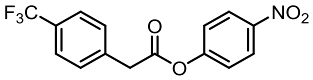

The title compound was prepared according to *General Procedure* ***E*** from 2-(4-(trifluoromethyl)phenyl)acetic acid (1.91 g, 9.34 mmol), EDCI·HCl (1.30 g, 7.01 mmol) and 4-nitrophenol (0.65 g, 4.67 mmol) in CH_2Cl_2 (14 mL) to give crude material that was purified by flash silica column chromatography (hexane/EtOAc, 85:15) to give **22** (0.96 g, 64%) as a colourless solid.

mp 65-67 °C; **^{1}H NMR** (400 MHz, $CDCl_3$) δ_H: 4.00 (2H, s, C(2)H_2), 7.30 (2H, d, *J* 9.2, C(2')*H*, C(6')*H*), 7.53 (2H, d, *J* 8.0, C(2'')*H*, C(6'')*H*), 7.68 (2H, d, *J* 8.0, C(3'')*H*, C(5'')*H*), 8.29 (2H, d, *J* 9.2, C(3')*H*, C(5')*H*); **^{19}F{^{1}H} NMR** (376 MHz, $CDCl_3$) δ_F: 62.6 (CF_3).

Spectroscopic data in accordance with the literature.[4]

4'-Nitrophenyl 2-(naphthalen-2''-yl)acetate (23)

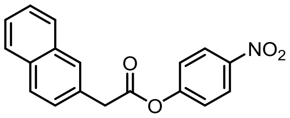

The title compound was prepared according to *General Procedure* ***E*** from 2-(naphthalen-2-yl)acetic acid (1.74 g, 9.34 mmol), EDCI·HCl (1.30 g, 7.01 mmol) and 4-nitrophenol (0.65 g, 4.67 mmol) in CH_2Cl_2 (14 mL) to give crude material that was purified by flash silica column chromatography (hexane/EtOAc, 85:15) to give **23** (0.95 g, 66%) as a colourless crystalline solid.

mp 94-96 °C; **^{1}H NMR** (400 MHz, $CDCl_3$) δ_H: 4.09 (2H, s, C(2)H_2), 7.29 (2H, d, *J* 9.2, C(2')*H*, C(6')*H*), 7.46-7.63 (3H, m, *Ar*), 7.82-8.00 (4H, m, *Ar*), 8.27 (2H, d, *J* 9.2, C(3')*H*, C(5')*H*).

Spectroscopic data in accordance with the literature.[4]

4'-Nitrophenyl 2-(naphthalen-1''-yl)acetate (24)

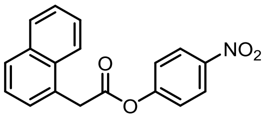

The title compound was prepared according to *General Procedure* ***E*** from 2-(naphthalen-1-yl)acetic acid (1.74 g, 9.34 mmol), EDCI·HCl (1.30 g, 7.01 mmol) and 4-nitrophenol (0.65 g,

4.67 mmol) in CH_2Cl_2 (14 mL) to give crude material that was purified by flash silica column chromatography (hexane/EtOAc, 85:15) to give **24** (0.87 g, 61%) as a colourless crystalline solid.

mp 79-81 °C; **^{1}H NMR** (400 MHz, $CDCl_3$) δ_H: 4.37 (2H, s, C(2)*H₂*), 7.22 (2H, d, *J* 9.0, C(2')*H*, C(6')*H*), 7.46-7.67 (4H, m, *Ar*), 7.89 (1H, d, *J* 7.9, C(8")*H*), 7.94 (1H, d, *J* 7.9, C(5")*H*), 8.10 (1H, d, *J* 8.4, C(4")*H*), 8.23 (2H, d, *J* 9.1, C(3')*H*, C(5')*H*).

Spectroscopic data in accordance with the literature.[4]

4'-Nitrophenyl 2-(thiophen-3"-yl)acetate (25)

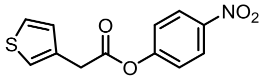

The title compound was prepared according to *General Procedure* ***E*** from 2-(thiophen-3-yl)acetic acid (1.33 g, 9.34 mmol), EDCI·HCl (1.30 g, 7.01 mmol) and 4-nitrophenol (0.65 g, 4.67 mmol) in CH_2Cl_2 (14 mL) to give crude material that was purified by flash silica column chromatography (hexane/EtOAc, 85:15) to give **25** (0.76 g, 61%) as a colourless solid.

mp 55-57 °C; **^{1}H NMR** (400 MHz, $CDCl_3$) δ_H: 3.97 (2H, s, C(2)H_2), 7.15 (1H, dd, *J* 5.0, 1.3, C(4")*H*), 7.28-7.33 (3H, m, C(2')*H*, C(6')*H*, C(2")*H*), 7.39 (1H, dd, *J* 5.0, 3.0, C(5")*H*), 8.29 (2H, d, *J* 9.2, C(3')*H*, C(5')*H*).

Spectroscopic data in accordance with the literature.[4]

Amide Products Derived from Isothiourea Catalysis

Only the structure of the major diastereoisomer is shown in each case.

(2*S*,1′*R*)-*N*-Benzyl-1′-(3′′′′,5′′′′-di-*tert*-butyl-4′′′′-hydroxyphenyl)-2-(*p*-tolyl)-1′-phenyl propenamide (7) (Table 1, entry 8)

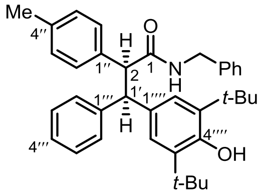

By following *General Procedure F*, 4-benzylidene-2,6-di-*tert*-butylcyclohexa-2,5-dien-1-one **5** (74 mg, 0.25 mmol), 4'-nitrophenyl 2-(*p*-tolyl)acetate **1** (102 mg, 0.375 mmol), (*S*)-TM·HCl (3 mg, 5 mol%) and Et_3N (35 µL, 0.25 mmol) were dissolved in anhydrous MeCN (0.42 mL). The reaction mixture was stirred at 40 °C for 24 h before being quenched with benzylamine (137 µL, 1.25 mmol) at rt to give a crude mixture containing the title compound in 60:40 dr. The mixture was purified by flash silica column chromatography (petroleum ether/EtOAc, 85:15) to afford the title compound **7** (132 mg, 99%) as a pale yellow solid in 65:35 dr.

mp 126-128 °C; $[\alpha]_D^{20}$ +10.0 (*c* 1.0, $CHCl_3$); **Chiral HPLC analysis**,Chiralpak AD-H (10% *i*-PrOH/hexane, flow rate 1.5 $mLmin^{-1}$, 211 nm, 40 °C), t_R major: 8.5 min and 29.3 min, 92:8 er, t_R minor: 3.8 min and 18.6 min, 85:15 er ; **IR** ν_{max} (film)/cm^{-1} 3638 (O–H) 3304 (N–H), 2955 (C–H), 1645 (C=O); **^{1}H NMR** (500 MHz, $CDCl_3$) δ_H: 1.42 (18H, s, (C(3'''')C(C*H*$_3$)$_3$, C(5'''')C(C*H*$_3$)$_3$), 2.24 (3H, s, C(4'')C*H*$_3$), 3.90-4.05 (2H, m, C(2)*H*, C*H*$_A$Ph), 4.44 (1H, dd, *J* 15.0, 6.8, C*H*$_B$Ph), 4.82 (1H, d, *J* 11.7, C(1')*H*), 5.14 (1H, s, O*H*), 5.55 (1H, t, *J* 5.6, N*H*), 6.71-6.76 (2H, m, *Ar*), 6.96-7.05 (2H, m, *Ar*), 7.09-7.15 (2H, m, *Ar*), 7.15-7.22 (4H, m, *Ar*), 7.23-7.30 (4H, m, C(4''')*H*, C(2'''')*H*, C(6'''')*H*, *Ar*), 7.34 (1H, t, *J* 7.6, *Ar*), 7.43-7.51 (1H, m, *Ar*); **^{13}C{^{1}H} NMR** (126 MHz, $CDCl_3$) δ_C: 21.0 (C(4'')*C*H$_3$), 30.4 (C(3'''')C(*C*H$_3$)$_3$, C(5'''')C(*C*H$_3$)$_3$), 34.4 (C(3'''')*C*(CH$_3$)$_3$, C(5'''')*C*(CH$_3$)$_3$), 43.6 (*C*H$_2$Ph), 54.1 (*C*(1')), 59.5 (*C*(2)), 124.7 (*C*(2''''), *C*(6'''')), 125.8 (*C*(4''')), 128.2 (*Ar*), 127.2 (*Ar*), 127.3 (*Ar*), 128.0 (*Ar*), 128.4 (*Ar*), 128.5 (*Ar*), 128.6 (*Ar*), 129.0 (*Ar*), 133.8 (*C*(1''')), 135.2 (*C*(1'')), 135.6 (*C*(3''''), *C*(5'''')), 136.4 (*C*(4'')), 137.9 (*i-Ph*), 142.4 (*C*(1'''')), 152.4 (*C*(4'''')), 172.1 (*C*(1)); **HRMS** (ESI^+) $C_{37}H_{43}NO_2$ ($[M+H]^+$), found 534.3359, requires 534.3367 (−1.4 ppm).

Selected data for minor diastereoisomer (**8**): **^{1}H NMR** (500 MHz, $CDCl_3$) δ_H: 1.27 (18H, s, (C(3'''')C(C*H*$_3$)$_3$, C(5'''')C(C*H*$_3$)$_3$), 2.27 (3H, s, C(4'')C*H*$_3$), 3.90-4.05 (2H, m, C(2)*H*, C*H*$_A$Ph), 4.50 (1H, dd, *J* 15.0, 6.8, C*H*$_B$Ph), 4.69 (1H, d, *J* 11.7, C(1')*H*), 4.89 (1H, s, O*H*), 5.69 (1H, t,

J 5.6, N*H*), 6.81-6.84 (2H, m, *Ar*); **^{13}C{^{1}H} NMR** (126 MHz, $CDCl_3$) δ_C: 21.0 (C(4")*C*H_3), 30.2 (C(3"")C(*C*H_3)$_3$, C(5"")C(*C*H_3)$_3$), 34.2 (C(3"")*C*(CH_3)$_3$, C(5"")*C*(CH_3)$_3$), 43.4 (*C*H_2Ph), 54.7 (*C*(1')), 59.5 (*C*(2)), 125.3 (*C*(2""), *C*(6"")), 126.3 (*C*(4"')), 127.5 (*Ar*), 128.2 (*Ar*), 128.4 (*Ar*), 128.8 (*Ar*), 132.1 (*C*(1"')), 134.9 (*C*(3""), *C*(5"")), 135.5 (*C*(1")), 136.4 (*C*(4")), 138.2 (*i-Ph*), 143.6 (*C*(1""), 151.7 (*C*(4"")), 172.0 (*C*(1)).

(2*S*,1′*R*)-*N*-Benzyl-1′-(3′′′′,5′′′′-di-*tert*-butyl-4′′′′-hydroxyphenyl)-2-(*p*-tolyl)-1′-phenyl pentanamide (17)

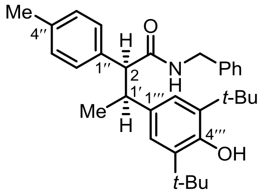

By following *General Procedure **F***, 2,6-di-*tert*-butyl-4-ethylidenecyclohexa-2,5-dien-1-one **12** (58 mg, 0.25 mmol), 4'-nitrophenyl 2-(*p*-tolyl)acetate **1** (102 mg, 0.375 mmol), (*S*)-TM·HCl (3 mg, 5 mol%) and Et_3N (35 µL, 0.25 mmol) were dissolved in anhydrous MeCN (0.42 mL). The reaction mixture was stirred at 40 °C for 24 h before being quenched with benzylamine (137 µL, 1.25 mmol) at rt to give a crude mixture containing the title compound in 60:40 dr. The mixture was purified by flash silica column chromatography (petroleum ether/EtOAc, 85:15) to afford the title compound **17** (59 mg, 50%) as a pale yellow solid in 65:35 dr.

mp 56-58 °C; $[\alpha]_D^{20}$ +18.7 (*c* 1.0, $CHCl_3$); **Chiral HPLC analysis**, Chiralpak AD-H (5% *i*-PrOH/hexane, flow rate 1.5 mLmin^{-1}, 211 nm, 40 °C), t_R major: 5.9 min and 11.8 min, 89:11 er, t_R minor: 4.8 min and 14.2 min, 81:19 er; **IR** ν_{max} (film)/cm^{-1} 3649 (O–H) 3285 (N–H), 2957 (C–H), 1643 (C=O); **^{1}H NMR** (400 MHz, $CDCl_3$) δ_H: 1.34 (18H, s, (C(3"')C(C*H_3*)$_3$, C(5"')C(C*H_3*)$_3$), 1.45-1.50 (3H, m, C(1')C*H_3*), 2.26 (3H, s, C(4")C*H_3*), 3.21 (1H, d, *J* 10.4, C(2)*H*), 3.44-3.53 (1H, m, C(1')*H*), 4.30 (1H, dd, *J* 14.8, 5.2, C*H_A*Ph), 4.62 (dd, *J* = 14.8, 6.2, C*H_B*Ph), 4.97 (1H, s, O*H*), 6.02 (1H, t, *J* 5.6, N*H*), 6.70 (2H, s, *Ar*), 6.91-7.00 (2H, m, *Ar*), 7.09-7.15 (2H, m, *Ar*), 7.16-7.24 (4H, m, C(2"')*H*, C(6"')*H*, *Ar*), 7.26-7.35 (3H, m, *Ar*); **^{13}C{^{1}H} NMR** (101 MHz, $CDCl_3$) δ_C: 19.8 (C(1')*C*H_3), 20.9 (C(4")*C*H_3), 30.3 (C(3"')C(*C*H_3)$_3$, C(5"')C(*C*H_3)$_3$), 34.2 (C(3"')*C*(CH_3)$_3$, C(5"')*C*(CH_3)$_3$), 43.5 (*C*(1')), 43.7 (*C*H_2Ph), 61.9 (*C*(2)), 124.4 (*C*(2"'), *C*(6"')), 127.2 (*Ar*), 127.4 (*Ar*), 127.7 (*Ar*), 128.3 (*Ar*), 128.5 (*Ar*), 128.6 (*Ar*), 128.7 (*Ar*), 129.1 (*Ar*), 135.0 (*C*(3"'), *C*(5"')), 135.6 (*C*(1"')), 136.1 (*C*(1")), 136.1 (*C*(4")), 138.4 (*i-Ph*), 151.8 (*C*(4"')), 173.1 (*C*(1)); **HRMS** (NSI$^+$) $C_{32}H_{42}NO_2$ ([M+H]$^+$), found 472.3210, requires 472.3201 (−1.9 ppm).

Selected data for minor diastereoisomer: **^{1}H NMR** (400 MHz, $CDCl_3$) δ_H: 1.10 (3H, d, *J* 7.0, C(1')CH_3), 1.48 (18H, s, (C(3''')C(CH_3)$_3$, C(5''')C(CH_3)$_3$), 2.39 (3H, s, C(4'')CH_3), 3.28 (1H, d, *J* 10.4, C(2)*H*), 3.54-3.61 (1H, m, C(1')*H*), 3.87 (1H, dd, *J* 15.0, 4.1, CH_APh), 4.45 (1H, dd, *J* 15.0, 7.0, CH_BPh), 5.23 (1H, s, O*H*), 5.53 (1H, t, *J* 5.6, N*H*), 6.64-6.69 (2H, m, *Ar*), 7.40-7.49 (2H, m, *Ar*); **^{13}C{^{1}H} NMR** (101 MHz, $CDCl_3$) δ_C: 19.6 (C(1')*C*H$_3$), 21.1 (C(4'')*C*H$_3$), 30.5 (C(3''')C(*C*H$_3$)$_3$, C(5''')C(*C*H$_3$)$_3$), 34.5 (C(3''')*C*(CH$_3$)$_3$, C(5''')*C*(CH$_3$)$_3$), 42.9 (*C*(1')), 43.3 (*C*H$_2$Ph), 62.5 (*C*(2)), 124.1 (*C*(2'''), *C*(6''')), 127.1 (*Ar*), 132.1 (*Ar*), 134.9 (*C*(3'''), *C*(5''')), 134.0 (*C*(1'')), 135.7 (*Ar*), 135.8 (*Ar*), 134.0 (*C*(3''), *C*(5'')), 135.7 (*C*(1''')), 135.8 (*C*(1'')), 136.8 (*C*(4'')), 138.0 (*i-Ph*), 152.4 (*C*(4''')), 172.6 (*C*(1)).

(2*S*,1′*R*)-*N*-Benzyl-1′-(3′′′′,5′′′′-di-*tert*-butyl-4′′′′-hydroxyphenyl)-2-(*p*-tolyl)-1′-(naphthalen-2′′-yl)-propenamide (18)

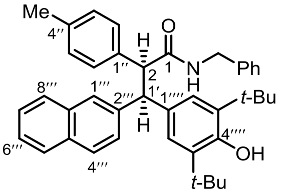

By following *General Procedure* ***F***, 2,6-di-tert-butyl-4-(naphthalen-2-ylmethylene)cyclohexa-2,5-dien-1-one **13** (86 mg, 0.25 mmol), 4'-nitrophenyl 2-(*p*-tolyl)acetate **1** (102 mg, 0.375 mmol), (*S*)-TM·HCl (3 mg, 5 mol%) and Et_3N (35 µL, 0.25 mmol) were dissolved in anhydrous MeCN (0.42 mL). The reaction mixture was stirred at 40 °C for 24 h before being quenched with benzylamine (137 µL, 1.25 mmol) at rt to give a crude mixture containing the title compound in 70:30 dr. The mixture was purified by flash silica column chromatography (petroleum ether/EtOAc, 85:15) to afford the title compound **18** (121 mg, 83%) as a pale yellow solid in > 95:5 dr as the major diastereoisomer.

mp 78-80 °C; $[\alpha]_D^{20}$ +22.8 (*c* 0.5, $CHCl_3$); **Chiral HPLC analysis**, Chiralpak AD-H (10% *i*-PrOH/hexane, flow rate 1.5 mLmin^{-1}, 211 nm, 40 °C), t_R major: 11.9 min and 17.6 min, 94:6 er, t_R minor: 2.2 min and 5.1 min, 63:37 er; **IR** ν_{max} (film)/cm^{-1} 3630 (O–H) 3287 (N–H), 2953 (C–H), 1641 (C=O); **^{1}H NMR** (500 MHz, $CDCl_3$) δ_H: 1.27 (18H, s, (C(3'''')C(CH_3)$_3$, C(5'''')C(CH_3)$_3$), 2.28 (3H, s, C(4'')CH_3), 3.84 (1H, dd, *J* 14.9, 3.7, CH_APh), 4.10 (1H, d, *J* 11.6, C(2)*H*), 4.47 (1H, dd, *J* 14.9, 7.3, CH_BPh), 4.88 (1H, d, *J* 11.8, C(1')*H*), 4.90 (1H, s, O*H*), 5.83 (1H, broad-s, N*H*), 6.60 (2H, d, *J* 7.4, *o*-Ph), 6.81 (2H, s, C(2'''')*H*, C(6'''')*H*), 6.86 (2H, t, *J* 7.7, *m*-Ph), 7.00 (2H, d, *J* 7.6, C(3′′)*H*, C(5′′)*H*), 7.06 (1H, t, *J* 7.3, *p*-Ph), 7.18 (2H, d, *J* 7.7, C(2′′)*H*, C(6′′)*H*), 7.45-7.51 (2H, m, *Ar*), 7.57 (1H, d, *J* 8.2, *Ar*), 7.78 (1H, d, *J* 8.5, *Ar*), 7.79-7.86 (2H, m, *Ar*), 7.94 (1H, s, *Ar*). **^{13}C{^{1}H} NMR** (126 MHz, $CDCl_3$) δ_C: 21.1

(C(4")*C*H_3), 30.3 (C(3"")C(*C*H_3)$_3$, C(5"")C(*C*H_3)$_3$), 34.2 (C(3"")*C*(CH_3)$_3$, C(5"")*C*(CH_3)$_3$), 43.4 (*C*H_2Ph), 54.8 (*C*(1')), 59.4 (*C*(2)), 125.5 (*C*(2""), *C*(6"")), 126.0 (*Ar*), 126.3 (*Ar*), 127.1 (*p*-Ph), 127.3 (*Ar*), 127.4 (*o*-Ph), 127.7 (*Ar*), 128.1 (*Ar*), 128.3 (*m*-Ph), 128.4 (Ar), 128.6 (Ar), 129.0 (*C*(3″), *C*(5″)), 132.0 (*Ar*), 132.5 (*Ar*), 133.8 (*Ar*), 135.1 (*C*(3""), *C*(5"")), 135.6 (*C*(1")), 136.6 (*C*(4")), 138.1 (*i-Ph*), 141.3 (*C*(1"")), 151.9 (*C*(4"")), 172.2 (*C*(1)). **HRMS** (NSI$^+$) $C_{41}H_{46}NO_2$ ([M+H]$^+$), found 584.3515, requires 584.3523 (−1.4 ppm).

PNP Ester Products from Isothiourea Catalysis

Only the structure of the major diastereoisomer is shown in each case.

(2*S*,1′*R*)-4-Nitrophenyl 1′-(3′′′′,5′′′′-di-*tert*-butyl-4′′′′-hydroxyphenyl)-2-(*p*-tolyl)-1′-phenylpropanoate propanoate (19)

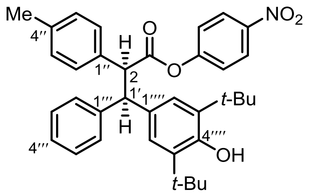

By following *General Procedure* ***G***, 4-benzylidene-2,6-di-*tert*-butylcyclohexa-2,5-dien-1-one **5** (370 mg, 1.25 mmol), 4'-nitrophenyl 2-(*p*-tolyl)acetate **1** (510 mg, 1.88 mmol), (*S*)-TM·HCl (15 mg, 5 mol%) and Et_3N (175 µL, 1.25 mmol) were dissolved in anhydrous MeCN (2.1 mL). The reaction mixture was stirred at rt for 24 h before being concentrated *in vacuo*. The crude material (55:45 dr) was purified and the diastereoisomers separated by flash silica column chromatography (petroleum ether/EtOAc, 95:5). The diastereoisomers were obtained as pale yellow solids (combined: 601 mg, 85%).

mp 83-85 °C; **Chiral HPLC analysis**, Chiralpak AD-H (2% *i*-PrOH/hexane, flow rate 1.5 $mLmin^{-1}$, 211 nm, 30 °C), t_R major: 12.9 min and 23.6 min, 92:8 er, t_R minor: 20.5 min and 35.1 min, 87:13 er; **IR** ν_{max} (film)/cm^{-1} 3615 (O–H), 2953 (C–H), 1769 (C=O), 1522 (N-O), 1489 (C=C), 1346 (N-O), 1109 (C-O); **HRMS** ($ASAP^-$) $C_{36}H_{39}NO_5$ ($[M]^-$), found 565.2822, requires 565.2834 (−2.1 ppm).

Data for major diastereoisomer (19): $[\alpha]_D^{20}$ +109 (*c* 0.1, $CHCl_3$); **^{1}H NMR** (500 MHz, $CDCl_3$) δ_H: 1.28 (18H, s, (C(3'''')C(C*H*$_3$)$_3$, C(5'''')C(C*H*$_3$)$_3$), 2.32 (3H, s, C(4'')C*H*$_3$), 4.48 (1H, d, *J* 12.1, C(2)*H*), 4.59 (1H, d, *J* 12.1, C(1')*H*), 4.97 (1H, s, O*H*), 6.74 (2H, d, *J* 8.8, ester C(1)OC(2)*H*, ester C(1)OC(6)*H*), 6.75 (2H, s, C(2'''')*H*, C(6'''')*H*), 7.08 (2H, d, *J* 7.5, C(3'')*H*, C(5'')*H*), 7.19 (2H, d, *J* 8.1, C(2'')*H*, C(6'')*H*), 7.31 (1H, d, *J* 7.5, C(4''')*H*), 7.41 (2H, t, *J* 7.5, C(3''')*H*, C(5''')*H*), 7.55 (2H, d, *J* 6.8, C(2''')*H*, C(6''')*H*), 8.13 (2H, d, *J* 8.8, ester C(3)*H*, ester C(5)*H*); **^{13}C{^{1}H} NMR** (126 MHz, $CDCl_3$) δ_C: 21.3 (C(4'')*C*H$_3$), 30.6 (C(3'''')C(*C*H$_3$)$_3$, C(5'''')C(*C*H$_3$)$_3$), 34.3 (C(3'''')*C*(CH$_3$)$_3$, C(5'''')*C*(CH$_3$)$_3$), 55.8 (*C*(1')), 57.8 (*C*(2)), 122.5 (ester *C*(2), ester *C*(6)), 125.1 (ester *C*(3), ester *C*(5)), 125.2 (*C*(2''''), *C*(6'''')), 127.0 (*C*(4''')), 128.3 (*C*(2'''), *C*(6''')), 128.6 (*C*(3'''), *C*(5''')), 128.9 (*C*(2''), *C*(6'')), 129.4 (*C*(3''), *C*(5'')), 131.1 (*C*(1''''), 133.3 (*C*(1'')), 135.4 (*C*(3''''), *C*(5'''')), 137.5 (*C*(4'')), 142.6 (*C*(1''')), 145.4 (ester C(1)O*C*(4)NO$_2$), 152.2 (*C*(4'''')), 155.4 (ester C(1)O*C*(1)), 171.0 (*C*(1)O).

Data for minor diastereoisomer (20): $[\alpha]_D^{20}$ +30.0 (*c* 0.1, $CHCl_3$); **^{1}H NMR** (500 MHz, $CDCl_3$) δ_H: 1.45 (18H, s, (C(3'''')C(C*H*$_3$)$_3$, C(5'''')C(C*H*$_3$)$_3$), 2.30 (3H, s, C(4'')C*H*$_3$), 4.65 (2H, s, C(2)*H*, C(1')*H*), 5.20 (1H, s, O*H*), 6.56 (2H, d, *J* 9.0, ester C(1)OC(2)*H*, ester C(1)OC(6)*H*), 7.06-7.11 (3H, m, C(3'')*H*, C(5'')*H*, *C*(4''')*H*), 7.14-7.23 (4H, m, C(2''), C(6''), C(3''')*H*, C(5''')*H*), 7.31-7.37 (4H, m, C(3'')*H*, C(5'')*H*, C(2'''')*H*, C(6'''')*H*), 8.11 (2H, d, *J* 9.0, ester C(3)*H*, ester C(5)*H*); **^{13}C{^{1}H} NMR** (126 MHz, $CDCl_3$) δ_C: 21.3 (C(4'')*C*H$_3$), 30.3 (C(3'''')C(*C*H$_3$)$_3$, C(5'''')C(*C*H$_3$)$_3$), 34.3 (C(3'''')*C*(CH$_3$)$_3$, C(5'''')*C*(CH$_3$)$_3$), 55.5 (*C*(1')), 57.4 (*C*(2)), 122.4 (ester *C*(2), ester *C*(6)), 124.6 (ester *C*(3), ester *C*(5)), 125.2 (*C*(2''''), *C*(6'''')), 126.3 (*C*(4''')), 128.0 (*C*(2'''), *C*(6''')), 128.3 (*C*(3'''), *C*(5''')), 128.6 (*C*(2''), *C*(6'')), 129.4 (*C*(3''), *C*(5'')), 132.8 (*C*(1''''), *C*(1'')), 135.4 (*C*(3''''), *C*(5'''')), 137.4 (*C*(4'')), 141.5 (*C*(1''')), 145.2 (ester C(1)O*C*(4)NO$_2$), 152.2 (*C*(4'''')), 155.4 (ester C(1)O*C*(1)), 171.0 (*C*(1)O).

(2*S*,1′*R*)-4-Nitrophenyl 1-(3′′′′,5′′′′-di-*tert*-butyl-4′′′′-hydroxyphenyl)-2-(4-methoxyphenyl)-1′-phenylpropanoate (26)

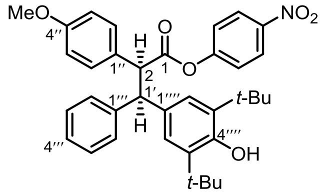

By following *General Procedure* ***G***, 4-benzylidene-2,6-di-*tert*-butylcyclohexa-2,5-dien-1-one **5** (74 mg, 0.25 mmol), 4'-nitrophenyl 2-(4''-methoxyphenyl)acetate **21** (108 mg, 0.375 mmol), (*S*)-TM·HCl (3 mg, 5 mol%) and Et_3N (35 µL, 0.25 mmol) were dissolved in anhydrous MeCN (0.42 mL). The reaction mixture was stirred at rt for 24 h before being concentrated *in vacuo*. The crude material (60:40 dr) was purified and the diastereoisomers separated by flash silica column chromatography (petroleum ether/EtOAc, 95:5). The diastereoisomers were obtained as pale yellow solids (combined: 144 mg, 99%).

mp 51-53 °C; **Chiral HPLC analysis**, Chiralpak AD-H (4.5% *i*-PrOH/hexane, flow rate 1.5 mLmin^{-1}, 211 nm, 40 °C), t_R major: 9.8 min and 18.6 min, 92:8 er, t_R minor: 13.1 min and 14.7 min, 87:13 er; **IR** ν_{max} (film)/cm^{-1} 3628 (O–H), 2953 (C–H), 1757 (C=O), 1524 (N-O), 1489 (C=C), 1344 (N-O), 1103 (C-O); **HRMS** (ASAP$^-$) $C_{36}H_{39}NO_6$ ([M]$^-$), found 581.2771, requires 581.2783 (−2.1 ppm).

Data for major diastereoisomer: $[\alpha]_D^{20}$ +85.0 (*c* 0.1, $CHCl_3$); **^{1}H NMR** (500 MHz, $CDCl_3$) δ_H: 1.30 (18H, s, (C(3'''')C(C*H*$_3$)$_3$, C(5'''')C(C*H*$_3$)$_3$), 3.70 (3H, s, OC*H*$_3$), 4.49 (1H, d, *J* 12.1, C(2)*H*), 4.59 (1H, d, *J* 12.1, C(1')*H*), 4.99 (1H, s, O*H*), 6.74 (2H, d, *J* 9.1, ester C(1)OC(2)*H*,

ester C(1)OC(6)*H*), 6.78 (2H, s, C(2'''')*H*, C(6'''')*H*), 6.83 (2H, d, *J* 8.7, C(3'')*H*, C(5'')*H*), 7.25 (2H, d, *J* 8.7, C(2'')*H*, C(6'')*H*), 7.32 (1H, d, *J* 7.4, C(4''')*H*), 7.41 (2H, t, *J* 7.4, C(3''')*H*, C(5''')*H*), 7.56 (2H, d, *J* 7.4, C(2''')*H*, C(6''')*H*), 8.14 (2H, d, *J* 9.1, ester C(3)*H*, ester C(5)*H*); **^{13}C{^{1}H} NMR** (126 MHz, $CDCl_3$) δ_C: 30.2 (C(3'''')C(CH_3)$_3$, C(5'''')C(CH_3)$_3$), 34.3 (C(3'''')*C*(CH_3)$_3$, C(5'''')*C*(CH_3)$_3$), 55.3 (OCH_3), 55.7 (*C*(1')), 57.2 (*C*(2)), 113.8 (*C*(3''), *C*(5'')), 122.6 (ester *C*(2), ester *C*(6)), 124.9 (ester *C*(3), ester *C*(5)), 125.1 (*C*(2''''), *C*(6'''')), 127.0 (*C*(4''')), 128.7 (*C*(2'''), *C*(6''')), 128.6 (*C*(3'''), *C*(5''')), 129.7 (*C*(2''), *C*(6'')), 131.0 (*C*(1''''), 133.3 (*C*(1'')), 135.3 (*C*(3''''), *C*(5'''')), 142.6 (*C*(1''')), 145.1 (ester C(1)O*C*(4)NO_2), 152.2 (*C*(4'''')), 155.2 (ester C(1)O*C*(1)), 159.1 (*C*(4'')), 170.9 (*C*(1)O).

Data for minor diastereoisomer: $[\alpha]_D^{20}$ +38.0 (*c* 0.1, $CHCl_3$); **^{1}H NMR** (500 MHz, $CDCl_3$) δ_H: 1.44 (18H, s, (C(3'''')C(C*H*$_3$)$_3$, C(5'''')C(C*H*$_3$)$_3$), 3.78 (3H, s, OC*H*$_3$), 4.62 (2H, s, C(2)*H*, C(1')*H*), 5.20 (1H, s, O*H*), 6.57 (2H, d, *J* 9.1, ester C(1)OC(2)*H*, ester C(1)OC(6)*H*), 6.81 (2H, d, *J* 8.8, C(2'')*H*, C(6'')*H*), 7.06-7.11 (1H, m, C(4''')*H*), 7.16-7.19 (2H, m, C(3''')*H*, C(5''')*H*), 7.18 (2H, s, C(2''')*H*, C(6''')*H*), 7.33 (s, C(2'''')*H*, C(6'''')*H*), 7.37 (2H, d, *J* 8.8, C(3'')*H*, C(5'')*H*), 8.11 (2H, d, *J* 9.1, ester C(3)*H*, ester C(5)*H*); **^{13}C{^{1}H} NMR** (126 MHz, $CDCl_3$) δ_C: 30.2 (C(3'''')C(CH_3)$_3$, C(5'''')C(CH_3)$_3$), 34.5 (C(3'''')*C*(CH_3)$_3$, C(5'''')*C*(CH_3)$_3$), 55.1 (OCH_3), 55.8 (*C*(1')), 57.0 (*C*(2)), 114.0 (*C*(2''), *C*(6'')), 122.6 (ester *C*(2), ester *C*(6)), 124.8 (*C*(2''''), *C*(6'''')), 125.0 (ester *C*(3), ester *C*(5)), 127.0 (*C*(4''')), 127.8 (*C*(1''''), 128.0 (*C*(3'''), *C*(5''')), 128.4 (*C*(2'''), *C*(6''')), 129.7 (*C*(3''), *C*(5'')), 132.7 (*C*(1'')), 136.0 (*C*(3''''), *C*(5'''')), 141.3 (*C*(1''')), 145.1 (ester C(1)O*C*(4)NO_2), 152.7 (*C*(4'''')), 155.4 (ester C(1)O*C*(1)), 158.9 (*C*(4'')), 171.1 (*C*(1)O).

(2*S*,1'*R*)-4-Nitrophenyl 1-(3'''',5''''-di-*tert*-butyl-4''''-hydroxyphenyl)-2-(4''-(trifluoromethyl)phenyl)-1'-phenylpropanoate (27)

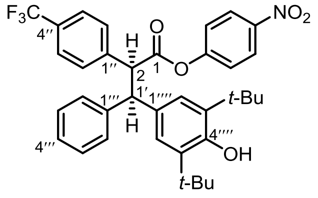

By following *General Procedure* ***G***, 4-benzylidene-2,6-di-*tert*-butylcyclohexa-2,5-dien-1-one **5** (74 mg, 0.25 mmol), 4'-nitrophenyl 2-(4''-(trifluoromethyl)phenyl)acetate **22** (122 mg, 0.375 mmol) and (*S*)-TM (2.6 mg, 5 mol%) were dissolved in anhydrous MeCN (0.42 mL). The reaction mixture was stirred at rt for 24 h before being concentrated *in vacuo*. The crude material (55:45 dr) was purified and the diastereoisomers separated by flash silica column

chromatography (petroleum ether/EtOAc, 95:5). The diastereoisomers were obtained as pale yellow solids (combined: 146 mg, 94%).

mp 91-93 °C; **Chiral HPLC analysis**,Chiralpak AD-H (0.8% *i*-PrOH/hexane, flow rate 1.5 mLmin^{-1}, 254 nm, 30 °C), t_R major: 28.4 min and 83.8 min, 88:12 er, t_R minor: 29.4 min and 42.8 min, 79:21 er ; **IR** ν_{max} (film)/cm^{-1} 3616 (O–H), 2955 (C–H), 1755 (C=O), 1522 (N-O), 1489 (C=C), 1323 (N-O), 1121 (C-O); **HRMS** (ASAP$^-$) $C_{36}H_{36}F_3NO_5$ ([M]), found 619.2540, requires 619.2551 (−1.8 ppm).

Data for major diastereoisomer (88:12 dr): $[\alpha]_D^{20}$ +50.0 (*c* 0.1, $CHCl_3$); **^{1}H NMR** (500 MHz, $CDCl_3$) δ_H: 1.25 (18H, s, (C(3'''')C(C*H*$_3$)$_3$, C(5'''')C(C*H*$_3$)$_3$), 4.57 (2H, s, C(2)*H*, C(1')*H*), 4.98 (1H, s, O*H*), 6.63-6.77 (4H, m, C(2'''')*H*, C(6'''')*H*, ester C(1)OC(2)*H*, ester C(1)OC(6)*H*), 7.29-7.35 (1H, m, C(4''')*H*), 7.38-7.44 (4H, m, *Ar*), 7.53 (4H, t, *J* 8.6, *Ar*), 8.13 (2H, d, *J* 9.1, ester C(3)*H*, ester C(5)*H*); **^{13}C{^{1}H} NMR** (126 MHz, $CDCl_3$) δ_C: 30.2 (C(3'''')C(*C*H$_3$)$_3$, C(5'''')C(*C*H$_3$)$_3$), 34.3 (C(3'''')*C*(CH$_3$)$_3$, C(5'''')*C*(CH$_3$)$_3$), 56.2 (*C*(1')), 58.0 (*C*(2)), 122.4 (ester *C*(2), ester *C*(6)), 124.9 (ester *C*(3), ester *C*(5)), 125.3 (*C*(2''''), *C*(6'''')), 125.5 (q, $^3J_{C\text{-}F}$ 3.1, C(3'',5'')), 127.4 (*C*(4''')), 128.2 (*Ar*), 129.0 (*Ar*), 129.2 (*Ar*), 130.5 (*C*(1'''')), 135.8 (*C*(3''''), *C*(5'''')), 140.6 (*C*(1'')), 142.0 (ester C(1)O*C*(4)NO$_2$), 145.6 (*C*(1''')), 152.6 (*C*(4'''')), 155.2 (ester C(1)O*C*(1)), 170.4 (*C*(1)O); **^{19}F NMR** (376 MHz, $CDCl_3$) δ_F: –62.7 (C*F*$_3$).

Data for minor diastereoisomer (79:21 dr): $[\alpha]_D^{20}$ +16.0 (*c* 0.1, $CHCl_3$); **^{1}H NMR** (500 MHz, $CDCl_3$) δ_H: 1.42 (18H, s, (C(3'''')C(C*H*$_3$)$_3$, C(5'''')C(C*H*$_3$)$_3$), 4.62 (1H, d, *J* 12.2, C(2)*H*) 4.73 (1H, d, *J* 12.2, C(1')*H*), 5.20 (1H, s, O*H*), 6.54 (2H, d, *J* 9.1, ester C(1)OC(2)*H*, ester C(1)OC(6)*H*), 7.06-7.12 (1H, m, C(4''')*H*), 7.13-7.19 (4H, m, C(2''')*H*, C(3''')*H*, C(5''')*H*, C(6''')*H*), 7.31 (2H, s, C(2'''')*H*, C(6'''')*H*), 7.48-7.54 (2H, m, *J* 8.2, C(2'')*H*, C(6'')*H*), 7.57 (2H, d, *J* 8.3, C(3'')*H*, C(5'')*H*), 8.09 (2H, d, *J* 9.1, ester C(3)*H*, ester C(5)*H*); **^{13}C{^{1}H} NMR** (126 MHz, $CDCl_3$) δ_C: 30.4 (C(3'''')C(*C*H$_3$)$_3$, C(5'''')C(*C*H$_3$)$_3$), 34.6 (C(3'''')*C*(CH$_3$)$_3$, C(5'''')*C*(CH$_3$)$_3$), 56.2 (*C*(1')), 57.7 (*C*(2)), 122.4 (ester *C*(2), ester *C*(6)), 124.9 (*C*(2''''), *C*(6'''')), 125.2 (ester *C*(3), ester *C*(5)), 125.7 (q, $^3J_{C\text{-}F}$ 3.9, *C*(3''), *C*(5'')), 126.9 (*Ar*), 128.0 (*Ar*), 128.7 (*Ar*), 129.3 (*C*(2''), *C*(6'')), 132.2 (*C*(1'''')), 136.4 (*C*(3''''), *C*(5'''')), 140.1 (*C*(1'')), 140.9 (ester C(1)O*C*(4)NO$_2$), 145.6 (*C*(1''')), 153.1 (*C*(4'''')), 155.3 (ester C(1)O*C*(1)), 170.5 (*C*(1)O); **^{19}F NMR** (376 MHz, $CDCl_3$) δ_F: –62.6 (C*F*$_3$).

(2*S*,1′*R*)-4-Nitrophenyl 1-(3′′′′,5′′′′-di-*tert*-butyl-4′′′′-hydroxyphenyl)-2-(naphthalen-2′′-yl)-1′-phenylpropanoate (28)

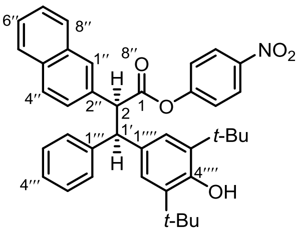

By following *General Procedure* ***G***, 4-benzylidene-2,6-di-*tert*-butylcyclohexa-2,5-dien-1-one **5** (74 mg, 0.25 mmol), 4'-nitrophenyl 2-(naphthalen-2"-yl)acetate **23** (115 mg, 0.375 mmol) and (*S*)-TM (2.6 mg, 5 mol%) were dissolved in anhydrous MeCN (0.42 mL). The reaction mixture was stirred at rt for 24 h before being concentrated *in vacuo*. The crude material (55:45 dr) was purified and the diastereoisomers separated by flash silica column chromatography (petroleum ether/EtOAc, 95:5). The diastereoisomers were obtained as pale yellow solids (combined: 149 mg, 99%).

mp 48-50 °C; **Chiral HPLC analysis**, Chiralpak AD-H (1.5% *i*-PrOH/hexane, flow rate 1.5 mLmin^{-1}, 211 nm, 30 °C), t_R major: 27.9 min and 53.4 min, 91:9 er, t_R minor: 22.6 min and 36.7 min, 85:15 er; **IR** ν_{max} (film)/cm^{-1} 3628 (O–H), 2958 (C–H), 1757 (C=O), 1524 (N-O), 1489 (C=C), 1344 (N-O), 1103 (C-O); **HRMS** (ASAP^{-}) $C_{39}H_{39}NO_5$ ([M]$^{-}$), found 601.2831, requires 601.2835 (−0.7 ppm).

Data for major diastereoisomer: $[\alpha]_D^{20}$ +93.0 (*c* 0.1, $CHCl_3$); **^{1}H NMR** (500 MHz, $CDCl_3$) δ_H: 1.15 (18H, s, (C(3'''')C(C*H*$_3$)$_3$, C(5'''')C(C*H*$_3$)$_3$), 4.71 (2H, d, *J* 2.2, C(2)*H*), C(1')*H*), 4.87 (1H, s, O*H*), 6.74 (2H, d, *J* 9.2, ester C(1)OC(2)*H*, ester C(1)OC(6)*H*), 6.75 (2H, s, C(2'''')*H*, C(6'''')*H*), 7.31 (1H, t, *J* 7.4, C(4''')*H*), 7.42 (2H, d, *J* 7.7, *Ar*), 7.44-7.47 (2H, m, C(3''')*H*, C(5''')*H*), 7.50 (1H, dd, *J* 8.5, 1.8, *Ar*), 7.59 (2H, d, *J* 7.2, C(2''')*H*, C(6''')*H*), 7.69 (1H, s, *Ar*), 7.72 (1H, dd, *J* 6.0, 3.4, *Ar*), 7.76 (1H, d, *J* 8.5, *Ar*), 7.79 (1H, dd, *J* 6.1, 3.4, *Ar*), 8.12 (2H, d, *J* 9.1, ester C(3)*H*, ester C(5)*H*); **^{13}C{^{1}H} NMR** (126 MHz, $CDCl_3$) δ_C: 30.1 (C(3'''')C(*C*H$_3$)$_3$, C(5'''')C(*C*H$_3$)$_3$), 34.2 (C(3'''')*C*(CH$_3$)$_3$, C(5'''')*C*(CH$_3$)$_3$), 55.8 (*C*(1')), 58.2 (*C*(2)), 122.5 (ester *C*(2), ester *C*(6)), 125.0 (ester *C*(3), ester *C*(5)), 125.2 (*C*(2''''), *C*(6'''')), 126.2 (*Ar*), 126.3 (*Ar*), 126.4 (*Ar*), 127.1 (*C*(4''')), 127.6 (*Ar*), 127.9 (*Ar*), 128.3 (*C*(2'''), *C*(6''')), 128.4 (*Ar*), 128.4 (*Ar*), 128.9 (*C*(3'''), *C*(5''')), 131.1 (*C*(4''a), 132.9 (*C*(8''a)), 133.4 (*C*(1''''), 133.8 (*C*(2'')), 135.5 (*C*(3''''), *C*(5'''')), 142.6 (*C*(3''')), 145.5 (ester C(1)O*C*(4)NO$_2$), 152.3 (*C*(4'''')), 155.4 (ester C(1)O*C*(1)), 171.0 (*C*(1)O).

Data for minor diastereoisomer: $[\alpha]_D^{20}$ +38.0 (*c* 0.1, $CHCl_3$); **^{1}H NMR** (500 MHz, $CDCl_3$) δ_H: 1.46 (18H, s, (C(3'''')C(C*H*$_3$)$_3$, C(5'''')C(C*H*$_3$)$_3$), 4.80 (1H, d, *J* 12.2, C(2)*H*), 4.87 (1H, d, *J* 12.2, C(1')*H*), 5.22 (1H, s, O*H*), 6.57 (2H, d, *J* 9.1, ester C(1)OC(2)*H*, ester C(1)OC(6)*H*), 7.03 (1H, t, *J* 7.4, C(4''')*H*), 7.12 (2H, t, *J* 7.4, C(3''')*H*, C(5''')*H*), 7.22 (2H, t, *J* 7.4, C(2''')*H*, C(6''')*H*), 7.38 (2H, s, C(2'''')*H*, C(6'''')*H*), 7.48 (2H, t, *J* 4.2, *Ar*), 7.52 (1H, dd, *J* 8.5, 1.7, *Ar*), 7.77-7.82 (3H, m, *Ar*), 7.92 (1H, s, *Ar*), 8.10 (2H, d, *J* 9.1, ester C(3)*H*, ester C(5)*H*); **^{13}C{^{1}H} NMR** (126 MHz, $CDCl_3$) δ_C: 30.4 (C(3'''')C(*C*H$_3$)$_3$, C(5'''')C(*C*H$_3$)$_3$), 34.3 (C(3'''')*C*(CH$_3$)$_3$, C(5'''')*C*(CH$_3$)$_3$), 51.2 (*C*(1')), 54.3 (*C*(2)), 122.4 (ester *C*(2), ester *C*(6)), 124.7 (*C*(2''''), *C*(6'''')), 125.0 (ester *C*(3), ester *C*(5)), 126.2 (*Ar*), 126.3 (*Ar*), 126.5 (*C*(4''')), 127.6 (*Ar*), 127.8 (*Ar*), 127.9 (*C*(2'''), *C*(6''')), 128.2 (*Ar*), 128.4(*Ar*), 128.5(*C*(3'''), *C*(5''')), 132.7 (*Ar*), 132.8 (*Ar*), 133.3 (*Ar*), 133.4 (*Ar*), 136.0 (*C*(3''''), *C*(5'''')), 141.3 (*C*(3''')), 145.3 (ester C(1)O*C*(4)NO$_2$), 152.7 (*C*(4'''')), 155.4 (ester C(1)O*C*(1)), 170.9 (*C*(1)O).

(2*S*,1′*R*)-4-Nitrophenyl 1-(3′′′′,5′′′′-di-*tert*-butyl-4′′′′-hydroxyphenyl)-2-(naphthalen-1′′-yl)-1′-phenylpropanoate (29)

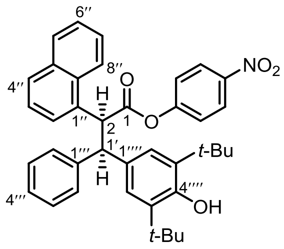

By following *General Procedure* ***G***, 4-benzylidene-2,6-di-*tert*-butylcyclohexa-2,5-dien-1-one **5** (74 mg, 0.25 mmol), 4'-nitrophenyl 2-(naphthalen-1''-yl)acetate **24** (115 mg, 0.375 mmol), (*S*)-TM·HCl (3 mg, 5 mol%) and Et_3N (35 µL, 0.25 mmol) were dissolved in anhydrous MeCN (0.42 mL). The reaction mixture was stirred at rt for 24 h before being concentrated *in vacuo*. The crude material (60:40 dr) was purified and the diastereoisomers separated by flash silica column chromatography (petroleum ether/EtOAc, 95:5). The diastereoisomers were obtained as pale yellow solids (combined: 149 mg, 99%).

mp 54-56 °C; **Chiral HPLC analysis**, Chiralcel OD-H (2% *i*-PrOH/hexane, flow rate 1.5 mLmin^{-1}, 211 nm, 30 °C), t_R major: 9.8 min and 21.2 min, 93:7 er, t_R minor: 6.1 min and 28.2 min, 94:6 er; **IR** ν_{max} (film)/cm^{-1} 3620 (O–H), 2957 (C–H), 1757 (C=O), 1524 (N-O), 1489 (C=C), 1344 (N-O), 1105 (C-O); **HRMS** (ASAP$^-$) $C_{39}H_{39}NO_5$ ([M]$^-$), found 601.2828, requires 601.2835 (−1.2 ppm).

Data for major diastereoisomer: $[\alpha]_D^{20}$ +124 (*c* 0.1, $CHCl_3$); **^{1}H NMR** (500 MHz, $CDCl_3$) δ_H: 1.12 (18H, s, (C(3'''')C(C*H*$_3$)$_3$, C(5'''')C(C*H*$_3$)$_3$), 4.80 (1H, s, O*H*), 4.86 (1H, d, *J* 11.7, C(2)*H*), 5.54 (1H, d, *J* 11.7, C(1')*H*), 6.70 (2H, s, C(2'''')*H*, C(6'''')*H*), 6.79 (2H, d, *J* 9.1, ester C(1)OC(2)*H*, ester C(1)OC(6)*H*), 7.37 (1H, t, *J* 7.4, C(4''')*H*), 7.42 (2H, q, *J* 8.0, 7.4, *Ar*), 7.48 (2H, t, *J* 7.4, C(3''')*H*, C(5''')*H*), 7.55 (2H, t, *J* 7.6, *Ar*), 7.69 (2H, d, *J* 7.4, C(2''')*H*, C(6''')*H*), 7.78-7.84 (2H, m, *Ar*), 7.94-8.03 (2H, m, C(5'')*H*, C(8'')*H*), 8.14 (2H, d, *J* 9.1, ester C(3)*H*, ester C(5)*H*); **^{13}C{^{1}H} NMR** (126 MHz, $CDCl_3$) δ_C: 30.6 (C(3'''')C(*C*H$_3$)$_3$, C(5'''')C(*C*H$_3$)$_3$), 33.9 (C(3'''')*C*(CH$_3$)$_3$, C(5'''')*C*(CH$_3$)$_3$), 50.8 (*C*(1')), 55.9 (*C*(2)), 122.2 (ester *C*(2), ester *C*(6)), 124.8 (*Ar*), 125.0 (ester *C*(3), ester *C*(5)), 125.2 (*C*(2''''), *C*(6'''')), 125.4 (*Ar*), 125.5 (*Ar*), 126.2 (*Ar*), 127.1 (*C*(4''')), 128.2 (*C*(2'''), *C*(6''')), 128.4 (*Ar*), 128.7 (*Ar*), 128.9 (*C*(3'''), *C*(5''')), 130.6 (*C*(8''a), 132.1 (*C*(4''a)), 132.9 (*C*(1''''), 133.6 (*C*(1'')), 134.8 (*C*(3''''), *C*(5'''')), 142.6 (*C*(3''')), 145.1 (ester C(1)O*C*(4)NO$_2$), 155.9 (*C*(4'''')), 155.2 (ester C(1)O*C*(1)), 171.1 (*C*(1)O).

Data for minor diastereoisomer: $[\alpha]_D^{20}$ +33.0 (*c* 0.1, $CHCl_3$); **^{1}H NMR** (500 MHz, $CDCl_3$) δ_H: 1.48 (18H, s, (C(3'''')C(C*H*$_3$)$_3$, C(5'''')C(C*H*$_3$)$_3$), 5.04 (1H, d, *J* 12.1, C(2)*H*), 5.24 (1H, s, O*H*), 5.66 (1H, d, *J* 12.1, C(1')*H*), 6.54 (2H, d, *J* 8.6, ester C(1)OC(2)*H*, ester C(1)OC(6)*H*), 6.98 (1H, t, *J* 7.5, *Ar*), 7.06 (2H, t, *J* 7.5, *Ar*), 7.21 (2H, d, *J* 7.5, *Ar*), 7.41 (2H, s, C(2'''')*H*, C(6'''')*H*), 7.47 (1H, t, *J* 7.5, *Ar*), 7.52 (1H, t, *J* 7.5, *Ar*), 7.63 (1H, t, *J* 7.5, *Ar*), 7.77 (1H, d, *J* 8.2, *Ar*), 7.87 (1H, d, *J* 8.2, *Ar*), 7.91 (1H, d, *J* 7.5, *Ar*), 8.08 (2H, d, *J* 8.6, ester C(3)*H*, ester C(5)*H*), 8.42 (1H, d, *J* 8.6, *Ar*); **^{13}C{^{1}H} NMR** (126 MHz, $CDCl_3$) δ_C: 30.4 (C(3'''')C(*C*H$_3$)$_3$, C(5'''')C(*C*H$_3$)$_3$), 34.3 (C(3'''')*C*(CH$_3$)$_3$, C(5'''')*C*(CH$_3$)$_3$), 51.2 (*C*(1')), 54.3 (*C*(2)), 122.4 (ester *C*(2), ester *C*(6)), 122.6 (*Ar*), 124.9 (ester *C*(3), ester *C*(5)), 125.1 (*C*(2''''), *C*(6'''')), 125.5 (*Ar*), 125.6 (*Ar*), 126.3 (*Ar*), 126.5 (*Ar*), 127.7 (*Ar*), 128.3 (*Ar*), 129.1 (*Ar*), 131.7 (*C*(8''a), 132.1 (*C*(4''a)), 132.8 (*C*(1''''), 133.8 (*C*(1'')), 136.1 (*C*(3''''), *C*(5'''')), 141.3 (*C*(3''')), 145.3 (ester C(1)O*C*(4)NO$_2$), 152.8 (*C*(4'''')), 155.3 (ester C(1)O*C*(1)), 170.9 (*C*(1)O).

(2*S*,1′*R*)-4-Nitrophenyl 1-(3′′′′,5′′′′-di-*tert*-butyl-4′′′′-hydroxyphenyl)-2-(thiophen-3′′-yl)-1′-phenylpropanoate (30)

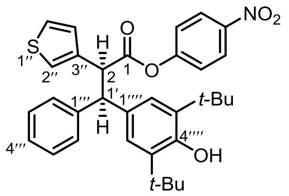

By following *General Procedure* ***G***, 4-benzylidene-2,6-di-*tert*-butylcyclohexa-2,5-dien-1-one **5** (74 mg, 0.25 mmol), 4'-nitrophenyl 2-(thiophen-3"-yl)acetate **25** (99 mg, 0.375 mmol), (*S*)-TM·HCl (3 mg, 5 mol%) and Et_3N (35 µL, 0.25 mmol) were dissolved in anhydrous MeCN (0.42 mL). The reaction mixture was stirred at rt for 24 h before being concentrated *in vacuo*. The crude material (55:45 dr) was purified and the diastereoisomers separated by flash silica column chromatography (petroleum ether/EtOAc, 95:5). The diastereoisomers were obtained as pale yellow solids (combined: 131 mg, 94%).

mp 54-56 °C; **Chiral HPLC analysis**, Chiralpak AD-H (1.5% *i*-PrOH/hexane, flow rate 1.5 $mLmin^{-1}$, 211 nm, 30 °C), t_R major: 19.6 min and 28.7 min, 88:12 er, t_R minor: 22.6 min and 24.0 min, 90:10 er; **IR** ν_{max} (film)/cm^{-1} 3612 (O–H), 2955 (C–H), 1759 (C=O), 1524 (N-O), 1489 (C=C), 1344 (N-O), 1107 (C-O); **HRMS** ($ASAP^-$) $C_{33}H_{35}NO_5S$ ($[M]^-$), found 557.2226, requires 557.2241 (−2.7 ppm).

Data for major diastereoisomer: $[\alpha]_D^{20}$ +54.0 (*c* 0.1, $CHCl_3$); **^{1}H NMR** (400 MHz, $CDCl_3$) δ_H: 1.33 (18H, s, (C(3"")C(C*H*3)3, C(5"")C(C*H*3)3), 4.57 (1H, d, *J* 12.0, C(2)*H*), 4.67 (1H, d, *J* 12.0, C(1')*H*), 5.02 (1H, s, O*H*), 6.72 (2H, d, *J* 9.1, ester C(1)OC(2)*H*, ester C(1)OC(6)*H*), 6.82 (2H, s, C(2"")*H*, C(6"")*H*), 7.02 (2H, dd, *J* 5.0, 1.2, C(4")*H*), 7.08 (2H, dd, *J* 2.9, 1.2, C(2")*H*), 7.23-7.27 (1H, m, C(5")*H*), 7.32 (1H, d, *J* 7.4, C(4‴)*H*), 7.41 (2H, t, *J* 7.4, C(3‴)*H*, C(5‴)*H*), 7.55 (2H, d, *J* 7.4, C(2‴)*H*, C(6‴)*H*), 8.15 (2H, d, *J* 9.1, ester C(3)*H*, ester C(5)*H*); **^{13}C{^{1}H} NMR** (101 MHz, $CDCl_3$) δ_C: 30.3 (C(3"")C(*C*H3)3, C(5"")C(*C*H3)3), 34.0 (C(3"")*C*(CH3)3, C(5"")*C*(CH3)3), 53.7 (*C*(1')), 55.8 (*C*(2)), 122.3 (ester *C*(2), ester *C*(6)), 123.4 (*C*(2")), 124.8 (*C*(2""), *C*(6"")), 125.2 (ester *C*(3), ester *C*(5)), 125.6 (*C*(5")), 127.0 (*C*(4‴)), 127.5 (*C*(4")), 128.2 (*C*(2‴), *C*(6‴)), 128.7 (*C*(3‴), *C*(5‴)), 131.1 (*C*(3"), 135.4 (*C*(3""), *C*(5"")), 136.5 (ester C(1)O*C*(4)NO_2), 142.2 (*C*(3"")), 145.2 (*C*(1"")), 152.2 (*C*(4"")), 155.0 (ester C(1)O*C*(1)), 170.3 (*C*(1)O).

Data for minor diastereoisomer: $[\alpha]_D^{20}$ +35.0 (*c* 0.1, $CHCl_3$); **^{1}H NMR** (400 MHz, $CDCl_3$) δ_H: 1.44 (18H, s, (C(3"")C(C*H*3)3, C(5"")C(C*H*3)3), 4.56 (1H, d, *J* 12.1, C(2)*H*), 4.81 (1H, d, *J* 12.1, C(1')*H*), 5.20 (1H, s, O*H*), 6.56 (2H, d, *J* 9.2, ester C(1)OC(2)*H*, ester C(1)OC(6)*H*), 7.11-7.16 (2H, m, *Ar*), 7.17 -7.22 (5H, m, *Ar*), 7.24 (1H, dd, *J* 5.0, 3.0, *Ar*), 7.34 (2H, s,

C(2'''')*H*, C(6'''')*H*), 8.11 (2H, d, *J* 9.2, ester C(3)*H*, ester C(5)*H*); **^{13}C{^{1}H} NMR** (101 MHz, $CDCl_3$) δ_C: 30.1 (C(3'''')C(CH$_3$)$_3$, C(5'''')C(CH$_3$)$_3$), 34.3 (C(3'''')C(CH$_3$)$_3$, C(5'''')C(CH$_3$)$_3$), 53.3 (*C*(1')), 56.1 (*C*(2)), 122.5 (ester *C*(2), ester *C*(6)), 123.6 (*Ar*), 124.8 (*C*(2''''), *C*(6'''')), 125.0 (ester *C*(3), ester *C*(5)), 125.8 (*Ar*), 126.5 (*Ar*), 127.3 (*Ar*), 127.9 (*Ar*), 128.4 (*Ar*), 132.3 (*C*(3''), 136.1 (*C*(3''''), *C*(5'''')), 136.2 (ester C(1)O*C*(4)NO_2), 141.3 (*C*(3''')), 145.2 (*C*(1'''')), 152.7 (*C*(4'''')), 155.2 (ester C(1)O*C*(1)), 170.5 (*C*(1)O).

Additional Reaction Optimization Tables

Solvent Screen:

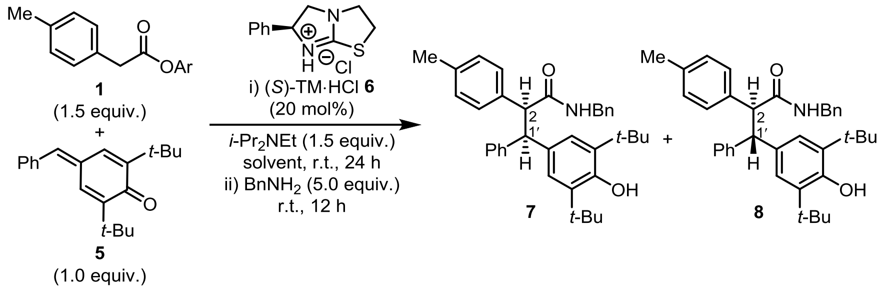

Entry	Solvent	Yield (%)	dr (7:8)	er (7)	er (8)
1	PhMe	0	-	-	-
2	CH_2Cl_2	0	-	-	-
3	THF	0	-	-	-
4	$CHCl_3$	0	-	-	-
5	1,4-Dioxane	0	-	-	-
6	MeCN	82	53:47	97:3	94:6
7	DMF	93	50:50	93:7	85:15

Table S1. Solvent screen. Isolated yield is a mixture of diastereoisomers **7** and **8**; dr determined by ^{1}H NMR spectroscopic analysis of the crude reaction mixture; er determined by chiral stationary phase (CSP) HPLC analysis: **7** (2*S*,1′*R*:2*R*,1′*S*) and **8** (2*S*,1′*S*:2*R*,1′*R*).

Solvent/Catalyst Screen:

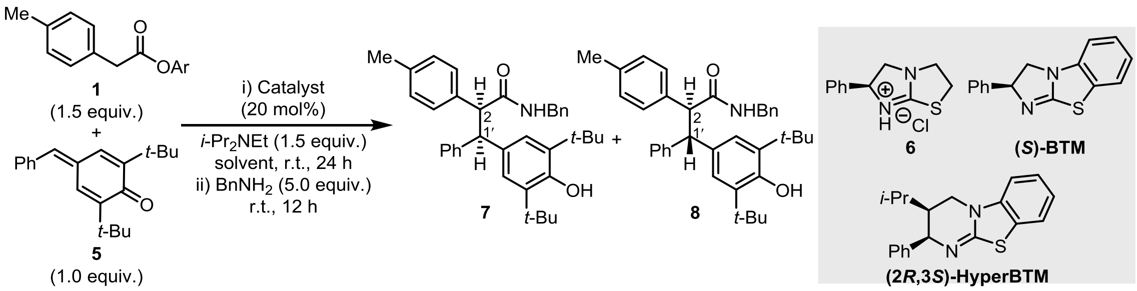

Entry	Catalyst	Solvent	Yield (%)	dr (7:8)	er (7)	er (8)
1	-	MeCN	0	-	-	-
2	-	DMF	84	65:35	50:50	50:50
3	**6**	MeCN	82	55:45	97:3	94:6
4	**6**	DMF	93	50:50	93:7	85:15
5	**BTM**	MeCN	64	60:40	98:2	93:7
6	**BTM**	DMF	98	55:45	90:10	77:23
7	**HyperBTM**	MeCN	4	55:45	70:30	69:31
8	**HyperBTM**	DMF	62	60:40	68:32	62:38

Table S2. Solvent/Catalyst screen. Isolated yield is a mixture of diastereoisomers **7** and **8**; dr determined by ^{1}H NMR spectroscopic analysis of the crude reaction mixture; er determined by chiral stationary phase (CSP) HPLC analysis: **7** (2*S*,1′*R*:2*R*,1′*S*) and **8** (2*S*,1′*S*:2*R*,1′*R*).

Base Screen:

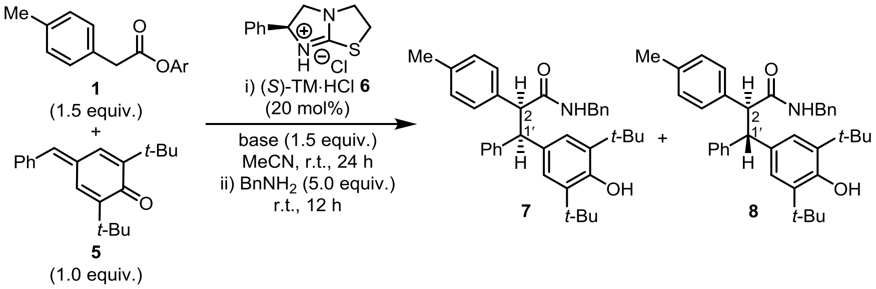

Entry	Base	Yield (%)	dr (7:8)	er (7)	er (8)
1	*i*-Pr_2NEt	82	55:45	97:3	94:6
2	*i*-Pr_2NH	16	55:45	96:4	94:6
3	Et_3N	98	60:40	97:3	94:6
4	DBU	70	60:40	76:24	81:19
5	DABCO	99	60:40	80:20	71:29
6	K_3PO_4	71	55:45	86:14	83:17
7	Cs_2CO_3	83	55:45	80:20	85:15
8	Na_2CO_3	87	60:40	97:3	96:4
9	K_2CO_3	99	60:40	96:4	91:9

Table S3. Base screen. Isolated yield is a mixture of diastereoisomers **7** and **8**; dr determined by ^{1}H NMR spectroscopic analysis of the crude reaction mixture; er determined by chiral stationary phase (CSP) HPLC analysis: **7** (2*S*,1′*R*:2*R*,1′*S*) and **8** (2*S*,1′*S*:2*R*,1′*R*).

Control Reactions

(a) PNP ester product (19 and 20) epimerization studies (Scheme 2)

A control reaction was conducted individually on diastereoisomers **19** and **20** to check for any change in dr (*via* epimerization) under the catalysis conditions. The diastereoisomers **19** and **20** were added to separate NMR tubes and dissolved in CD_3CN (0.18 M). At room temperature, *i*-Pr_2NEt (1.5 equiv.) was added to each sample. After 5 h, there was no change in ^1H NMR of either diastereoisomer. Therefore, **6** (20 mol%) was added and the mixture left for a further 15 h. Again, there was no evidence of epimerization. To mimic the aryloxide expected to be present under reaction conditions, tetrabutylammonium 4-nitrophenoxide (0.2 equiv.) was added. After a further 7 h, still no change in dr was observed. Addition of $BnNH_2$ (5.0 equiv.) formed the corresponding amide products **7** and **8** (from PNP esters **19** and **20**), each as a single diastereoisomer. Overall, sequential addition of the reagents under reaction conditions did not lead to formation of the opposite diastereoisomer in either case. This is consistent with no epimerization of the diastereoisomers **19** and **20** under the reaction conditions, and therefore the crude dr is representative of the kinetic dr of the reaction.

(b) Reagent compatibility

A series of control reactions were conducted to invesitgate reagent compatibility. Firstly, *p*-QM **5** (1.0 equiv.) was mixed with free base tetramisole **6** (1.0 equiv.) and stirred in CD_3CN at room temperature for 24 h. Only *p*-QM **5** and tetramisole **6** were observed, with no indication of any new species by ^1H NMR spectroscopic analysis. Next, *p*-QM **5** (1.0 equiv.) was mixed with tetrabutylammonium 4-nitrophenoxide (1.0 equiv.) and stirred in CD_3CN at room temperature for 24 h. ^1H NMR spectroscopy revealed only *p*-QM **5** and tetrabutylammonium 4-nitrophenoxide were present suggesting no side reactions between the *p*-QM **5** and the phenoxide counterion were taking place. These control reactions help explain the success of the aryloxide-promoted catalyst turnover concept since the reagents involved are compatible.

X-Ray Crystal Structure Information for 19

X-ray diffraction data were collected at 173 K using a Rigaku MM-007HF High Brilliance RA generator/confocal optics with XtaLAB P100 diffractometer [Cu Kα radiation (λ = 1.54187 Å)]. Data were collected using CrystalClear[6] and processed (including correction for Lorentz, polarization and absorption) using CrysAlisPro.[7] Structures were solved by dual-space (SHELXT[8]), direct (SIR2011[9]) or charge-flipping (Superflip[10]) methods and refined by full-matrix least-squares against F^2 (SHELXL-2018/3[11]). Non-hydrogen atoms were refined anisotropically, and all hydrogen atoms were refined using a riding model. All calculations were performed using the CrystalStructure[12] interface.

	**(2*S*,1′*R*)-19**
CCDC	1992504
empirical formula	$C_{36}H_{39}NO_5$
fw	565.71
crystal description	colourless prism
crystal size [mm]	0.10×0.10×0.10
space group	*P*1 (#1)
a [Å]	11.8586(2)
b [Å]	11.9458(3)
c [Å]	12.2838(3)
vol [Å]^3	1562.80(6)
α [°]	75.9529(19)
β [°]	72.0808(19)
γ [°]	73.2733(18)
Z	2
ρ (calc) [g/cm^3]	1.202
μ [mm^{-1}]	0.635
F(000)	604
reflections collected	15725
independent reflections (R_{int})	7160 (0.0270)
data/parameters	7160/760
GOF on F^2	1.133
R_1 [$I > 2\sigma(I)$]	0.0542
wR_2 (all data)	0.1210
largest diff. peak/hole [e/Å^3]	0.45, −0.52
Flack parameter	−0.04(18)

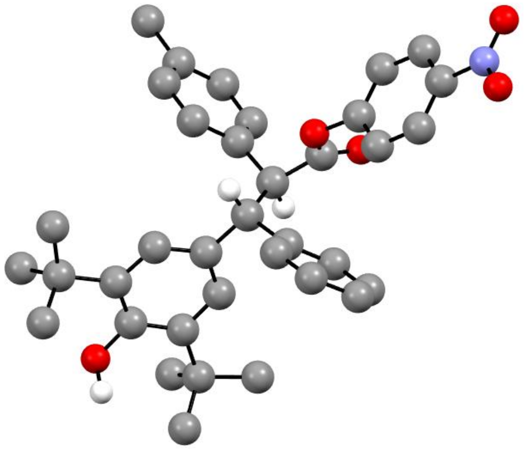

References

[1] L. Roiser, M. Waser, *Org. Lett.* **2017**, *19*, 2338-2341.

[2] D. Richter, N. Hampel, T. Singer, A. R. Ofial, H. Mayr, *Eur. J. Org. Chem.* **2009**, 3203-3211.

[3] B. S. Donslund, T. K. Johansen, P. H. Poulsen, K. S. Halskov, K. A. Jørgensen, *Angew. Chem., Int. Ed.* **2015**, *54*, 13860-13874.

[4] J. N. Arokianathar, A. B. Frost, A. M. Z. Slawin, D. Stead, A. D. Smith, *ACS Catal.* **2018**, *8*, 1067-1075.

[5] K. J. Schwarz, J. L. Amos, J. C. Klein, D. T. Do, T. N. Snaddon, *J. Am. Chem. Soc.* **2016**, *138*, 5214–5217

[6] CrystalClear-SM Expert v2.1. Rigaku Americas, The Woodlands, Texas, USA, and Rigaku Corporation, Tokyo, Japan, 2015.

[7] CrysAlisPro v1.171.38.46. Rigaku Oxford Diffraction, Rigaku Corporation, Oxford, U.K. 2015.

[8] G. M. Sheldrick, *Acta Crystallogr., Sect. A.* **2015**, *71*, 3–8.

[9] M. C. Burla, R. Caliandro, M. Camalli, B. Carrozzini, G. L. Cascarano, C. Giacovazzo, M. Mallamo, A. Mazzone, G. Polidori, R. Spagna, *J. Appl. Cryst.* **2012**, *45*, 357–361.

[10] Palatinus, L. Chapuis, G. *J. Appl. Cryst.* **2007**, *40*, 786–790.

[11] G. M. Sheldrick, *Acta Crystallogr., Sect. C.* **2015**, *71*, 3–8.

[12] CrystalStructure v4.3.0. Rigaku Americas, The Woodlands, Texas, USA, and Rigaku Corporation, Tokyo, Japan, 2018.

HPLC Traces

HPLC data for **7 & 8** (Table 1, entry 8): Chiralpak AD-H (10% *i*-PrOH/hexane, flow rate 1.5 mLmin^{-1}, 211 nm, 40 °C), t_R major: 8.5 min and 29.3 min, 92:8 er, t_R minor: 3.8 min and 18.6 min, 85:15 er.

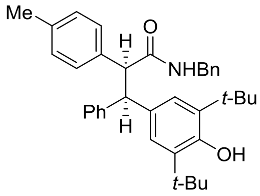

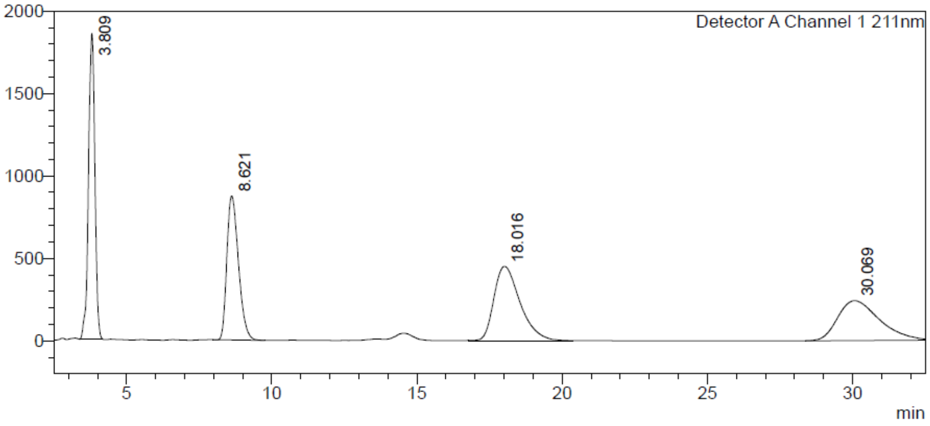

Detector A Channel 1 211nm

Peak#	Ret. Time	Area%
1	3.809	26.980
2	8.621	23.131
3	18.016	26.745
4	30.069	23.145
Total		100.000

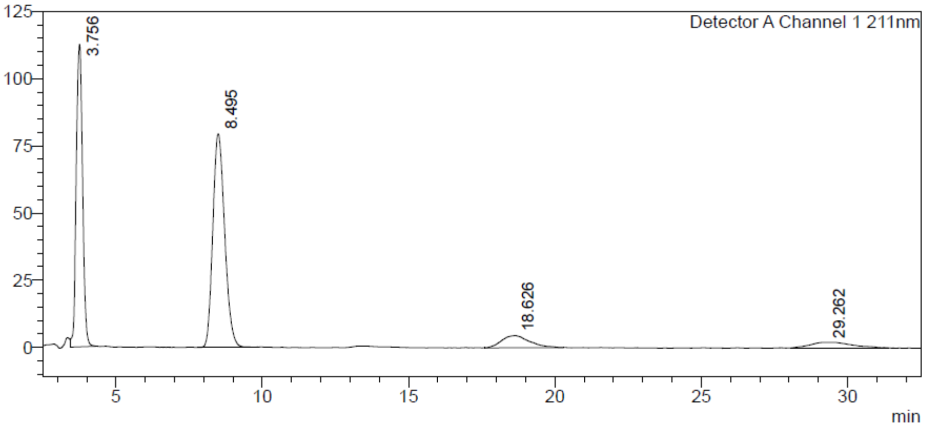

Detector A Channel 1 211nm

Peak#	Ret. Time	Area%
1	3.756	38.227
2	8.495	50.654
3	18.626	6.695
4	29.262	4.424
Total		100.000

HPLC data for **17**: Chiralpak AD-H (5% *i*-PrOH/hexane, flow rate 1.5 mLmin^{-1}, 211 nm, 40 °C), t_R major: 5.9 min and 11.8 min, 89:11 er, t_R minor: 4.8 min and 14.2 min, 81:19 er.

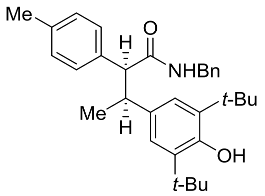

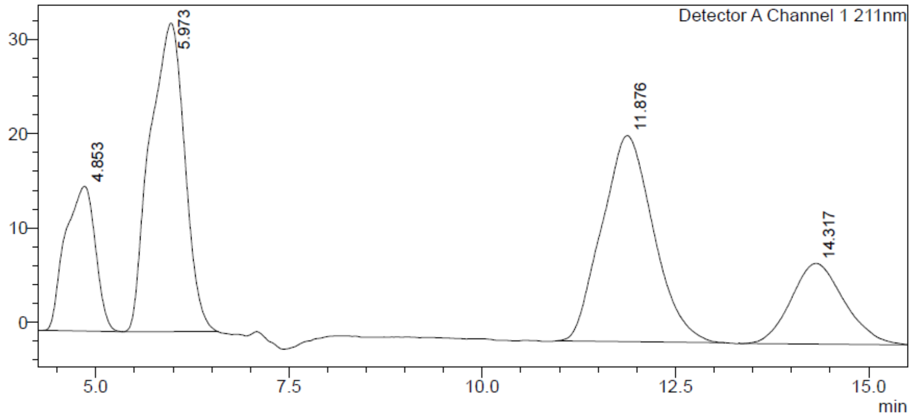

Detector A Channel 1 211nm

Peak#	Ret. Time	Area%
1	4.853	14.503
2	5.973	35.682
3	11.876	35.482
4	14.317	14.333
Total		100.000

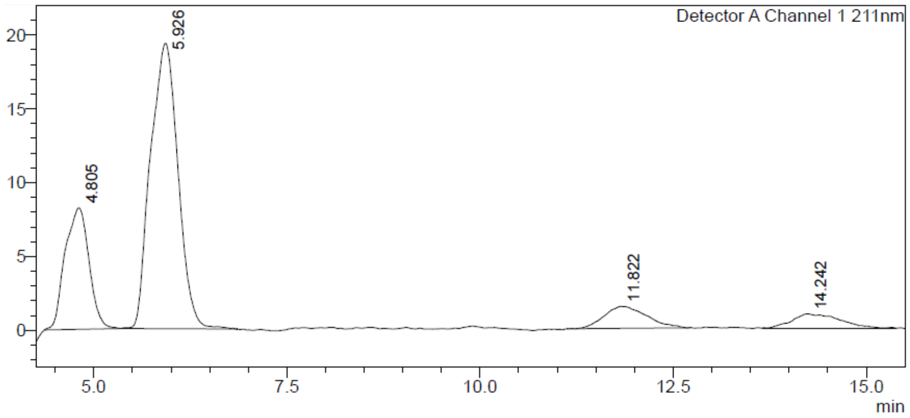

Detector A Channel 1 211nm

Peak#	Ret. Time	Area%
1	4.805	23.542
2	5.926	63.507
3	11.822	7.570
4	14.242	5.381
Total		100.000

HPLC data for **18**: Chiralpak AD-H (10% *i*-PrOH/hexane, flow rate 1.5 mLmin^{-1}, 211 nm, 40 °C), t_R major: 11.9 min and 17.6 min, 94:6 er, t_R minor: 2.2 min and 5.1 min, 63:37 er.

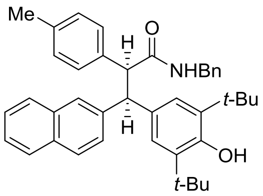

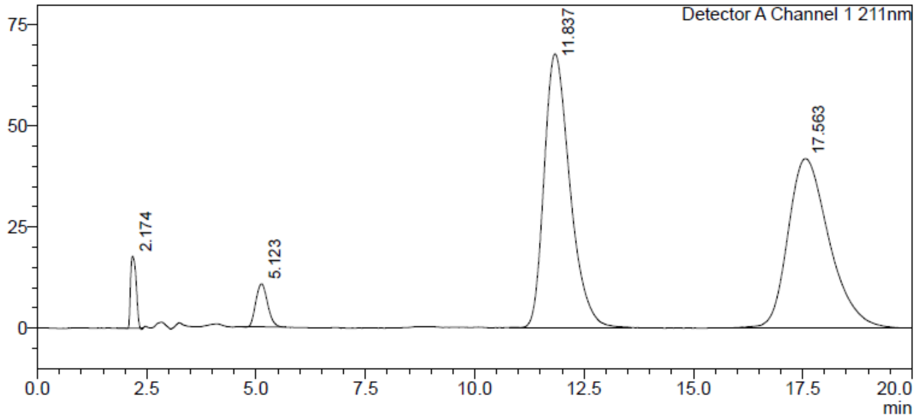

Detector A Channel 1 211nm

Peak#	Ret. Time	Area%
1	2.174	2.674
2	5.123	3.376
3	11.837	47.312
4	17.563	46.638
Total		100.000

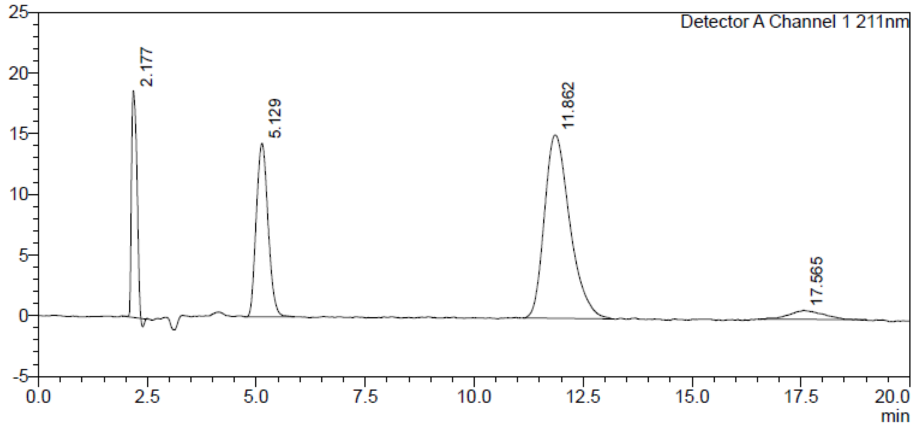

Detector A Channel 1 211nm

Peak#	Ret. Time	Area%
1	2.177	14.720
2	5.129	24.928
3	11.862	56.717
4	17.565	3.635
Total		100.000

HPLC data for **19 & 20**: Chiralpak AD-H (2% *i*-PrOH/hexane, flow rate 1.5 mLmin^{-1}, 211 nm, 30 °C), t_R major: 12.9 min and 23.6 min, 92:8 er, t_R minor: 20.5 min and 35.1 min, 87:13 er.

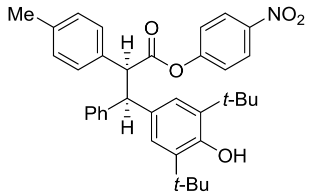

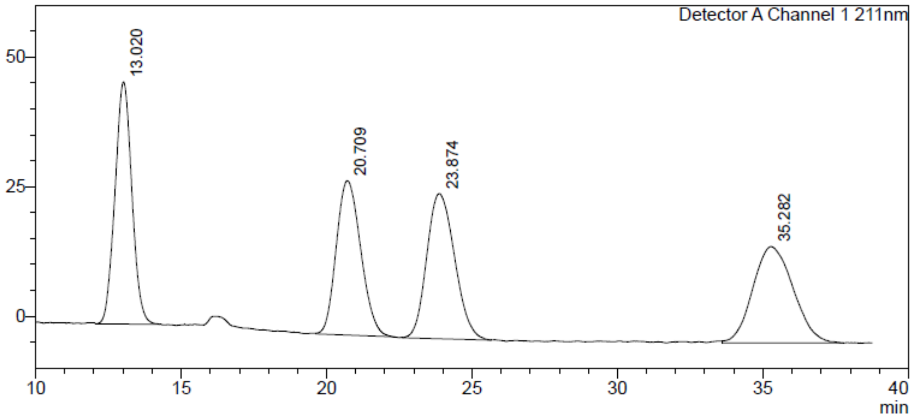

Peak#	Ret. Time	Area%
1	13.020	25.926
2	20.709	23.926
3	23.874	25.588
4	35.282	24.559
Total		100.000

Detector A Channel 1 211nm

19:

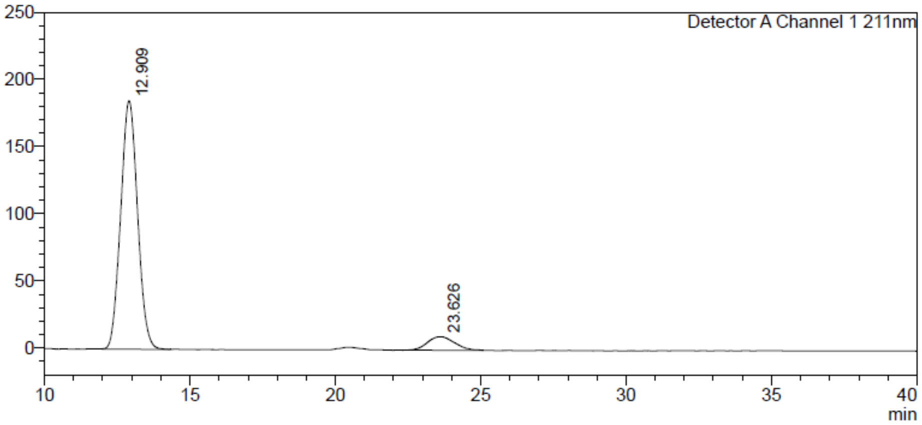

Detector A Channel 1 211nm

Peak#	Ret. Time	Area%
1	12.909	91.969
2	23.626	8.031
Total		100.000

20:

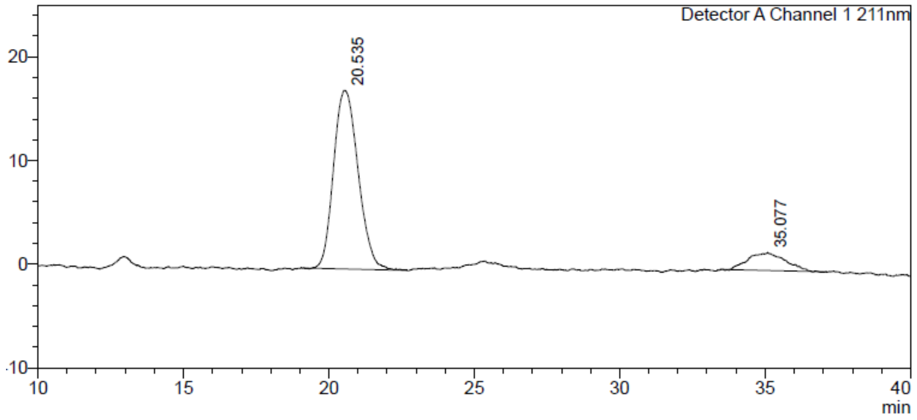

Detector A Channel 1 211nm

Peak#	Ret. Time	Area%
1	20.535	87.056
2	35.077	12.944
Total		100.000

HPLC data for **26**: Chiralpak AD-H (4.5% *i*-PrOH/hexane, flow rate 1.5 mLmin^{-1}, 211 nm, 40 °C), t_R major: 9.8 min and 18.6 min, 92:8 er, t_R minor: 13.1 min and 14.7 min, 87:13 er.

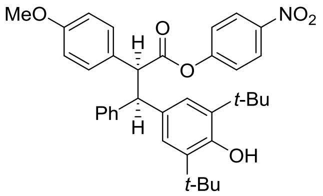

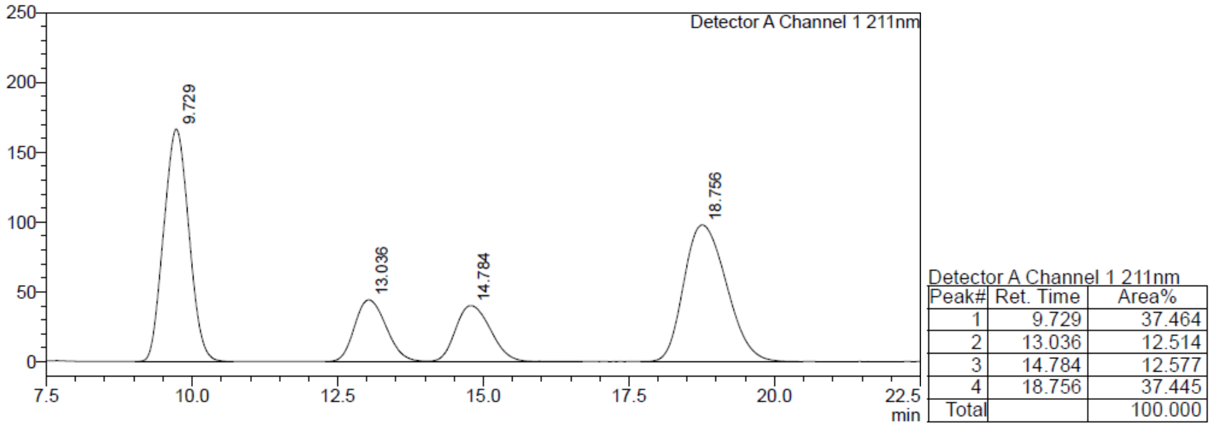

Detector A Channel 1 211nm

Peak#	Ret. Time	Area%
1	9.729	37.464
2	13.036	12.514
3	14.784	12.577
4	18.756	37.445
Total		100.000

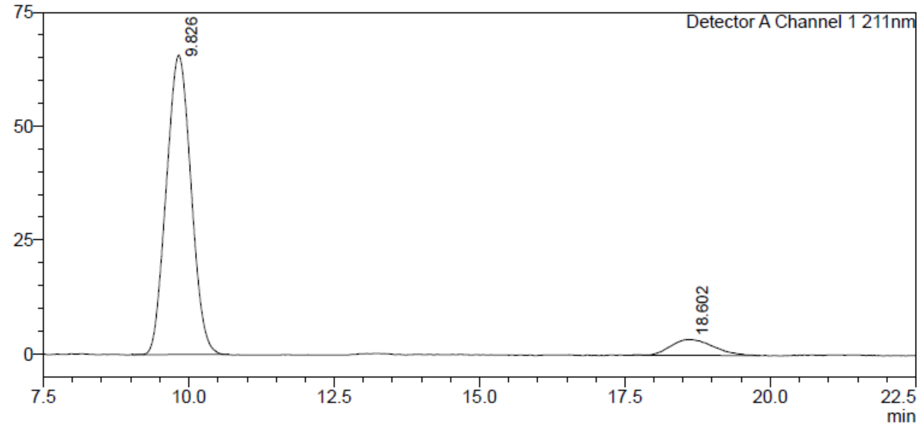

Detector A Channel 1 211nm

Peak#	Ret. Time	Area%
1	9.826	91.631
2	18.602	8.369
Total		100.000

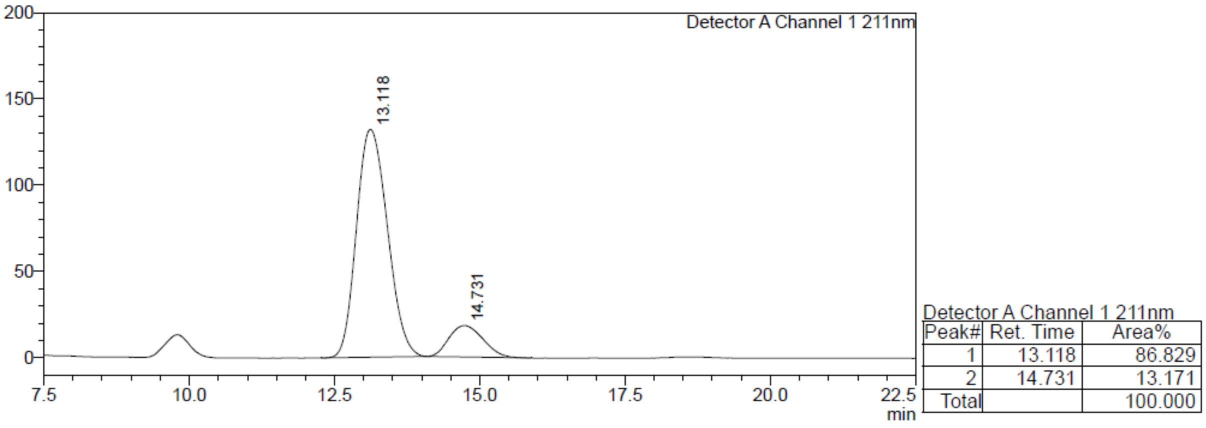

Detector A Channel 1 211nm

Peak#	Ret. Time	Area%
1	13.118	86.829
2	14.731	13.171
Total		100.000

HPLC data for **27**: Chiralpak AD-H (0.8% *i*-PrOH/hexane, flow rate 1.5 mLmin^{-1}, 254 nm, 30 °C), t_R major: 28.4 min and 83.8 min, 88:12 er, t_R minor: 29.4 min and 42.8 min, 79:21 er.

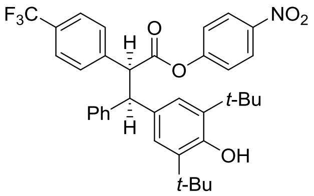

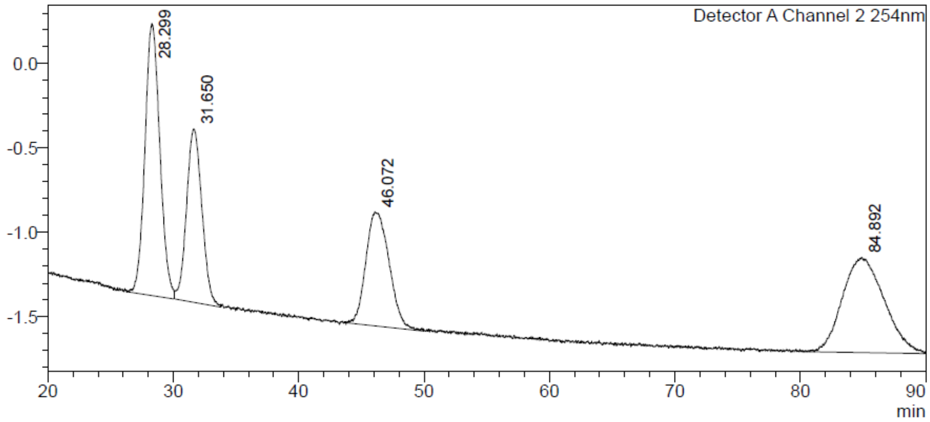

Detector A Channel 2 254nm

Peak#	Ret. Time	Area%
1	28.299	30.410
2	31.650	19.941
3	46.072	19.877
4	84.892	29.772
Total		100.000

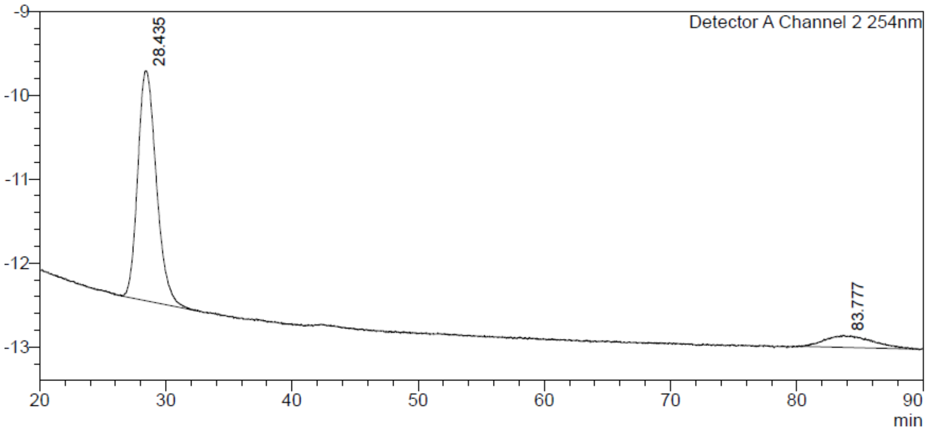

Detector A Channel 2 254nm

Peak#	Ret. Time	Area%
1	28.435	88.213
2	83.777	11.787
Total		100.000

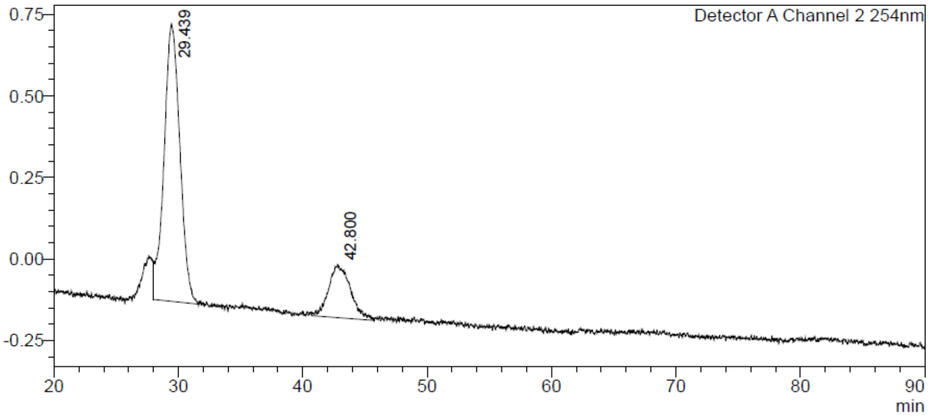

Detector A Channel 2 254nm

Peak#	Ret. Time	Area%
1	29.439	79.245
2	42.800	20.755
Total		100.000

HPLC data for **28**: Chiralpak AD-H (1.5% *i*-PrOH/hexane, flow rate 1.5 mLmin^{-1}, 211 nm, 30 °C), t_R major: 27.9 min and 53.4 min, 91:9 er, t_R minor: 22.6 min and 36.7 min, 85:15 er.

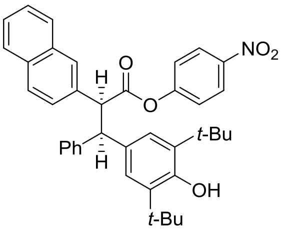

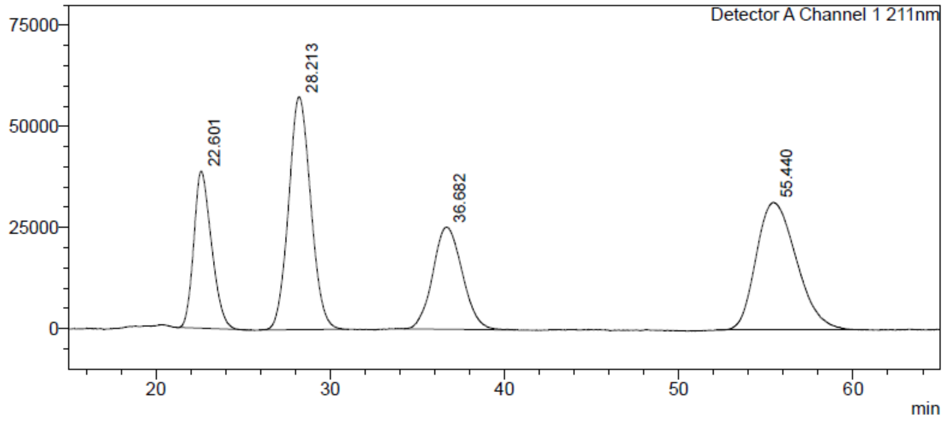

Detector A Channel 1 211nm

Peak#	Ret. Time	Area%
1	22.601	17.286
2	28.213	32.051
3	36.682	18.769
4	55.440	31.894
Total		100.000

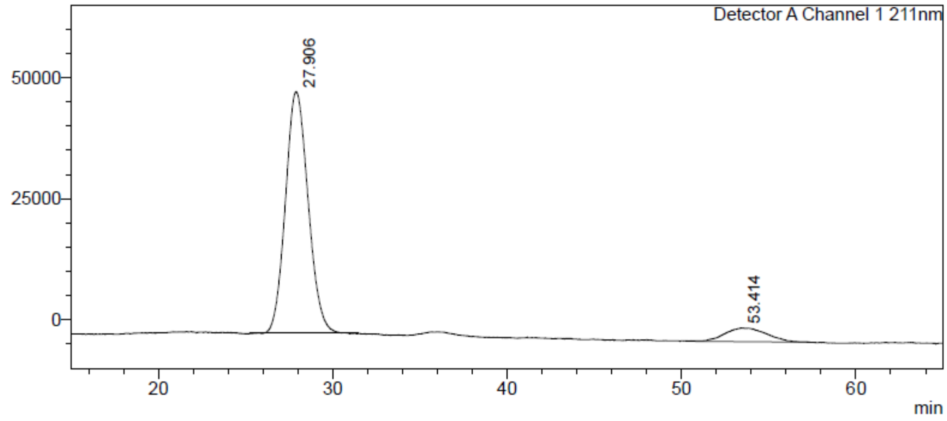

Detector A Channel 1 211nm

Peak#	Ret. Time	Area%
1	27.906	90.603
2	53.414	9.397
Total		100.000

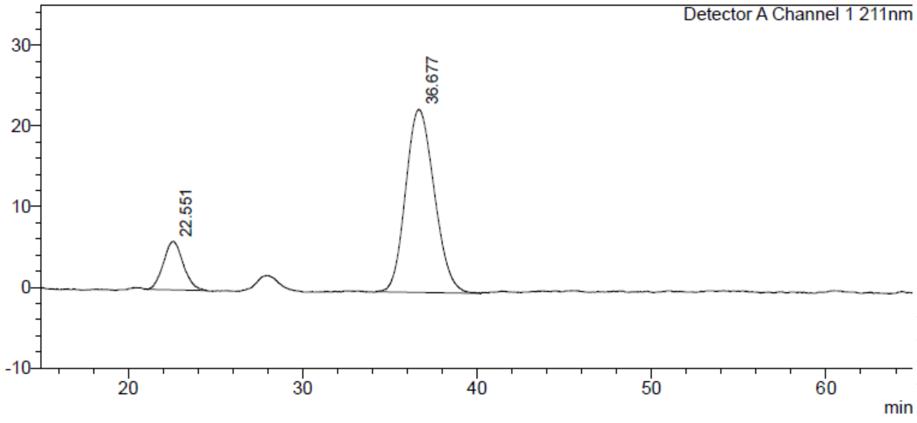

Detector A Channel 1 211nm

Peak#	Ret. Time	Area%
1	22.551	15.497
2	36.677	84.503
Total		100.000

HPLC data for **29**: Chiralcel OD-H (2% *i*-PrOH/hexane, flow rate 1.5 mLmin^{-1}, 211 nm, 30 °C), t_R major: 9.8 min and 21.2 min, 93:7 er, t_R minor: 6.1 min and 28.2 min, 94:6 er.

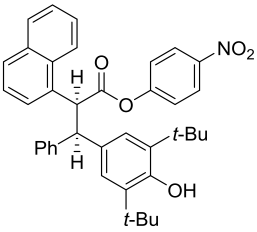

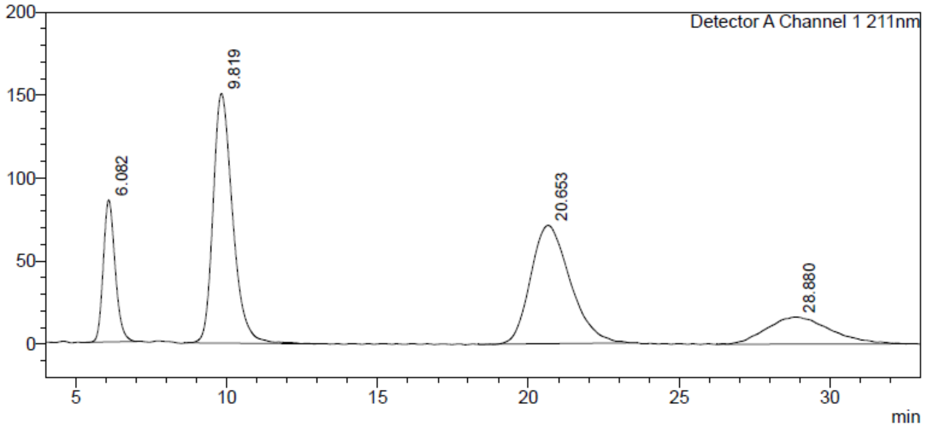

Detector A Channel 1 211nm

Peak#	Ret. Time	Area%
1	6.082	12.995
2	9.819	37.714
3	20.653	36.038
4	28.880	13.253
Total		100.000

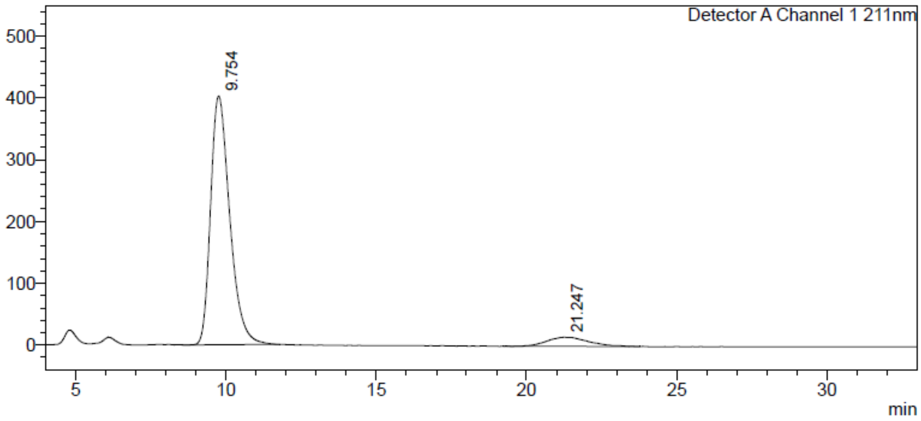

Detector A Channel 1 211nm

Peak#	Ret. Time	Area%
1	9.754	92.747
2	21.247	7.253
Total		100.000

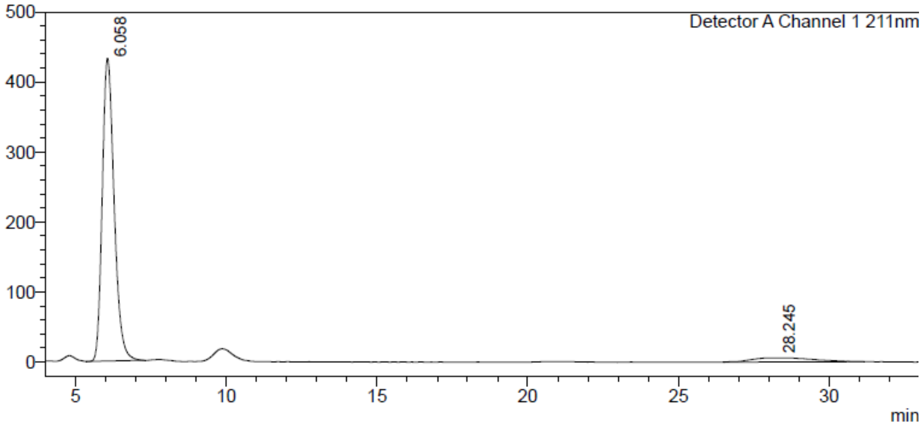

Detector A Channel 1 211nm

Peak#	Ret. Time	Area%
1	6.058	93.537
2	28.245	6.463
Total		100.000

HPLC data for **30**: Chiralpak AD-H (1.5% *i*-PrOH/hexane, flow rate 1.5 mLmin^{-1}, 211 nm, 30 °C), t_R major: 19.6 min and 28.7 min, 88:12 er, t_R minor: 22.6 min and 24.0 min, 90:10 er.

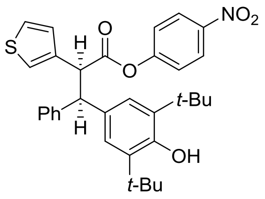

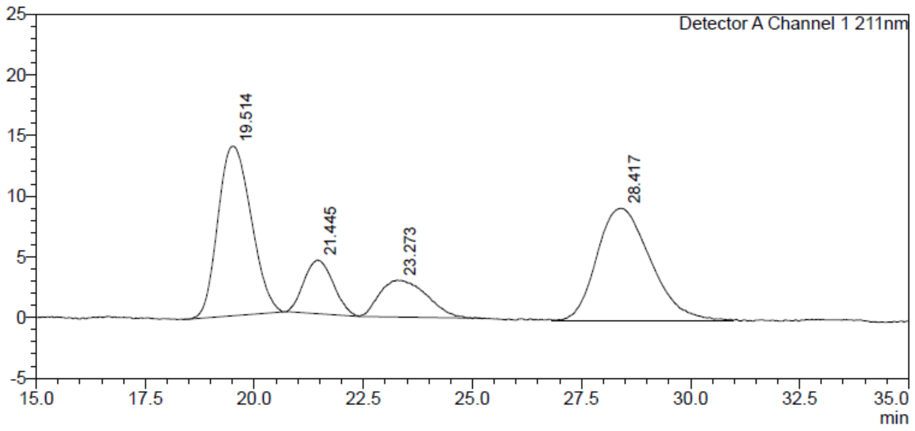

Detector A Channel 1 211nm

Peak#	Ret. Time	Area%
1	19.514	37.830
2	21.445	10.553
3	23.273	11.573
4	28.417	40.044
Total		100.000

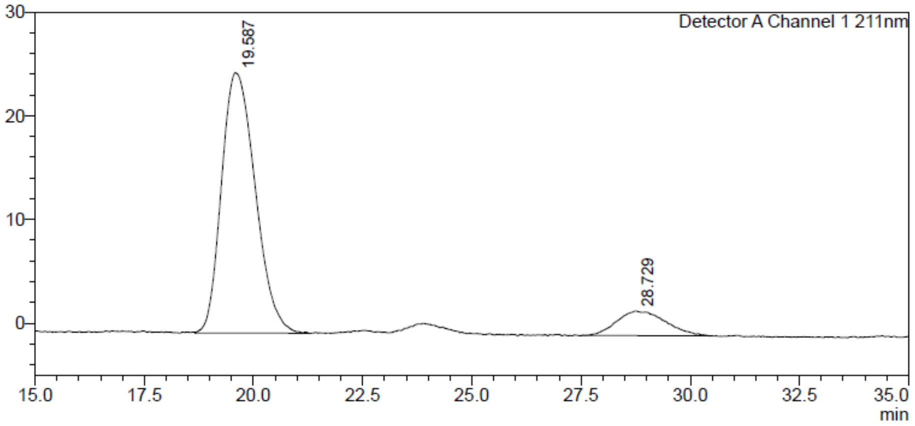

Detector A Channel 1 211nm

Peak#	Ret. Time	Area%
1	19.587	88.037
2	28.729	11.963
Total		100.000

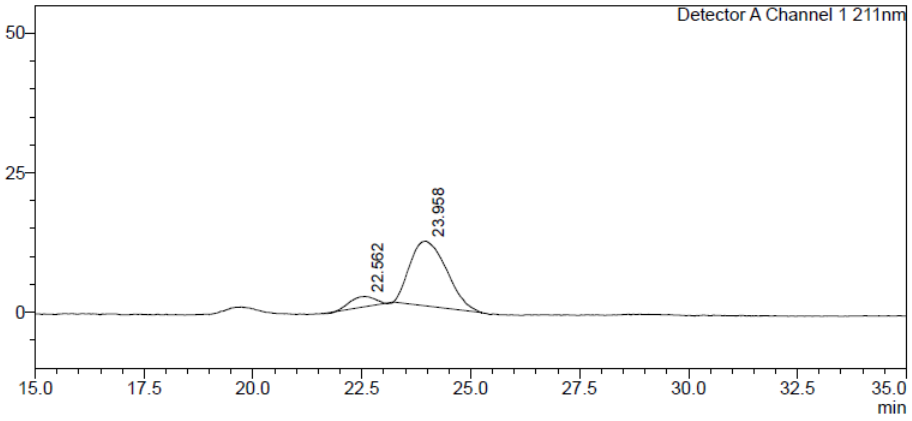

Detector A Channel 1 211nm

Peak#	Ret. Time	Area%
1	22.562	9.540
2	23.958	90.460
Total		100.000

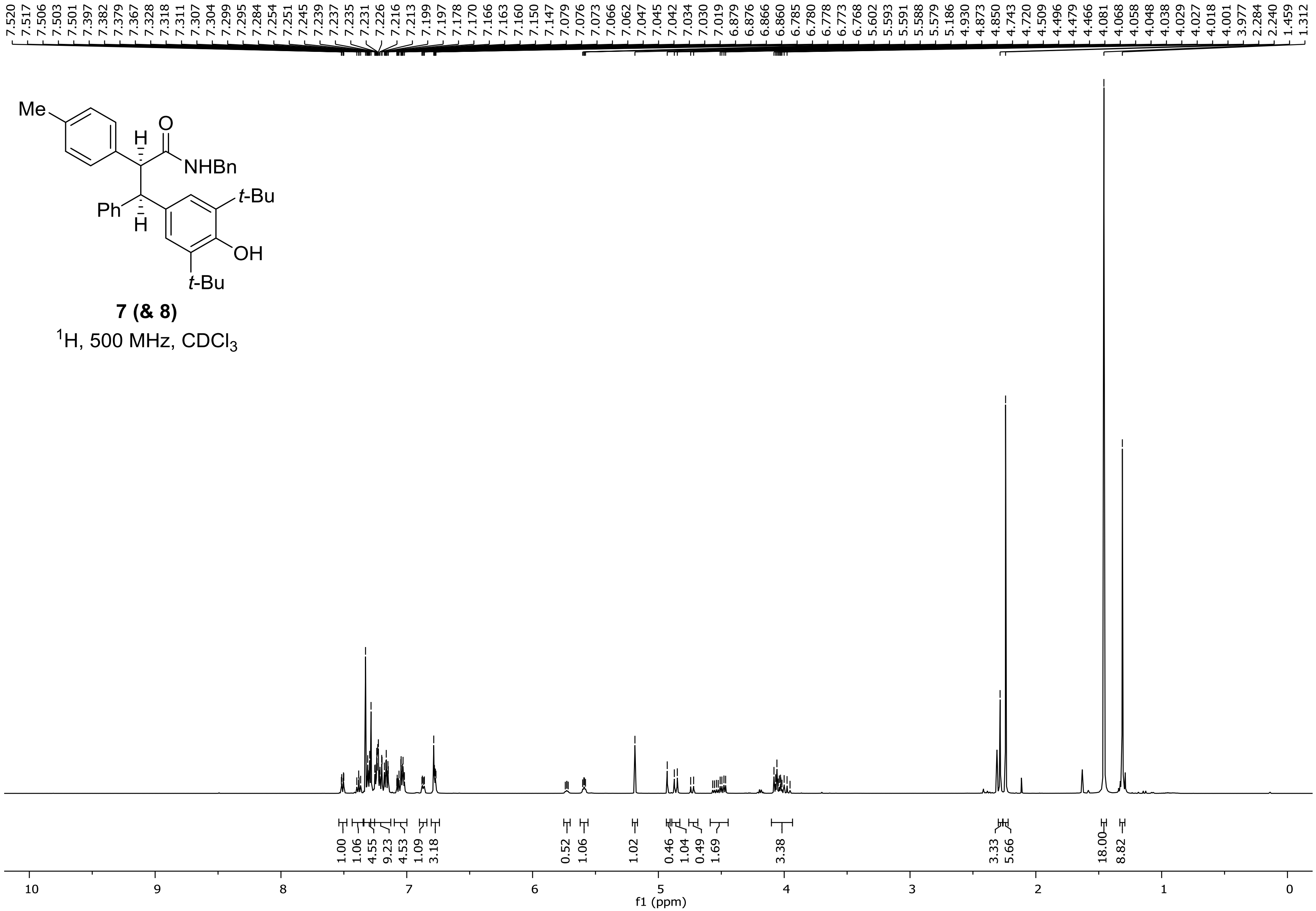
7.520 7.517 7.506 7.503 7.501 7.397 7.382 7.379 7.367 7.328 7.318 7.311 7.307 7.304 7.299 7.295 7.284 7.254 7.251 7.245 7.239 7.237 7.235 7.231 7.226 7.216 7.213 7.199 7.197 7.178 7.170 7.166 7.163 7.160 7.150 7.147 7.079 7.076 7.073 7.066 7.062 7.047 7.045 7.042 7.034 7.030 7.019 6.879 6.876 6.866 6.860 6.785 6.780 6.778 6.773 6.768 5.602 5.593 5.591 5.588 5.579 5.186 4.930 4.873 4.850 4.743 4.720 4.509 4.496 4.479 4.466 4.081 4.068 4.058 4.048 4.038 4.029 4.027 4.018 4.001 3.977 2.284 2.240 1.459 1.312
Me
H
O
NHBn
Ph
H
t-Bu
OH
t-Bu
7 (& 8)
^{1}H, 500 MHz, CDCl$_3$
1.00 1.06 4.55 9.23 4.53 1.09 3.18 0.52 1.06 1.02 0.46 1.04 0.49 1.69 3.38 3.33 5.66 18.00 8.82
10 9 8 7 6 5 4 3 2 1 0
f1 (ppm)

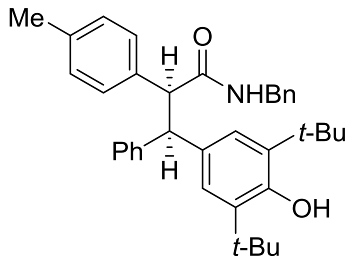

7 (& 8)

^{13}C, 126 MHz, $CDCl_3$

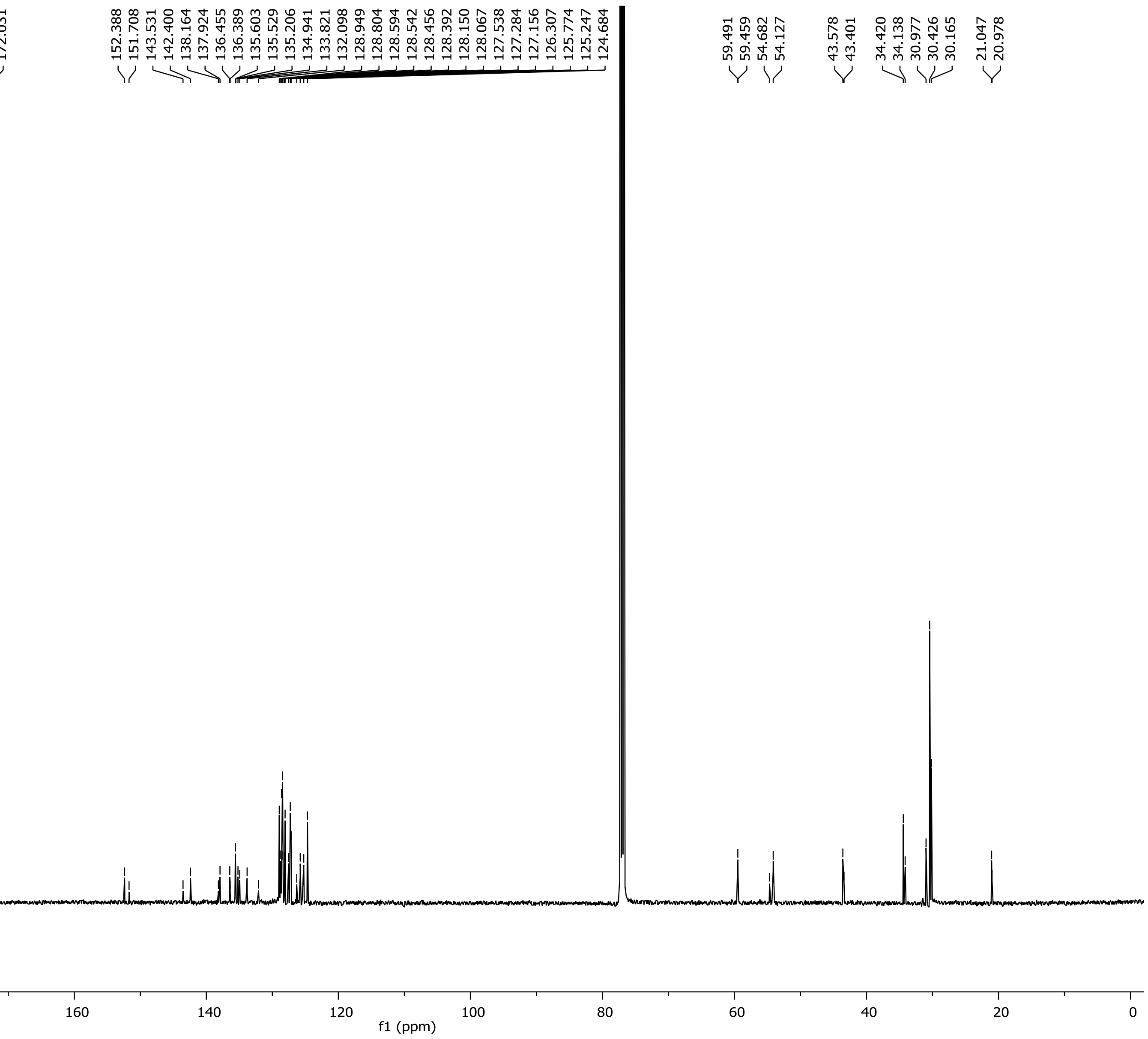

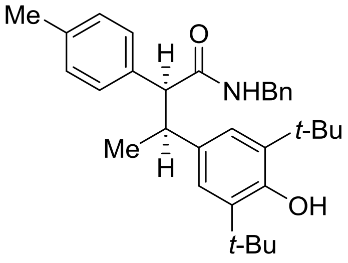

17

^{1}H, 400 MHz, $CDCl_3$

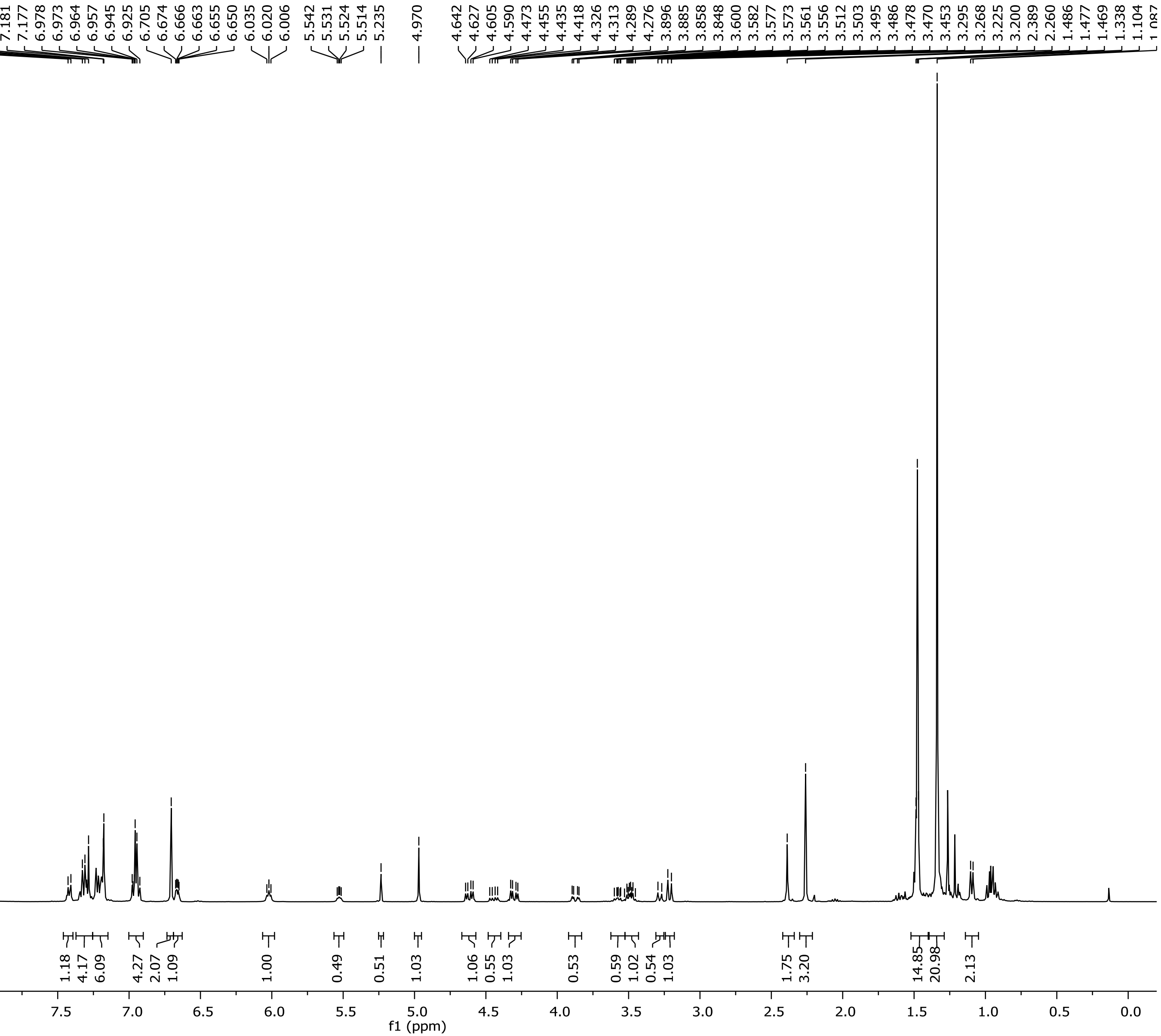

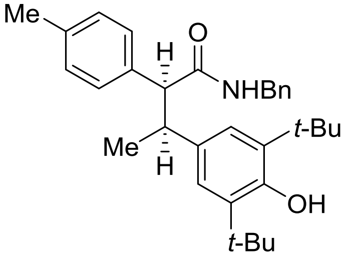

17

^{13}C, 101 MHz, $CDCl_3$

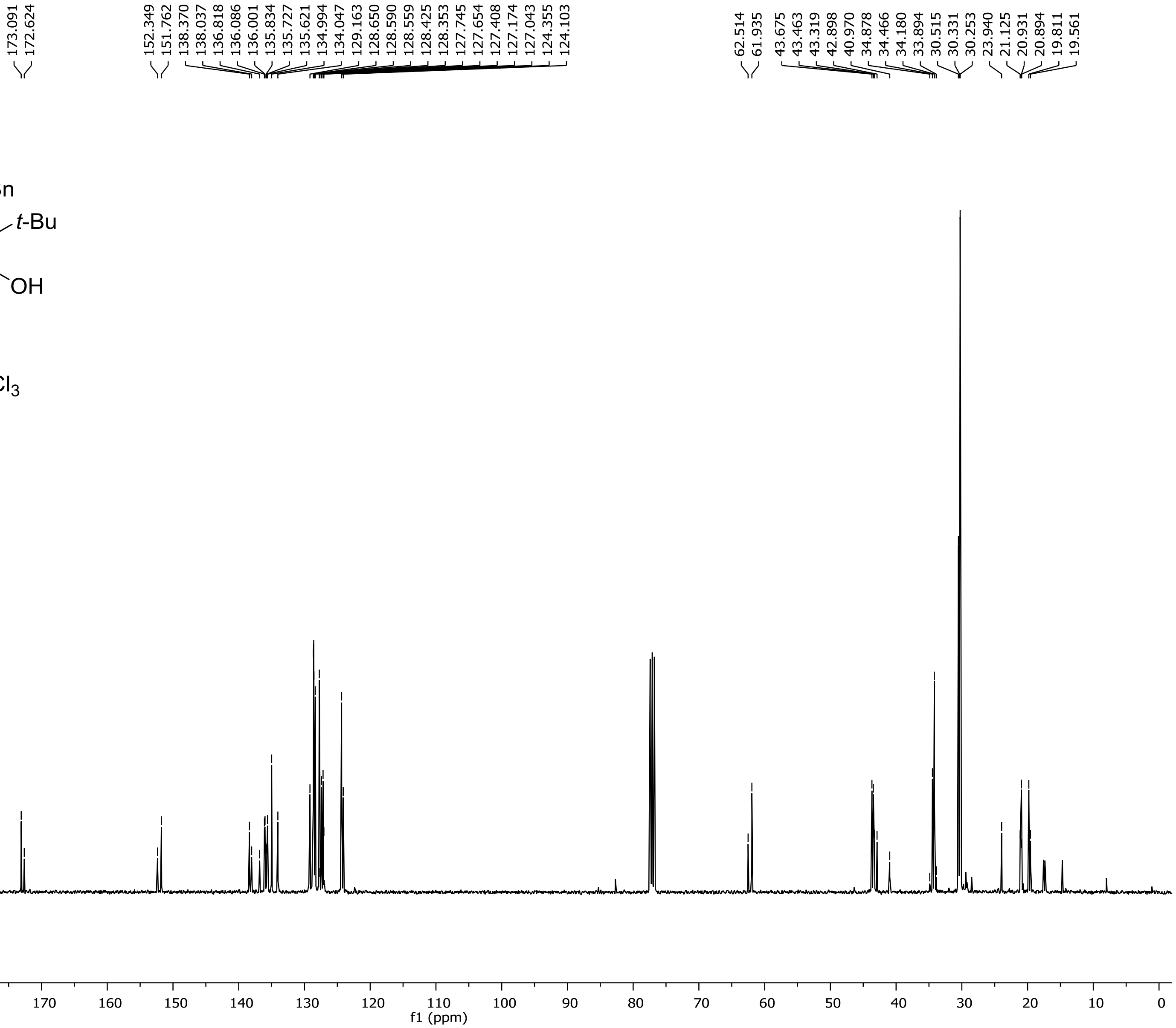

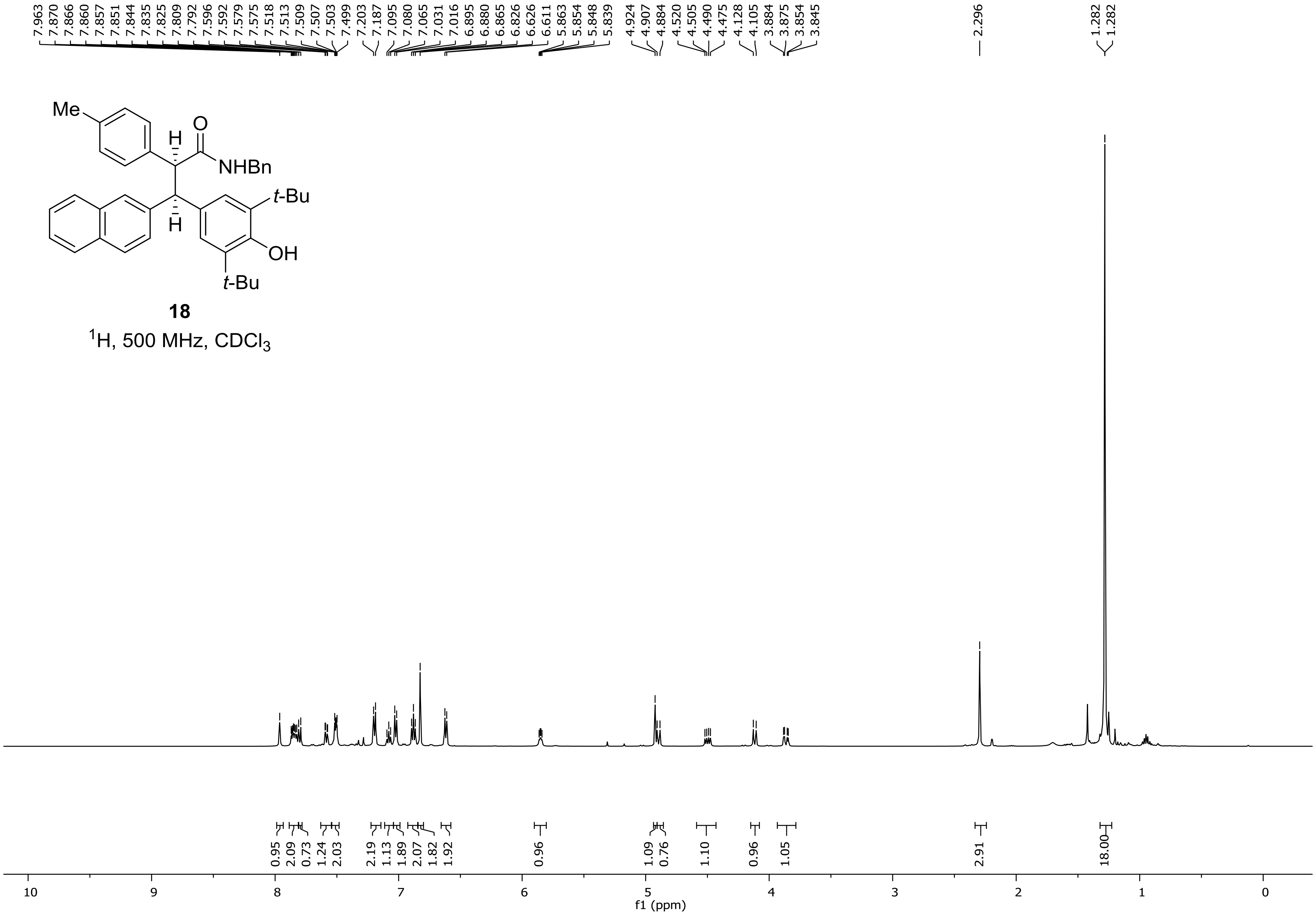

18

^{1}H, 500 MHz, $CDCl_3$

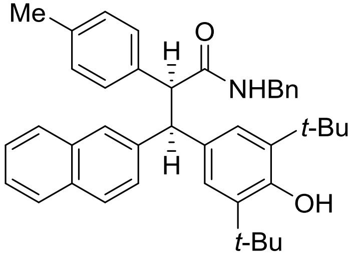

18

^{13}C, 126 MHz, $CDCl_3$

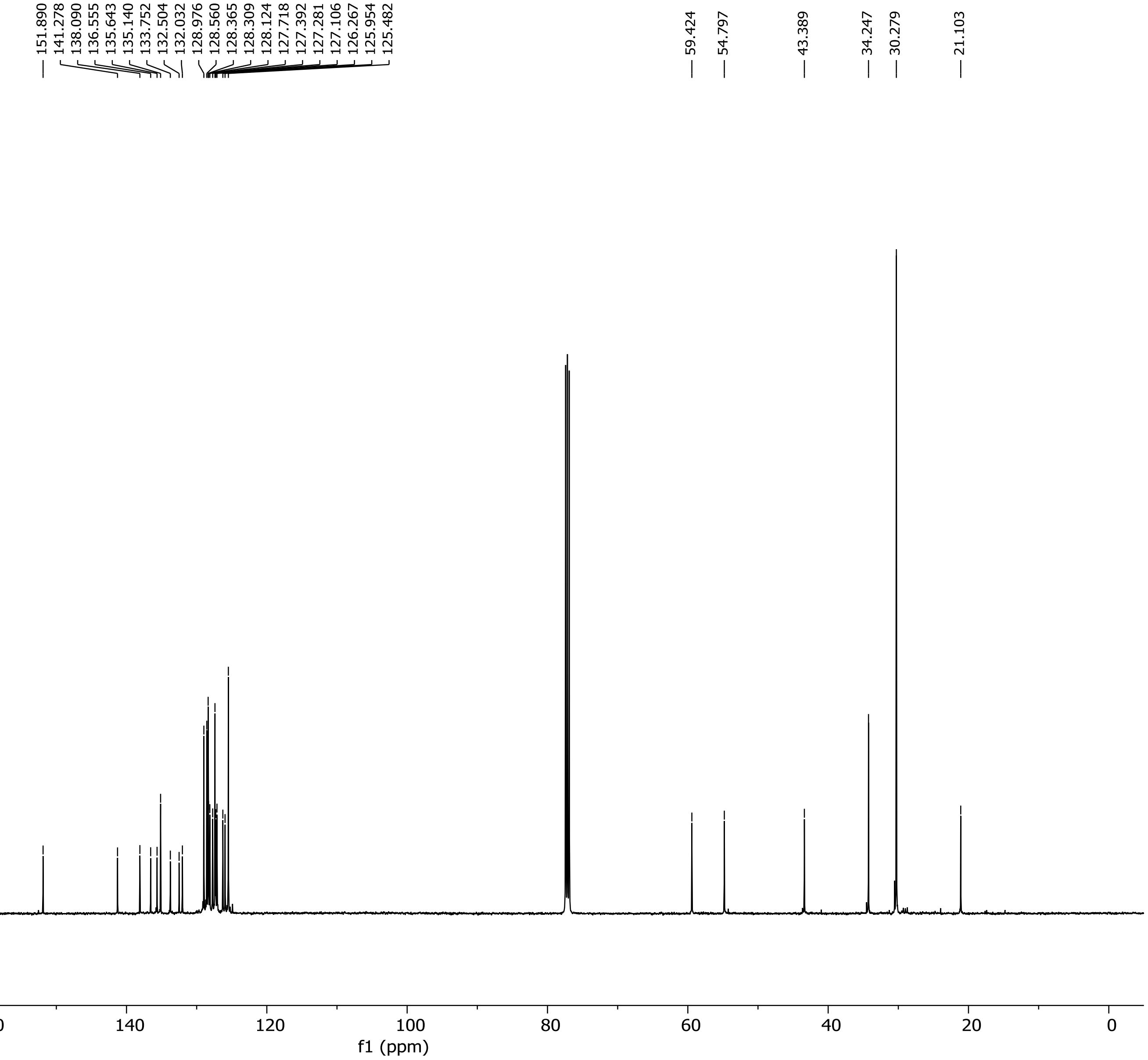

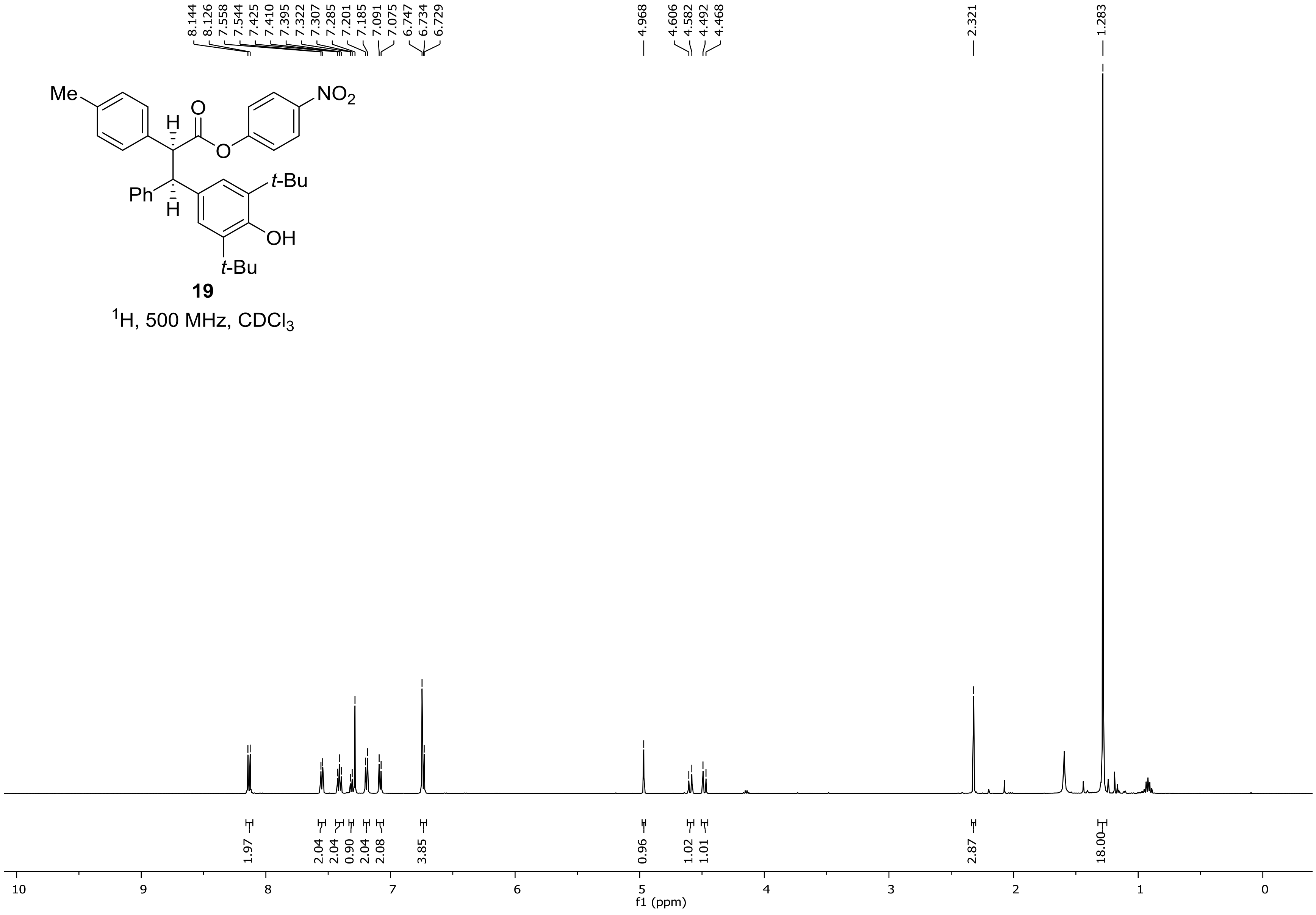

8.144
8.126
7.558
7.544
7.425
7.410
7.395
7.322
7.307
7.285
7.201
7.185
7.091
7.075
6.747
6.734
6.729
4.968
4.606
4.582
4.492
4.468
2.321
1.283
Me
NO2
H
O
O
Ph
H
t-Bu
OH
t-Bu
19
¹H, 500 MHz, CDCl3
1.97
2.04
2.04
0.90
2.04
2.08
3.85
0.96
1.02
1.01
2.87
18.00
10
9
8
7
6
5
4
3
2
1
0
f1 (ppm)

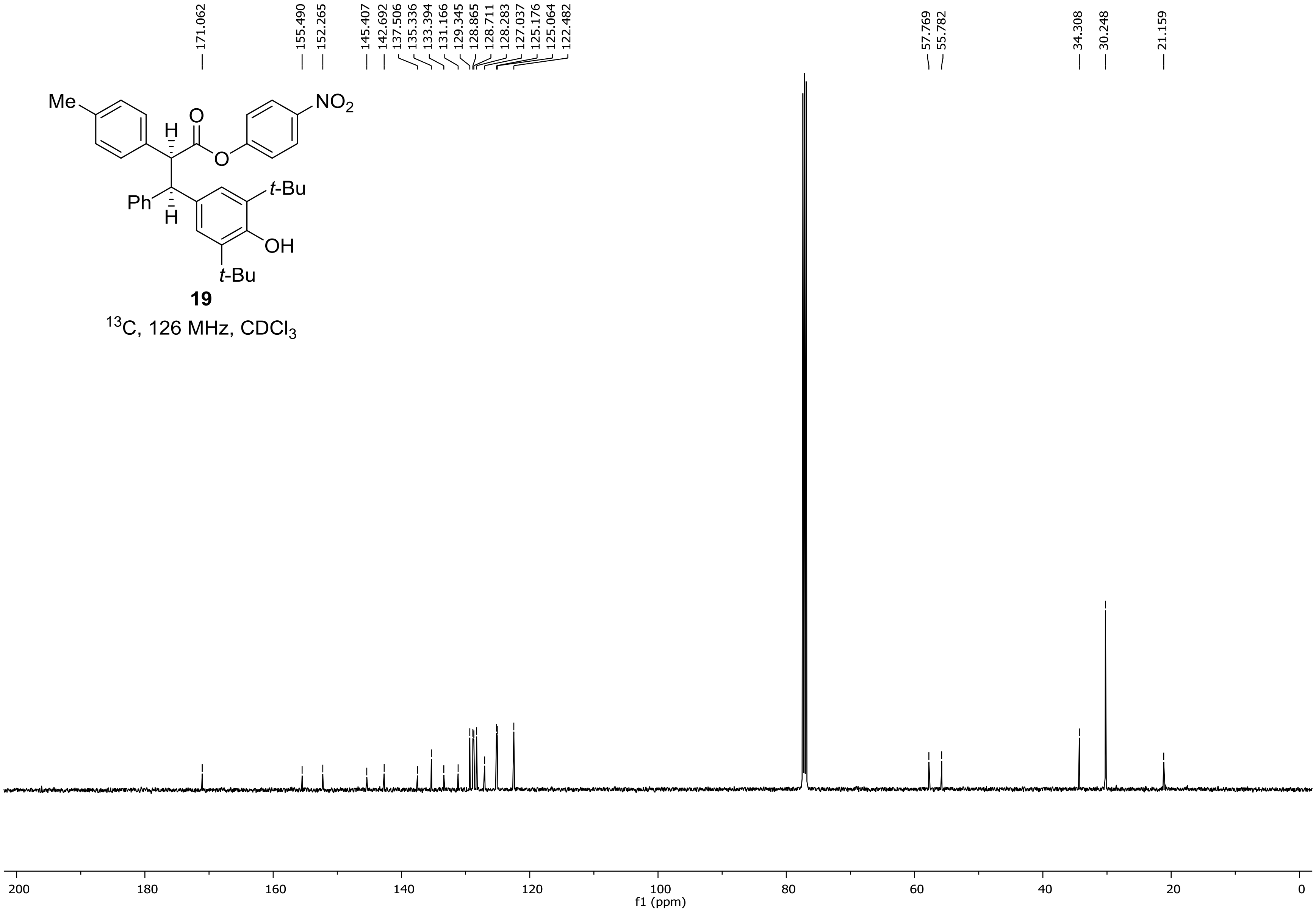
171.062
155.490
152.265
145.407
142.692
137.506
135.336
133.394
131.166
129.345
128.865
128.711
128.283
127.037
125.176
125.064
122.482
57.769
55.782
34.308
30.248
21.159
Me
NO_2
O
H
Ph
H
t-Bu
OH
t-Bu
19
^{13}C, 126 MHz, $CDCl_3$
200
180
160
140
120
100
80
60
40
20
0
f1 (ppm)

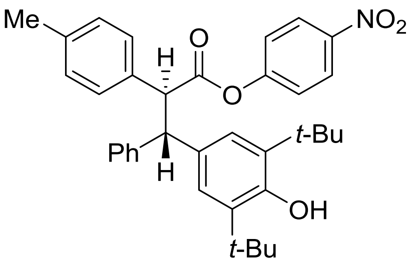

20

^{1}H, 500 MHz, $CDCl_3$

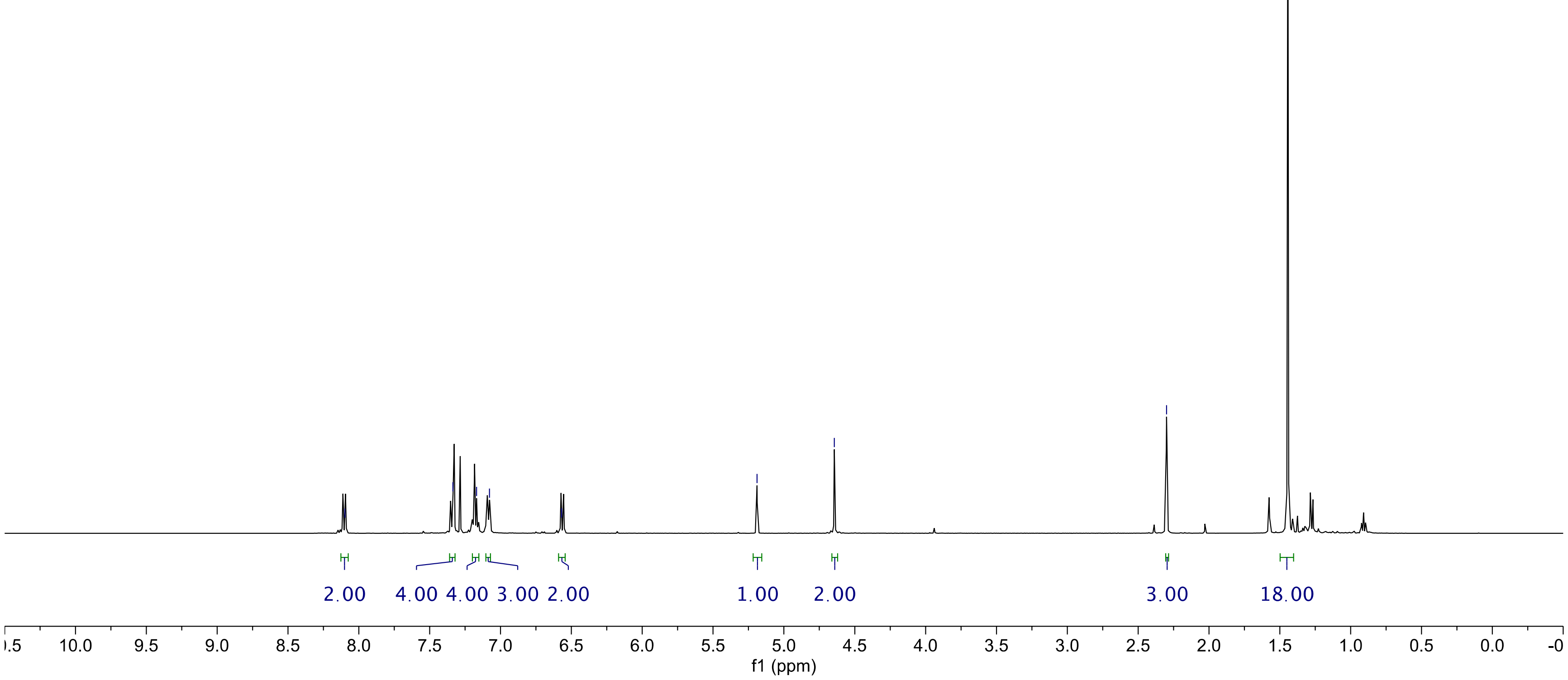

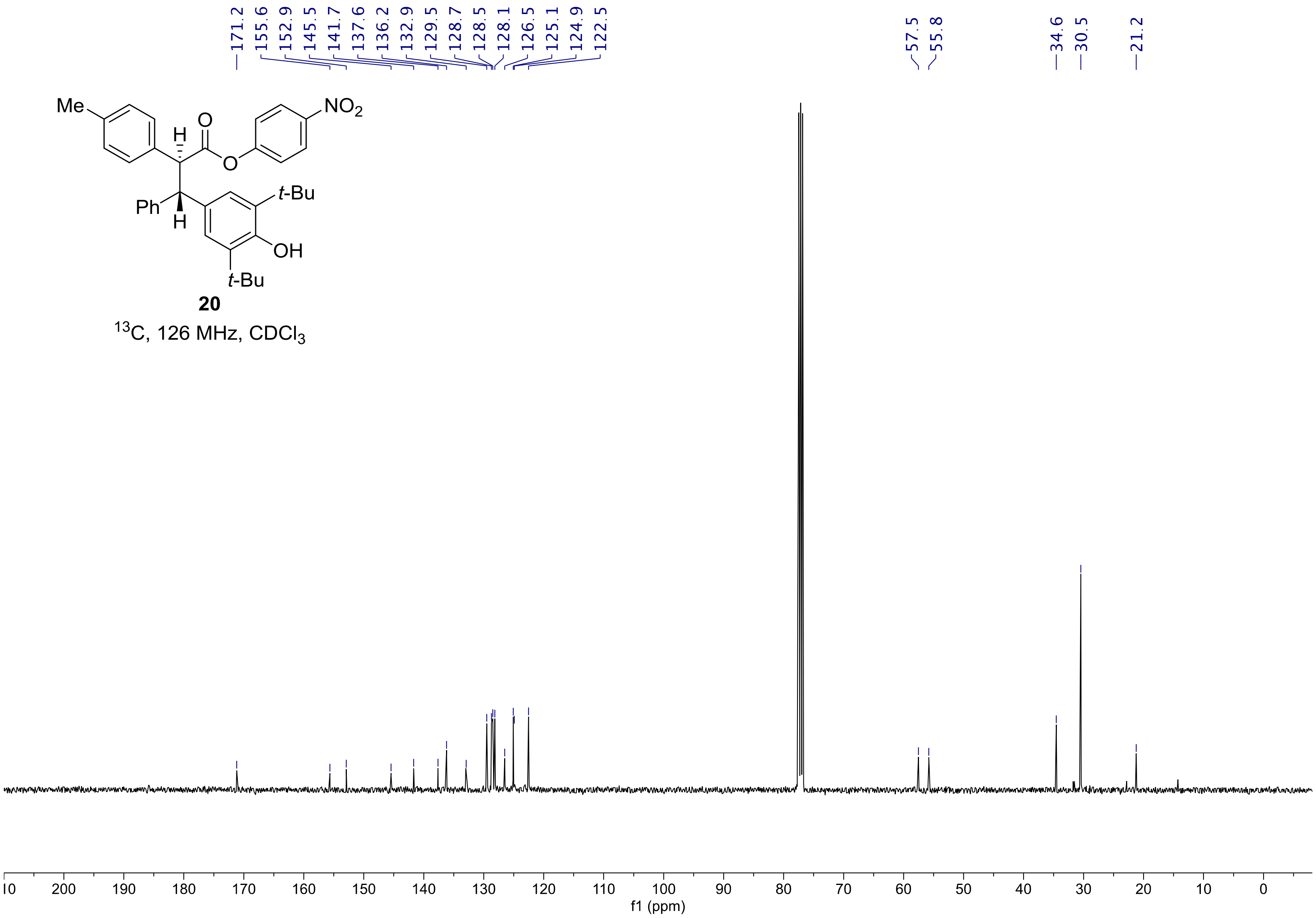

171.2
155.6
152.9
145.5
141.7
137.6
136.2
132.9
129.5
128.7
128.5
128.1
126.5
125.1
124.9
122.5
57.5
55.8
34.6
30.5
21.2
Me
NO2
O
H
O
Ph
H
t-Bu
OH
t-Bu
20
13C, 126 MHz, CDCl3
f1 (ppm)

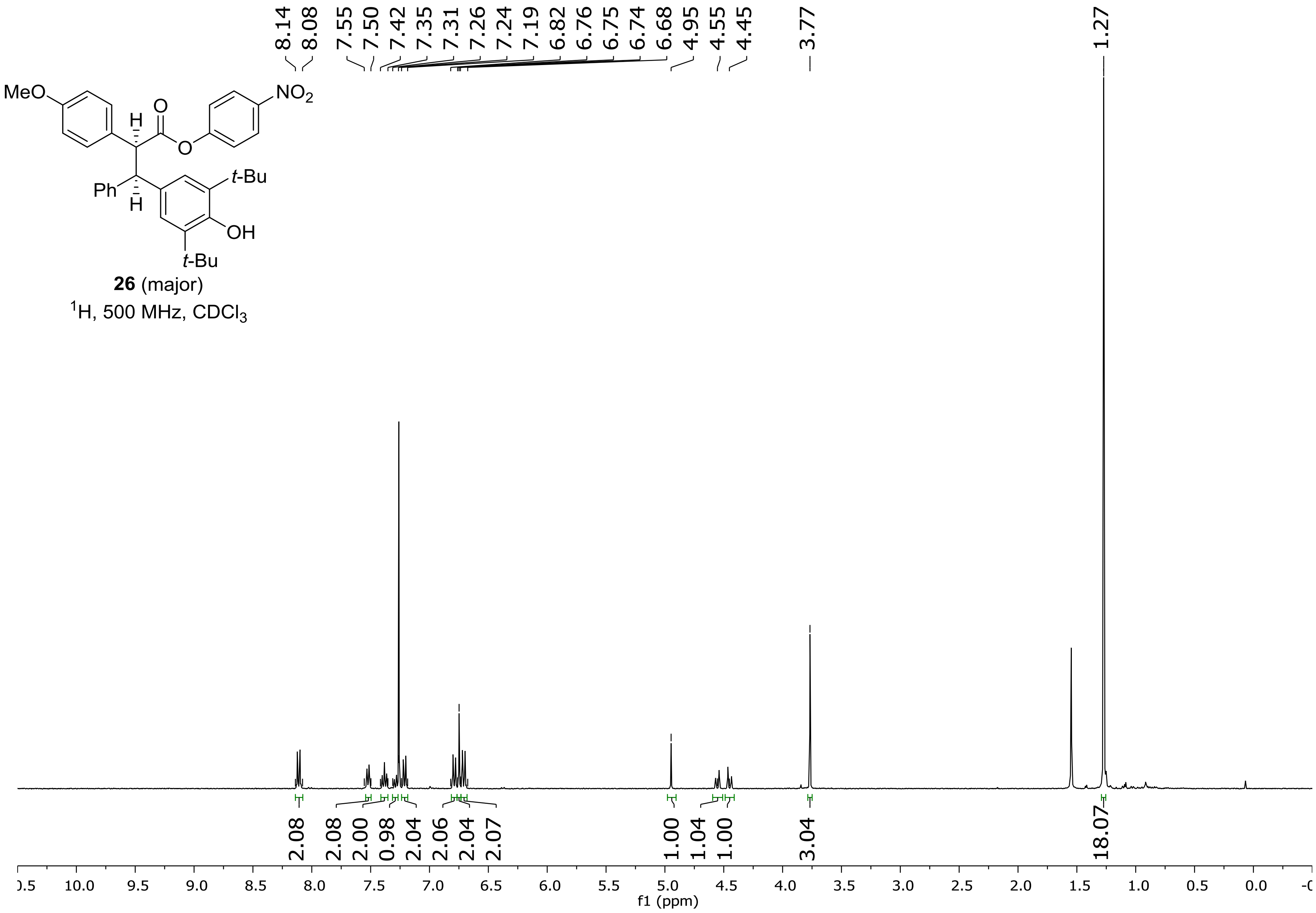
8.14
8.08
7.55
7.50
7.42
7.35
7.31
7.26
7.24
7.19
6.82
6.76
6.75
6.74
6.68
4.95
4.55
4.45
3.77
1.27
MeO
NO2
H
O
O
Ph
H
t-Bu
OH
t-Bu
26 (major)
1H, 500 MHz, CDCl3
2.08
2.08
2.00
0.98
2.04
2.06
2.04
2.07
1.00
1.04
1.00
3.04
18.07
f1 (ppm)

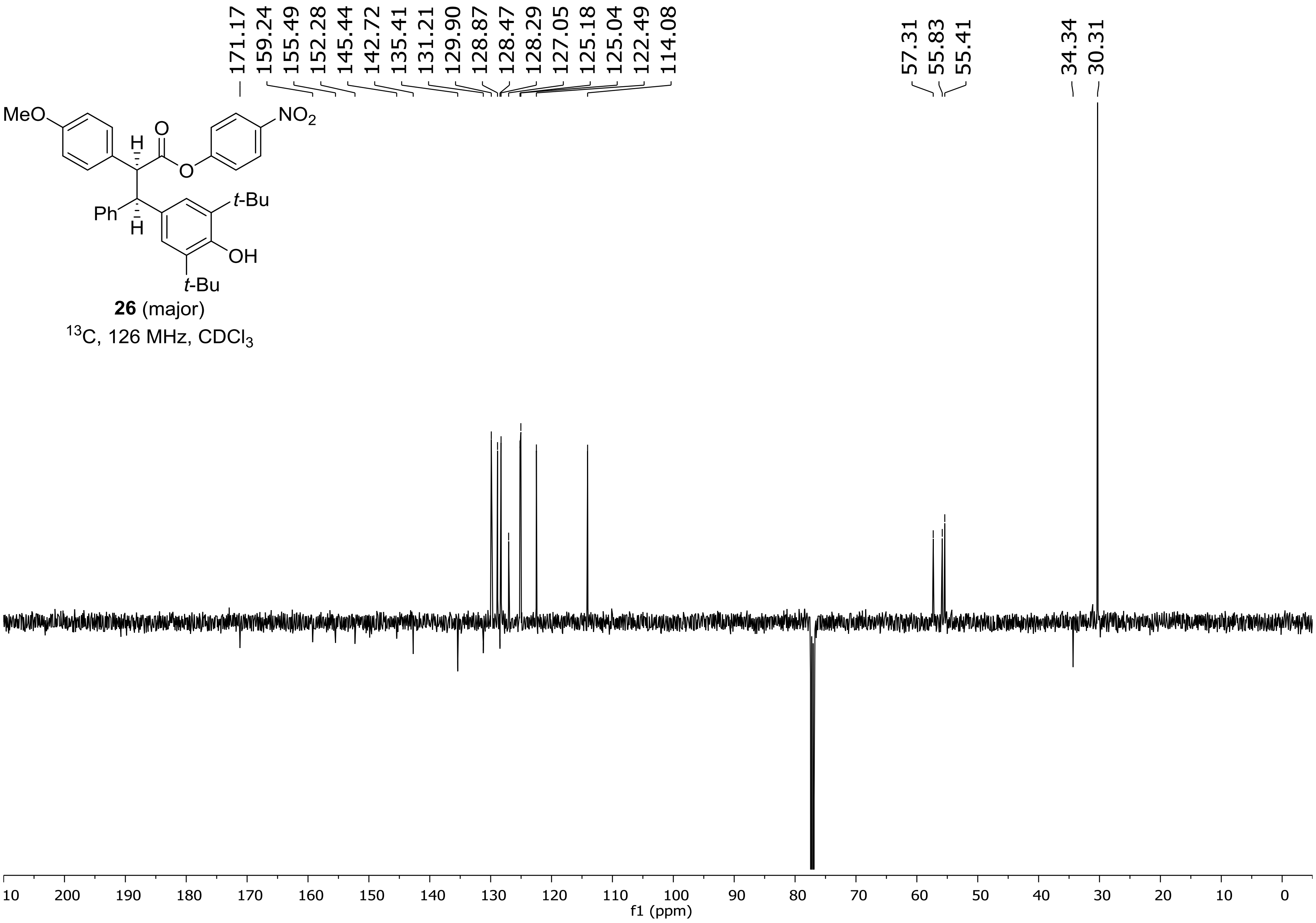
171.17
159.24
155.49
152.28
145.44
142.72
135.41
131.21
129.90
128.87
128.47
128.29
127.05
125.18
125.04
122.49
114.08
57.31
55.83
55.41
34.34
30.31
MeO
NO_2
H
O
O
Ph
H
t-Bu
OH
t-Bu
26 (major)
^{13}C, 126 MHz, $CDCl_3$
10 200 190 180 170 160 150 140 130 120 110 100 90 80 70 60 50 40 30 20 10 0
f1 (ppm)

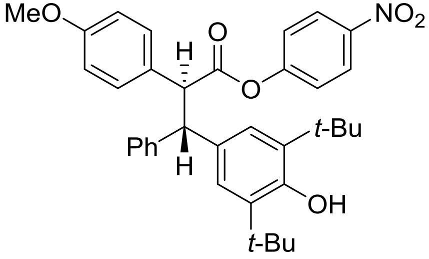

26 (minor)

^{1}H, 500 MHz, $CDCl_3$

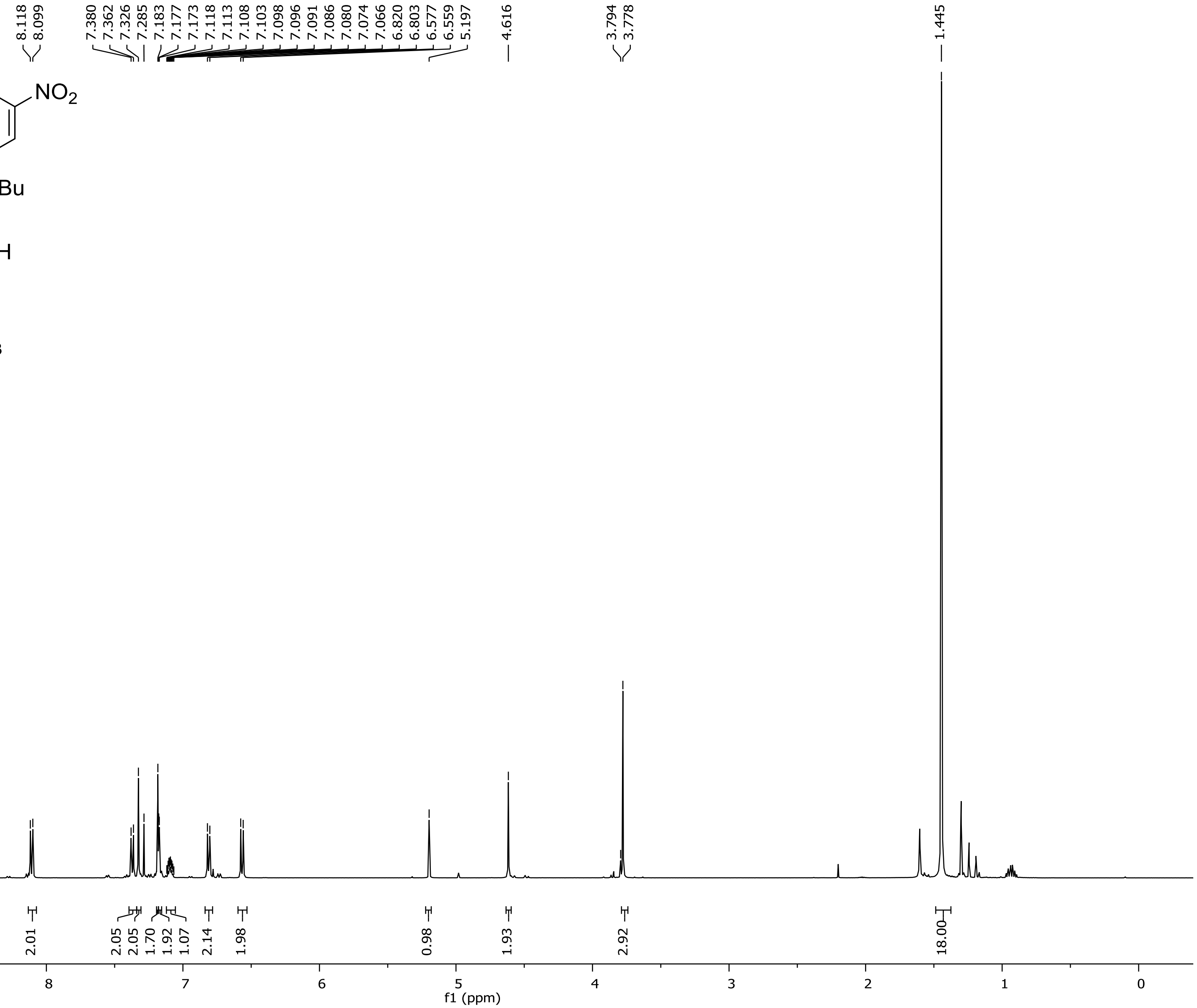

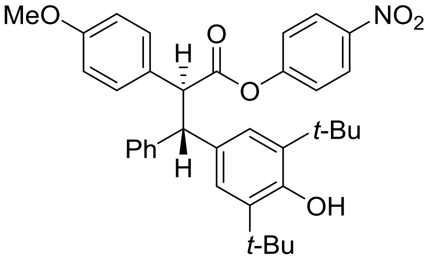

26 (minor)
^{13}C, 126 MHz, $CDCl_3$

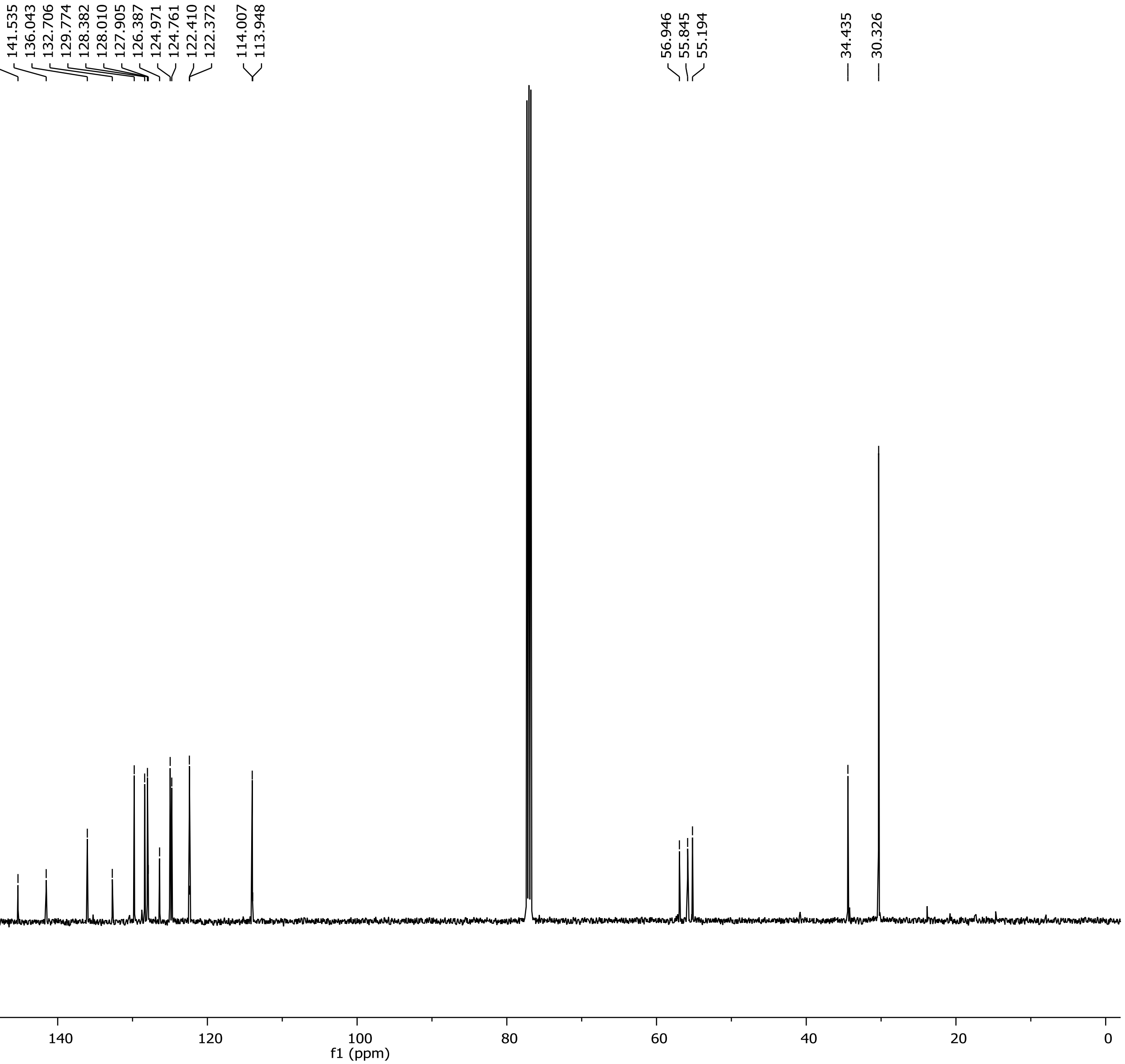

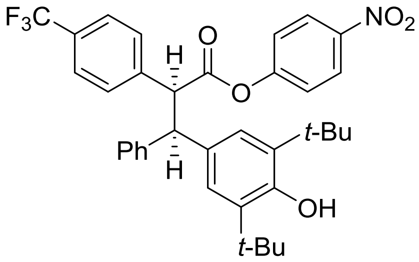

27 (major)
^1H, 500 MHz, $CDCl_3$

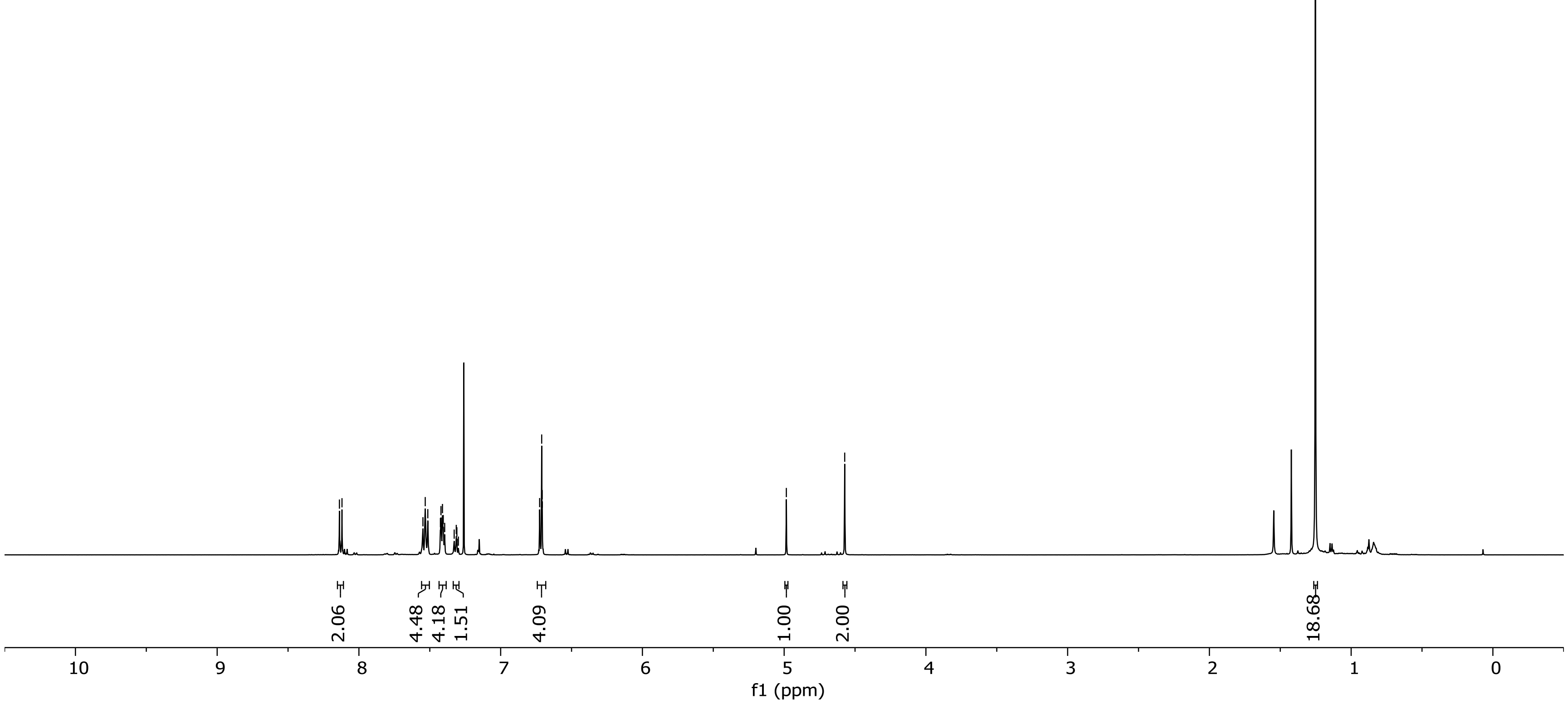

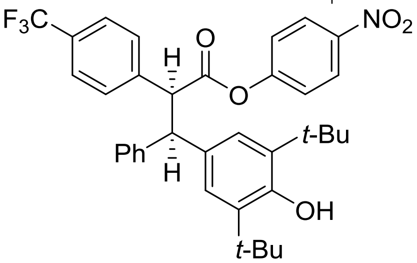

27 (major)

^{13}C, 126 MHz, $CDCl_3$

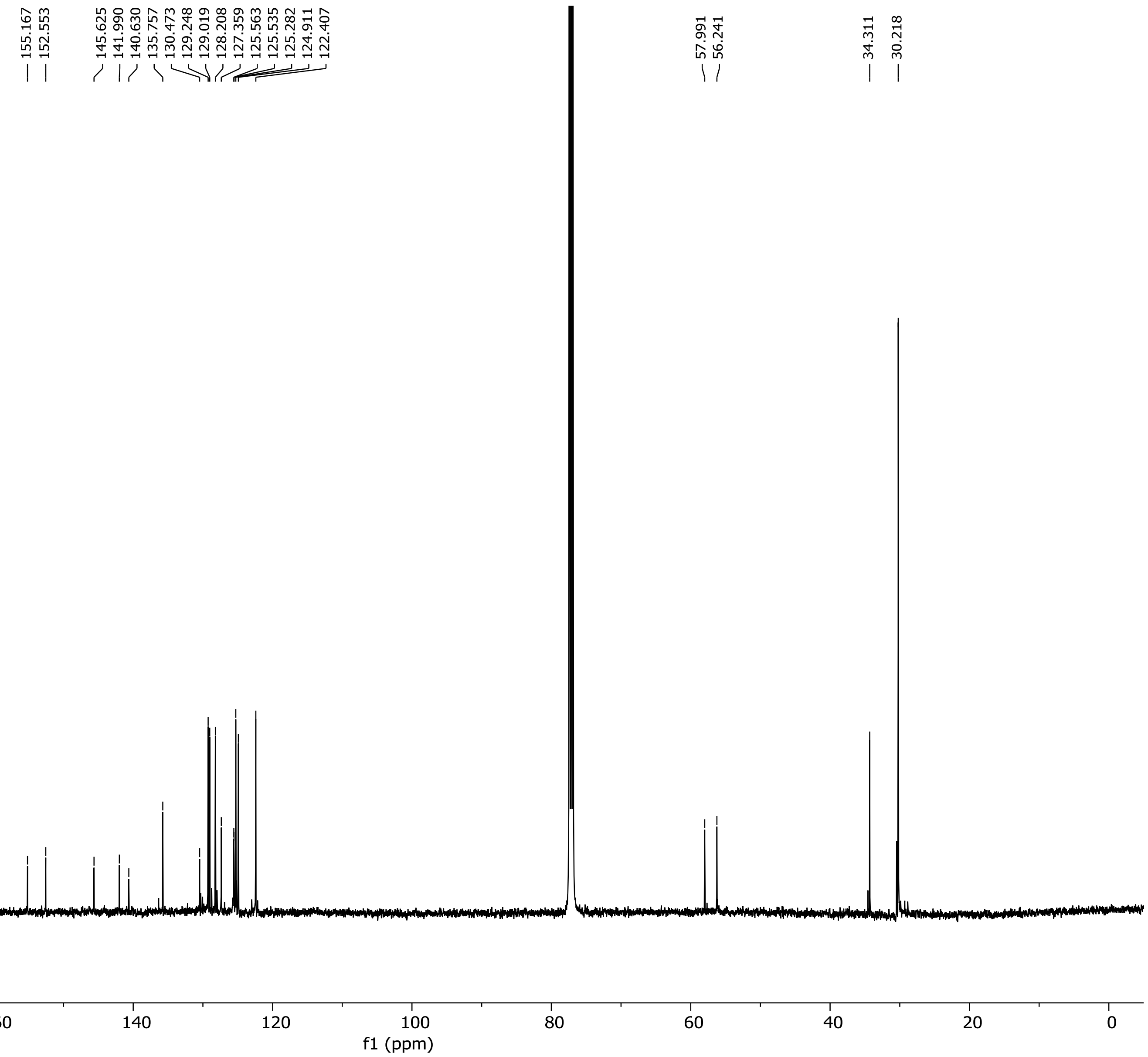

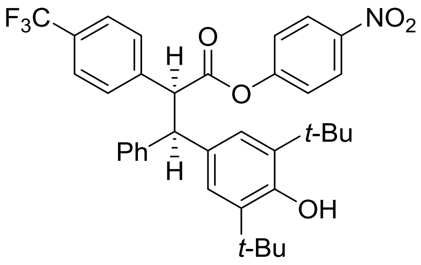

27 (major)

^{19}F, 376 MHz, $CDCl_3$

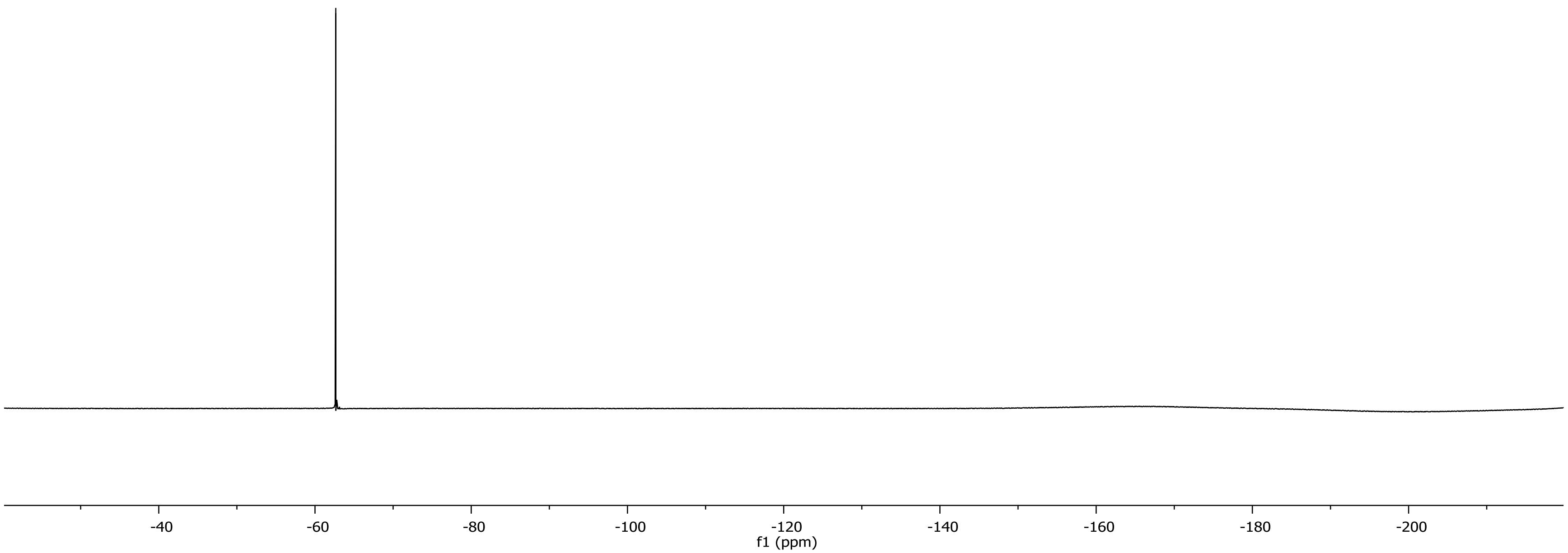

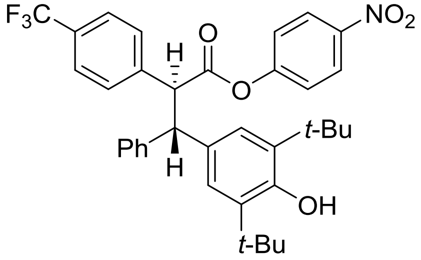

27 (minor)

^1H, 500 MHz, $CDCl_3$

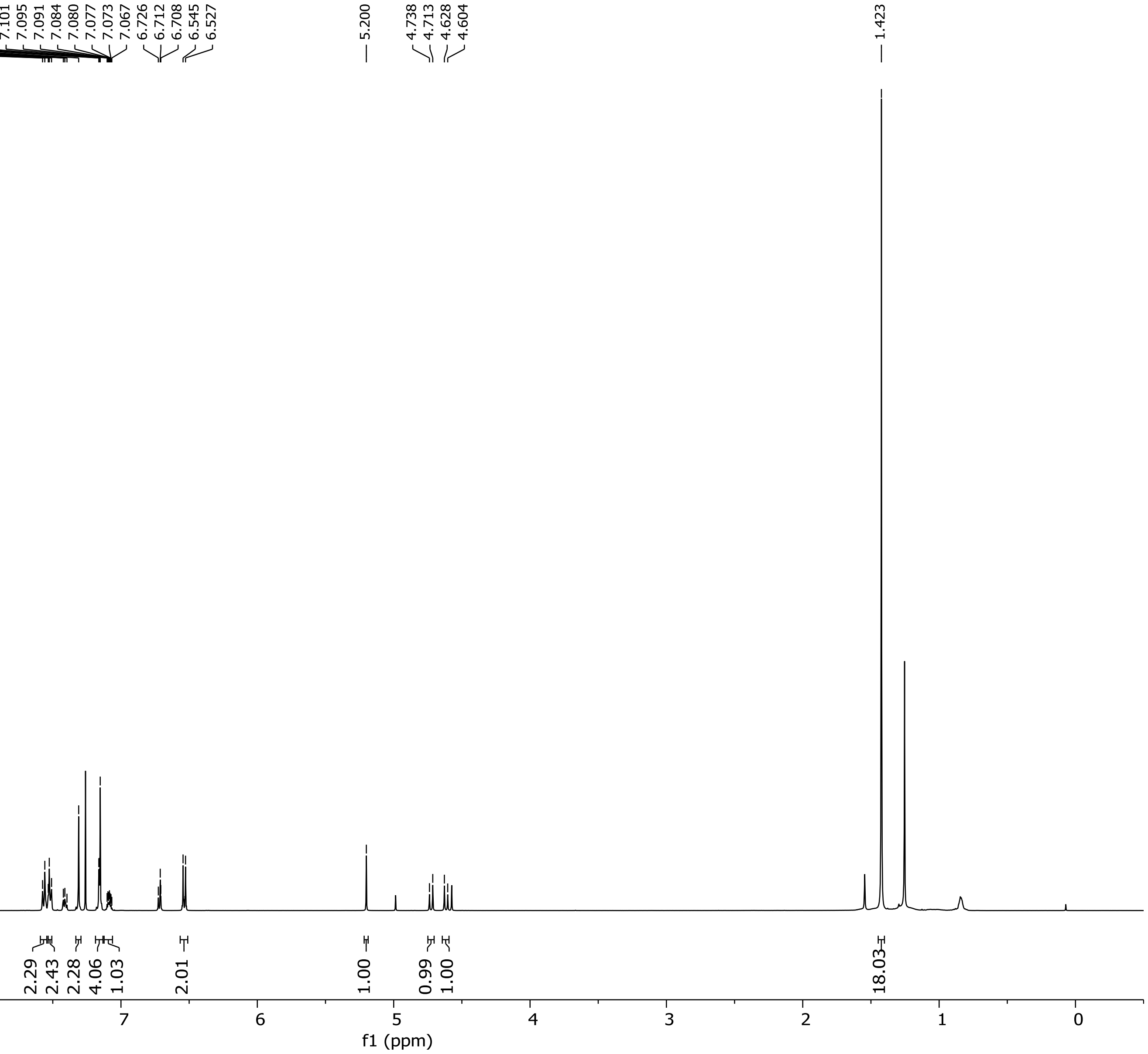

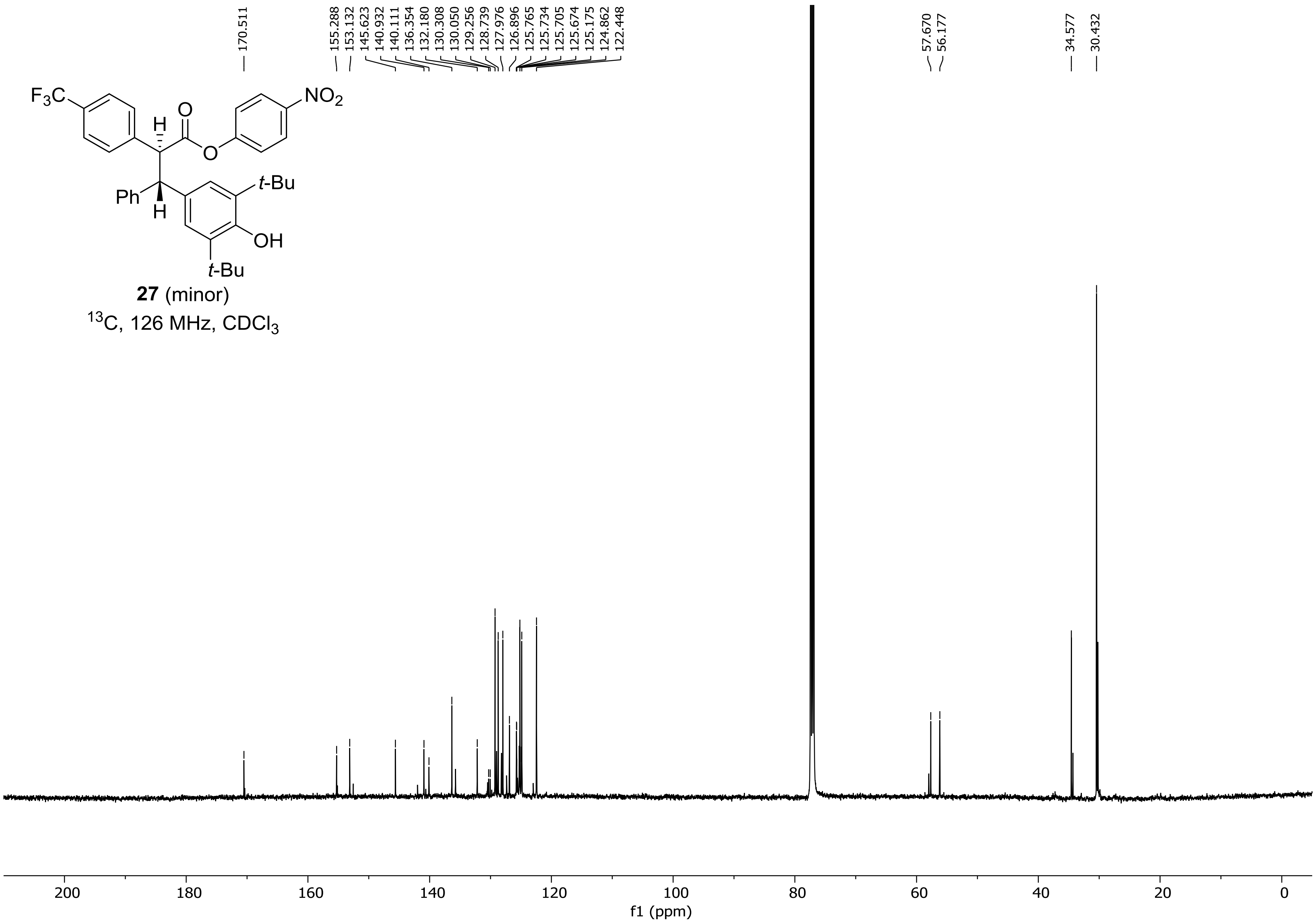
170.511
155.288
153.132
145.623
140.932
140.111
136.354
132.180
130.308
130.050
129.256
128.739
127.976
126.896
125.765
125.734
125.705
125.674
125.175
124.862
122.448
57.670
56.177
34.577
30.432
F_3C
NO_2
O
H
Ph
H
t-Bu
OH
t-Bu
27 (minor)
^{13}C, 126 MHz, $CDCl_3$
200
180
160
140
120
100
80
60
40
20
0
f1 (ppm)

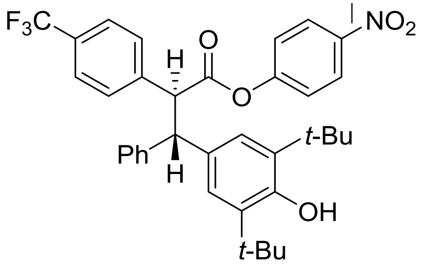

27 (minor)

^{19}F, 376 MHz, CDCl$_3$

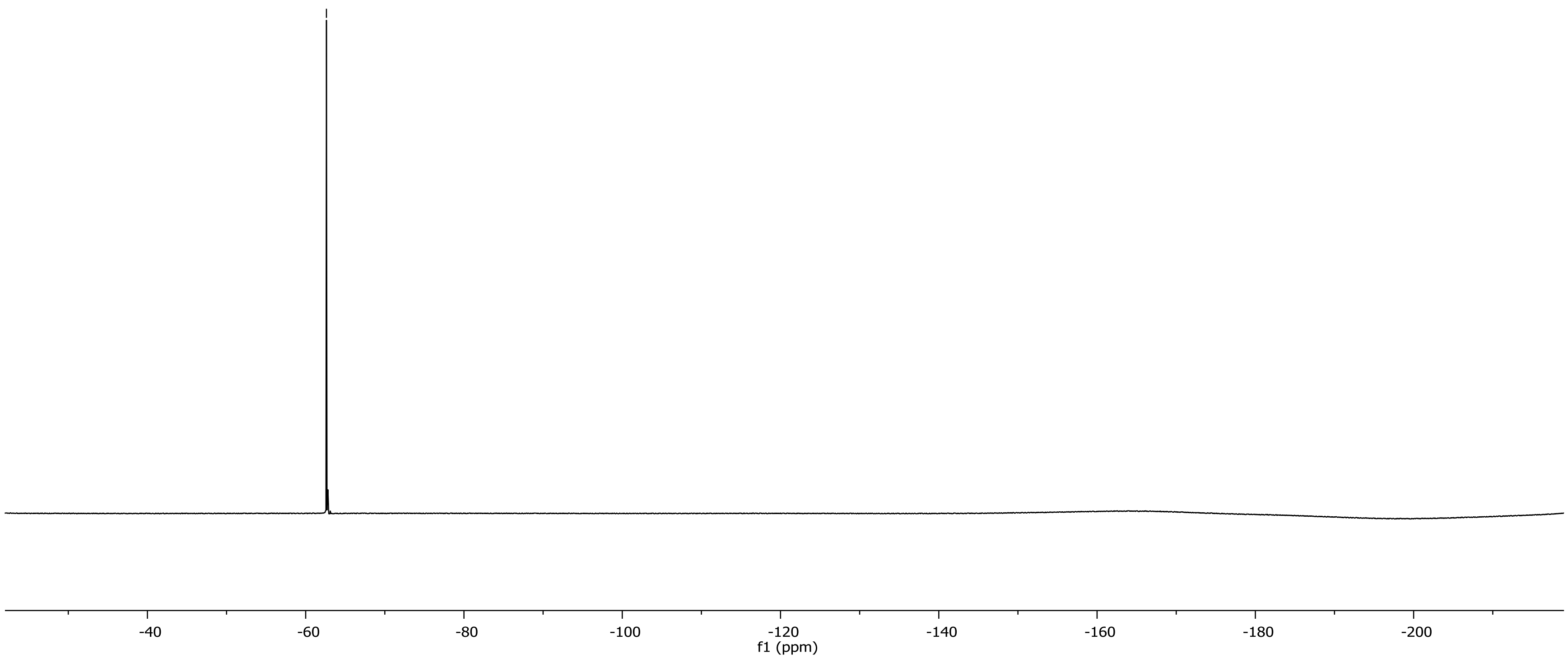

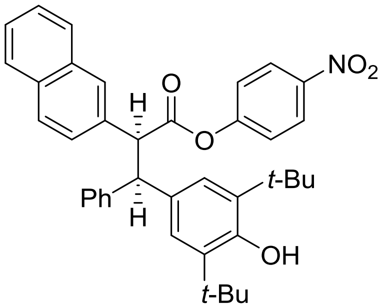

28 (major)

^{1}H, 500 MHz, $CDCl_3$

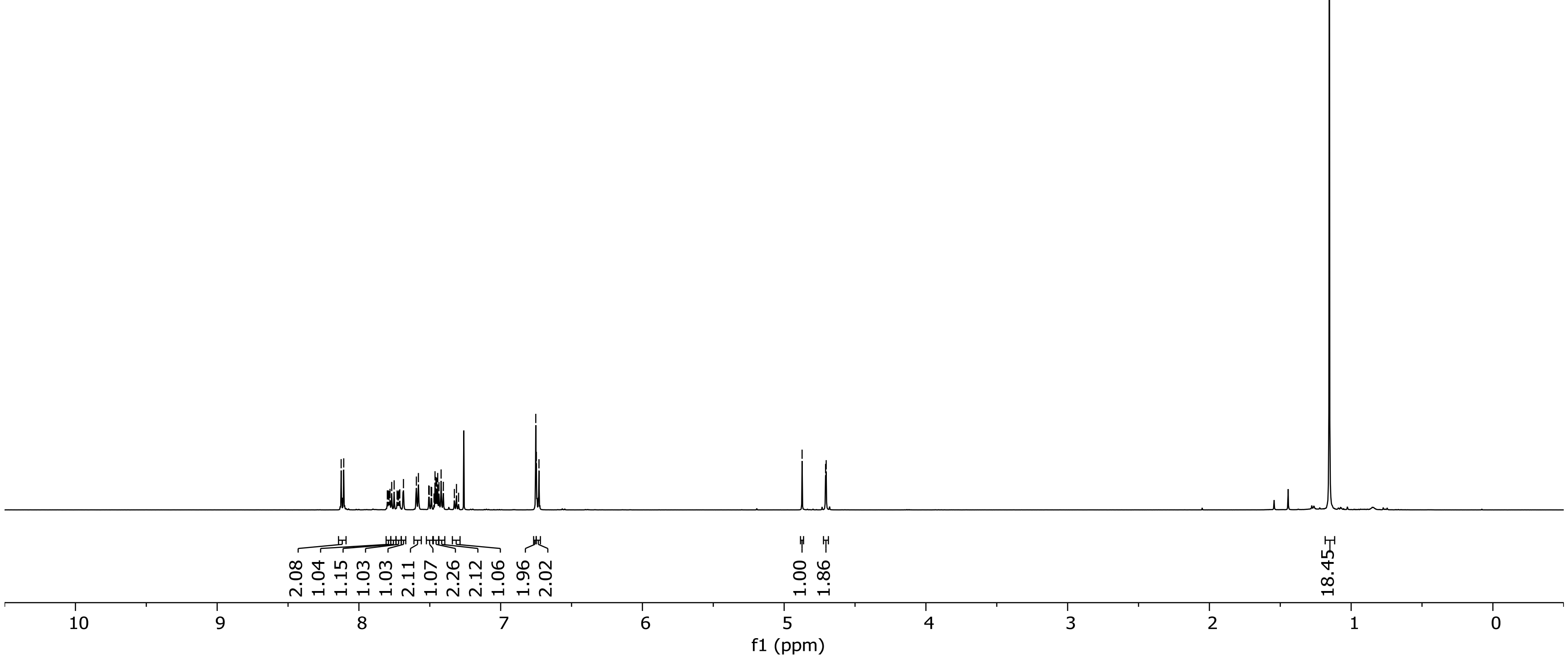

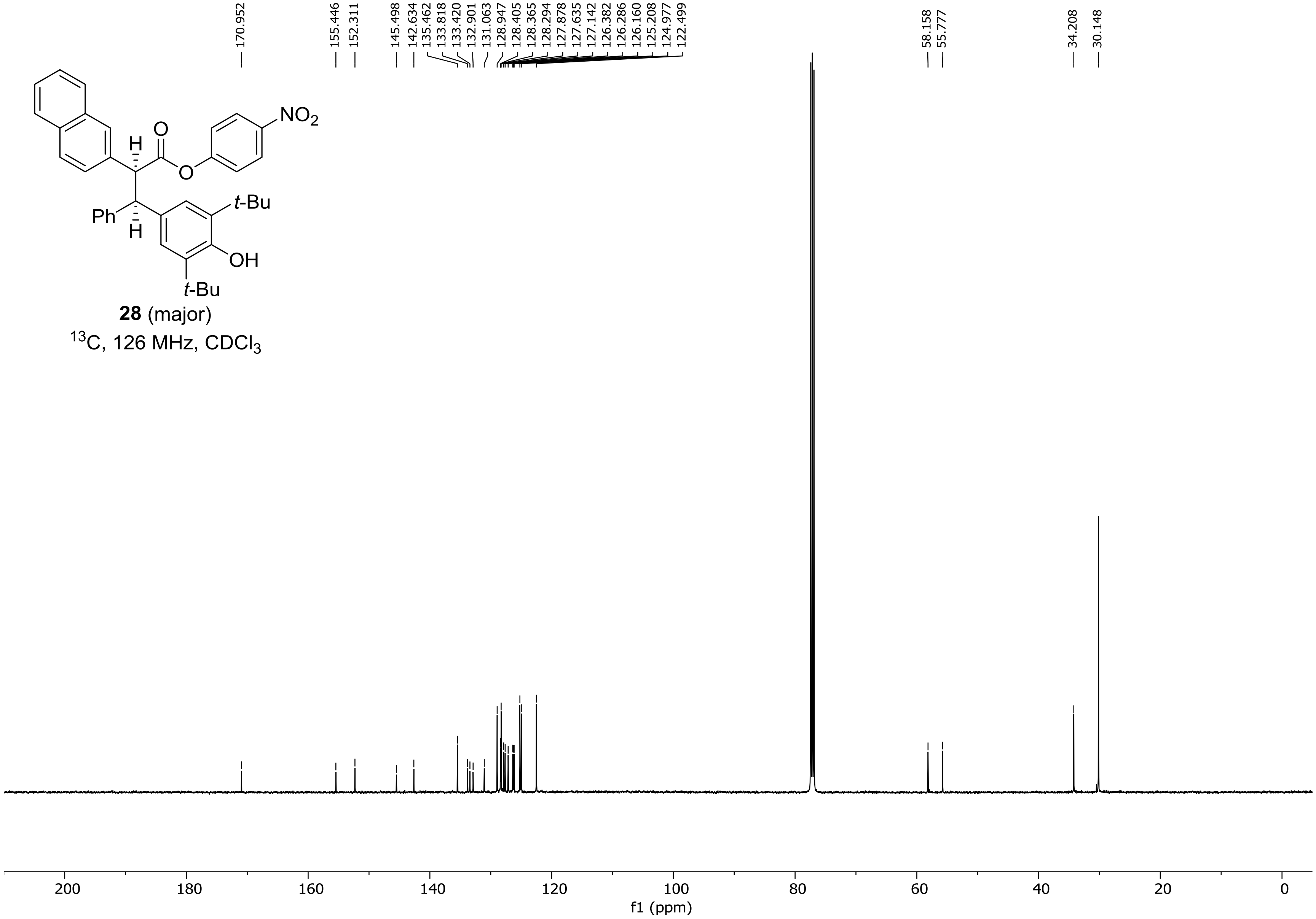

28 (major)

^{13}C, 126 MHz, $CDCl_3$

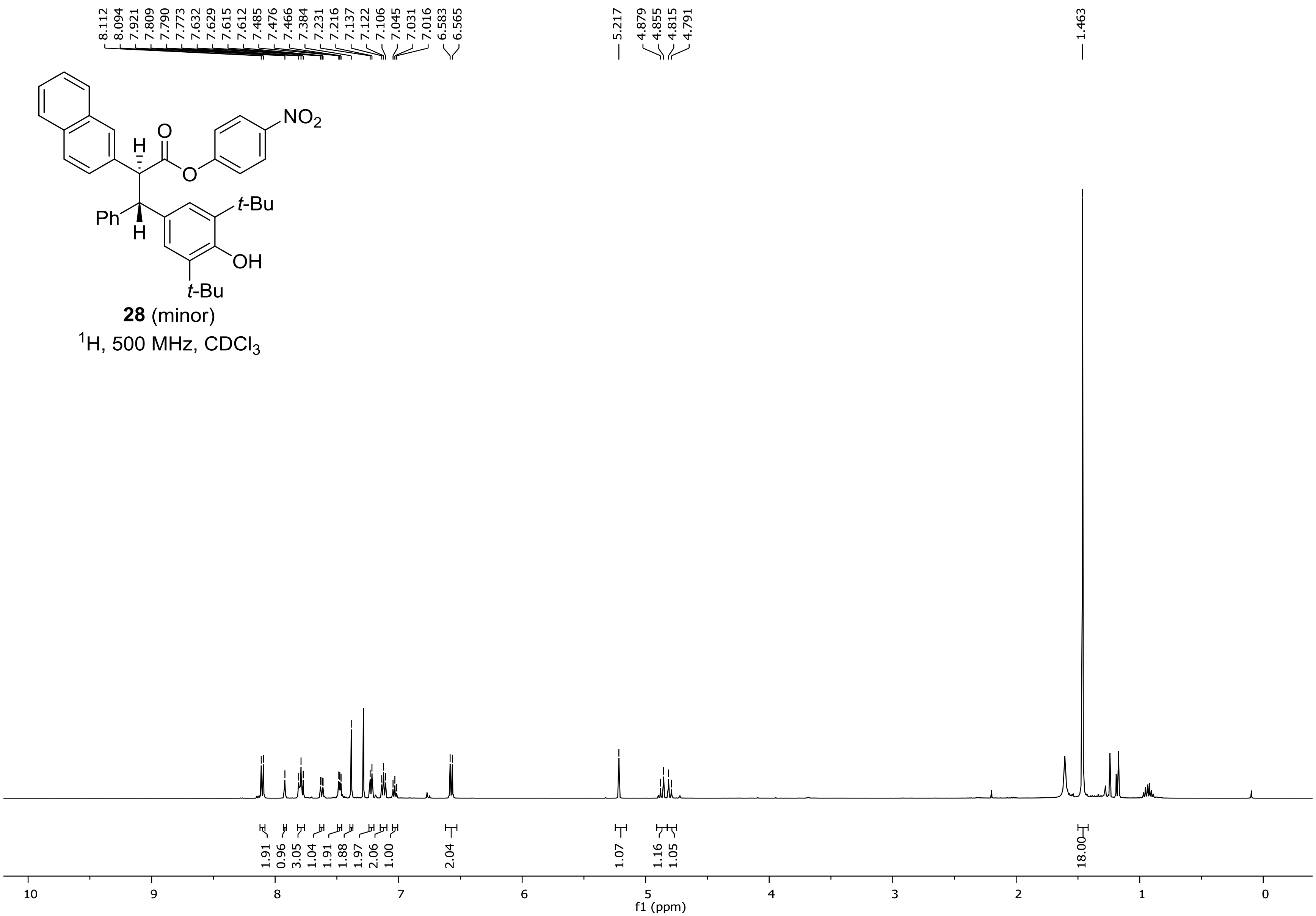
8.112
8.094
7.921
7.809
7.790
7.773
7.632
7.629
7.615
7.612
7.485
7.476
7.466
7.384
7.231
7.216
7.137
7.122
7.106
7.045
7.031
7.016
6.583
6.565
5.217
4.879
4.855
4.815
4.791
1.463
NO2
H
O
O
Ph
H
t-Bu
OH
t-Bu
28 (minor)
1H, 500 MHz, CDCl3
1.91
0.96
3.05
1.04
1.91
1.88
1.97
2.06
1.00
2.04
1.07
1.16
1.05
18.00
10
9
8
7
6
5
4
3
2
1
0
f1 (ppm)

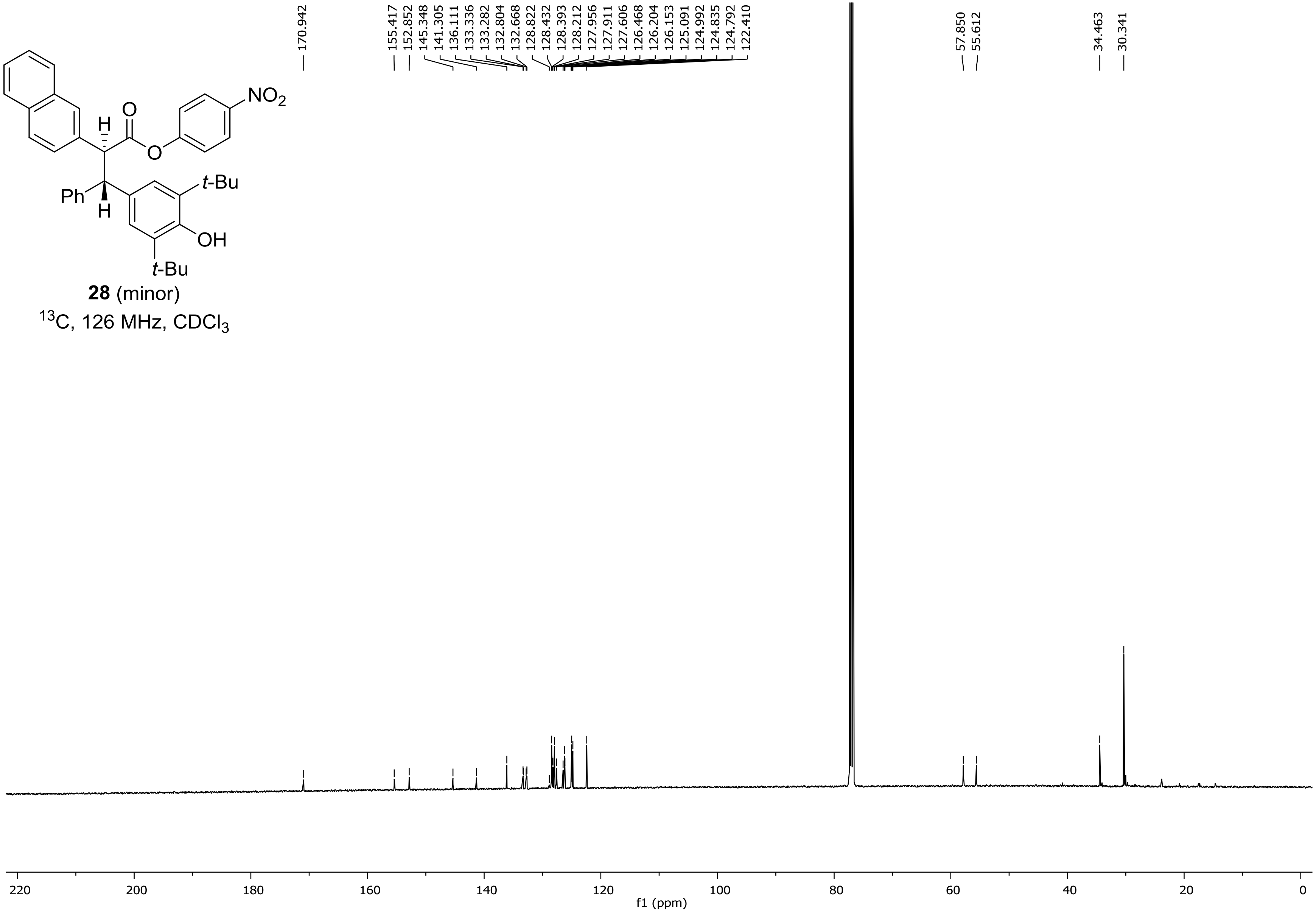
170.942
155.417
152.852
145.348
141.305
136.111
133.336
133.282
132.804
132.668
128.822
128.432
128.393
128.212
127.956
127.911
127.606
126.468
126.204
126.153
125.091
124.992
124.835
124.792
122.410
57.850
55.612
34.463
30.341
NO2
O
H
O
Ph
H
t-Bu
OH
t-Bu
28 (minor)
13C, 126 MHz, CDCl3
220
200
180
160
140
120
100
80
60
40
20
0
f1 (ppm)

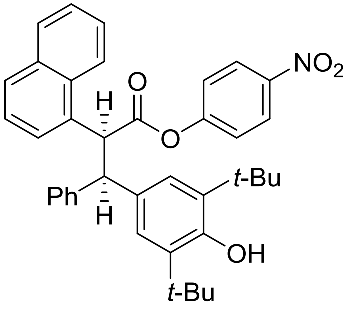

29 (major)

^{1}H, 500 MHz, $CDCl_3$

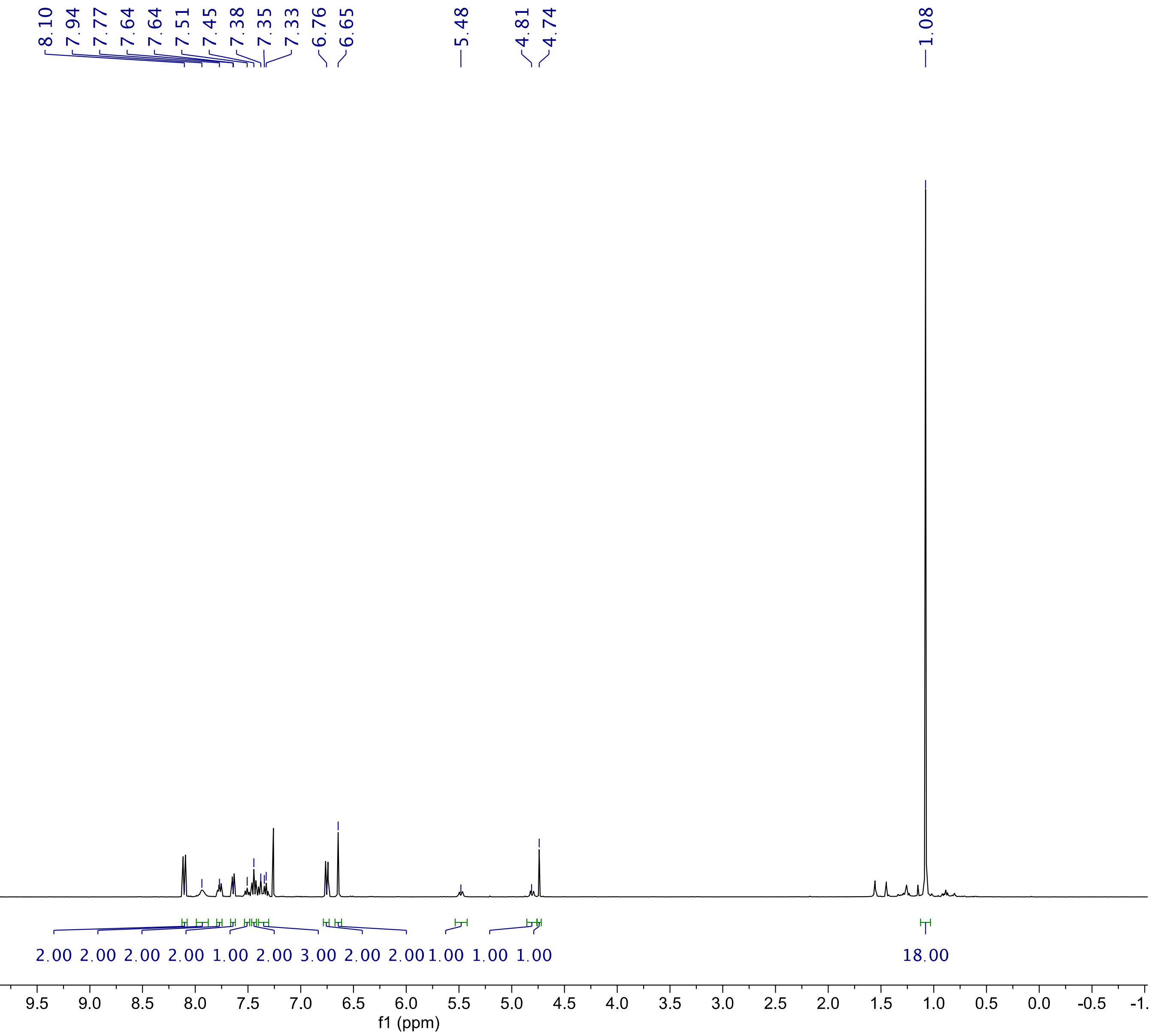

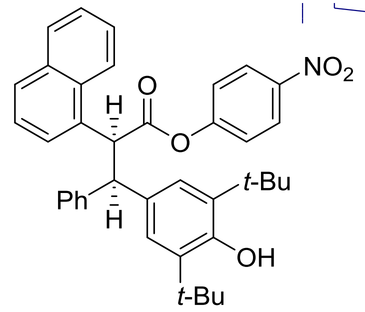

29 (major)

^{13}C, 126 MHz, $CDCl_3$

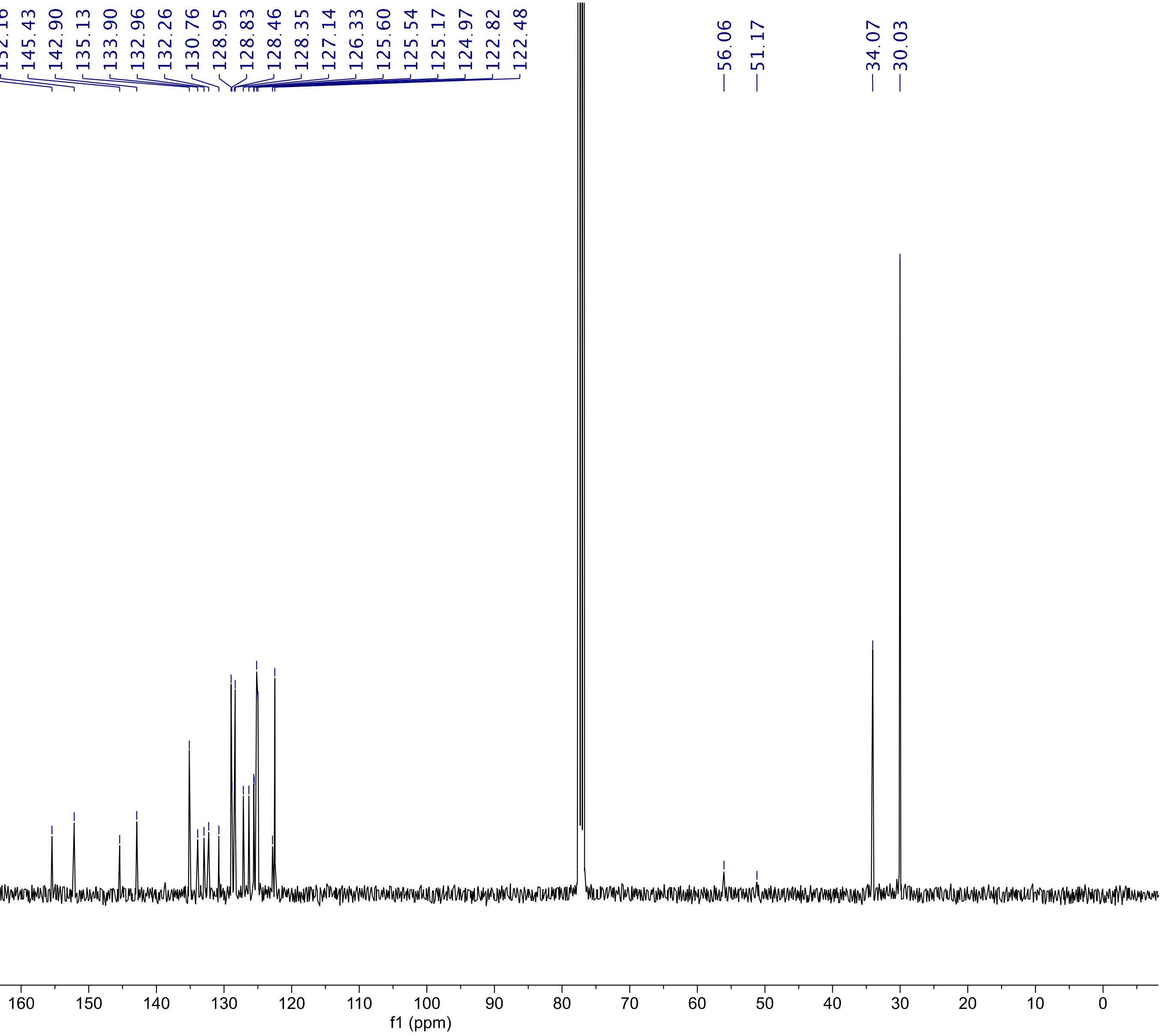

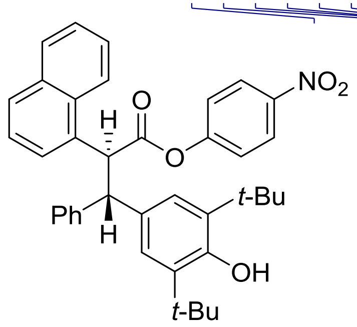

29 (minor)
^{1}H, 500 MHz, $CDCl_3$

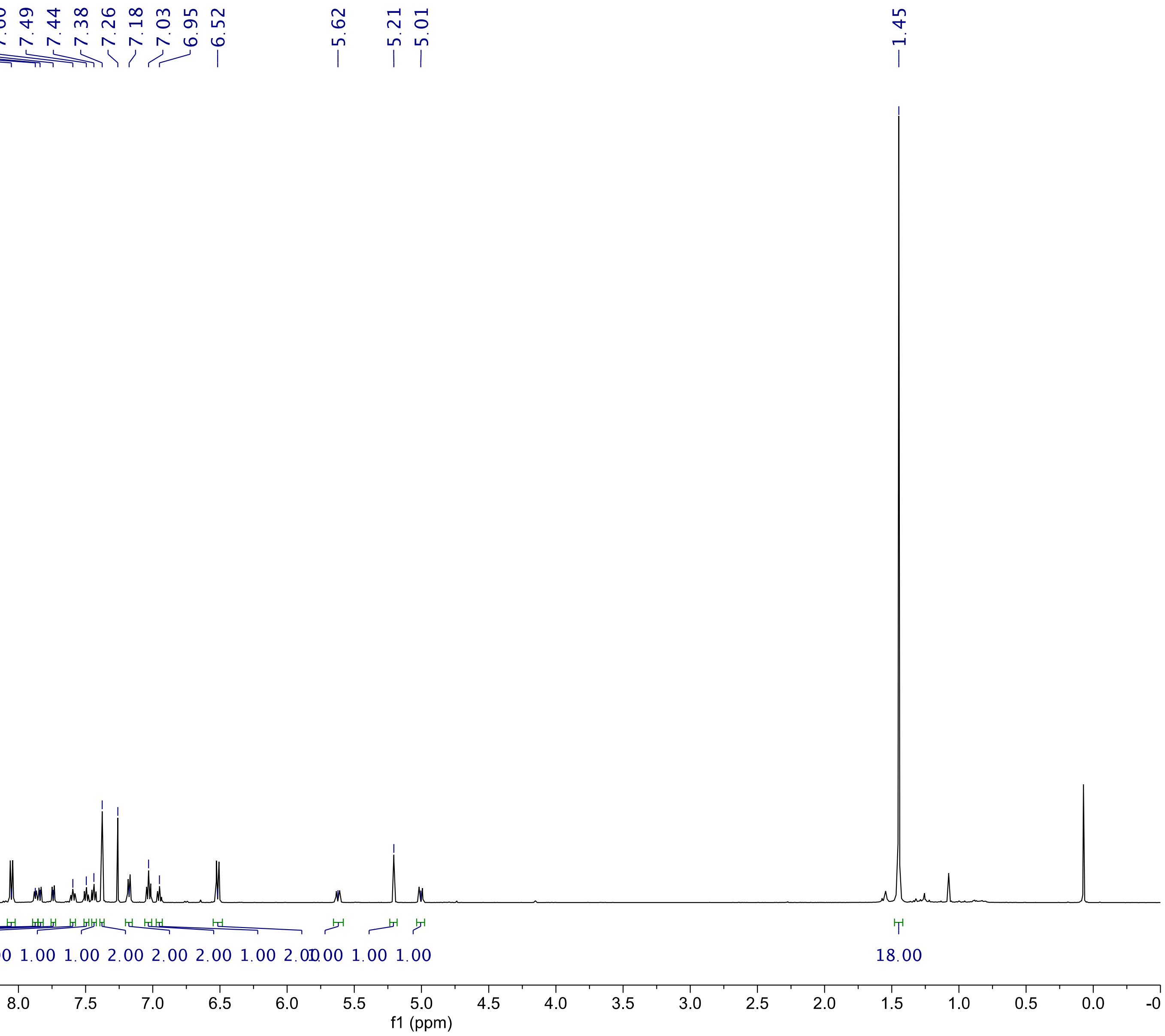

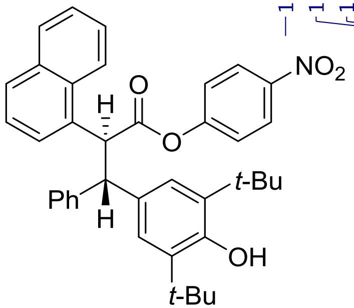

29 (minor)

^{13}C, 126 MHz, $CDCl_3$

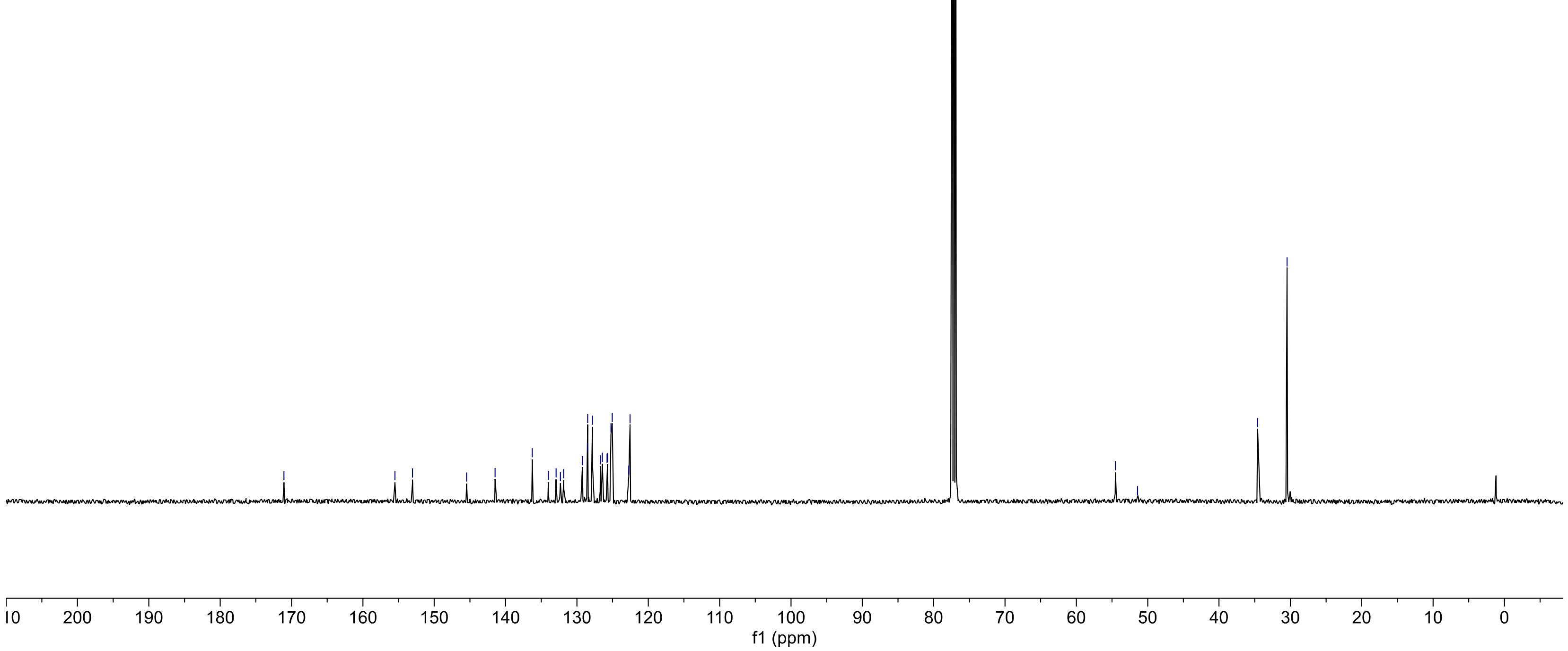

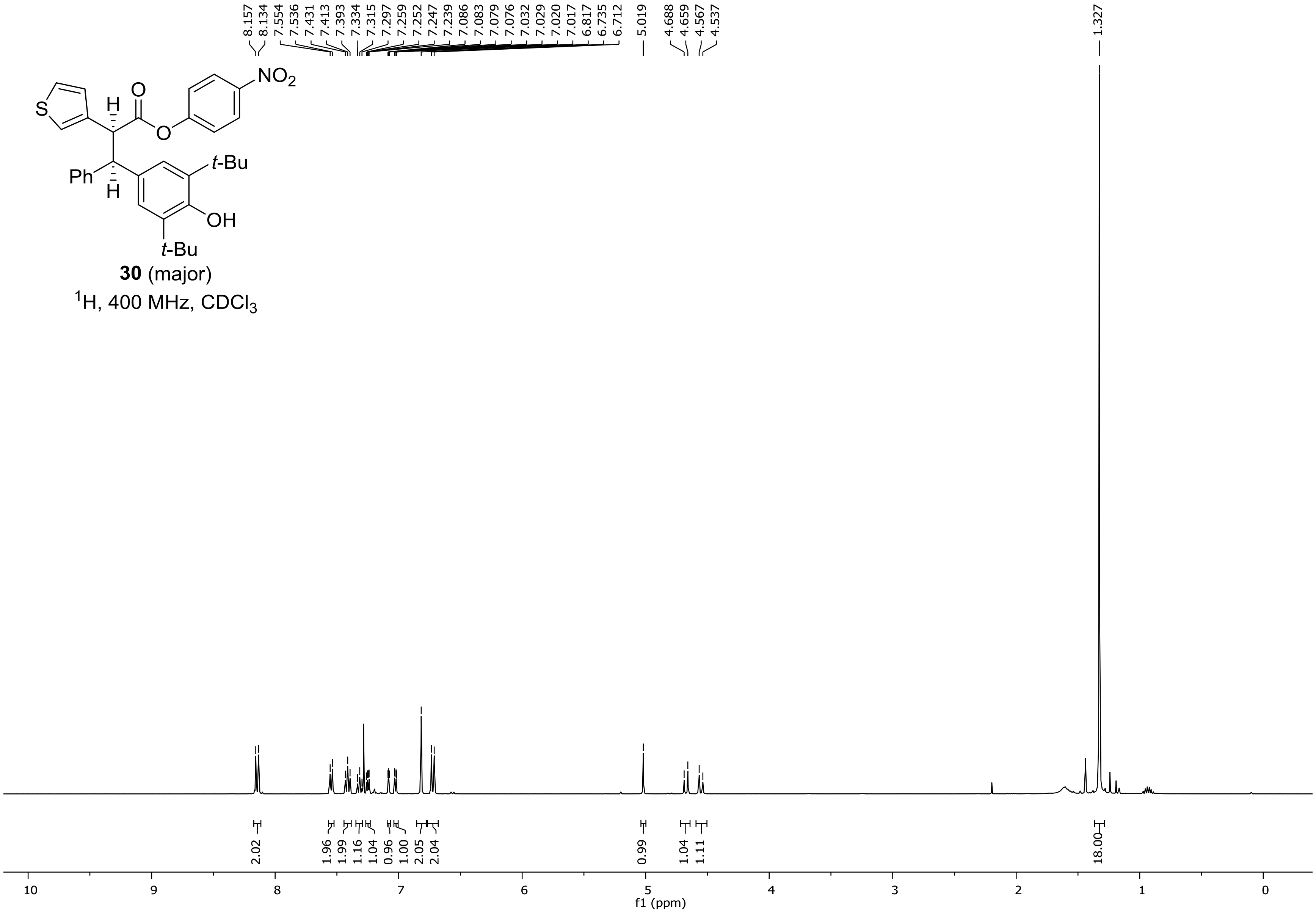
NO2
S
H
O
O
Ph
H
t-Bu
OH
t-Bu
30 (major)
1H, 400 MHz, CDCl3
8.157
8.134
7.554
7.536
7.431
7.413
7.393
7.334
7.315
7.297
7.259
7.252
7.247
7.239
7.086
7.083
7.079
7.076
7.032
7.029
7.020
7.017
6.817
6.735
6.712
5.019
4.688
4.659
4.567
4.537
1.327
2.02
1.96
1.99
1.16
1.04
0.96
1.00
2.05
2.04
0.99
1.04
1.11
18.00
10
9
8
7
6
5
4
3
2
1
0
f1 (ppm)

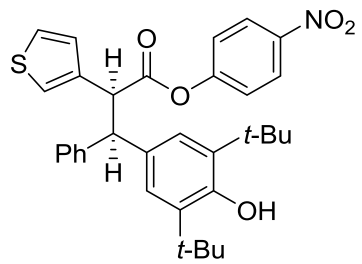

30 (major)

^{13}C, 101 MHz, $CDCl_3$

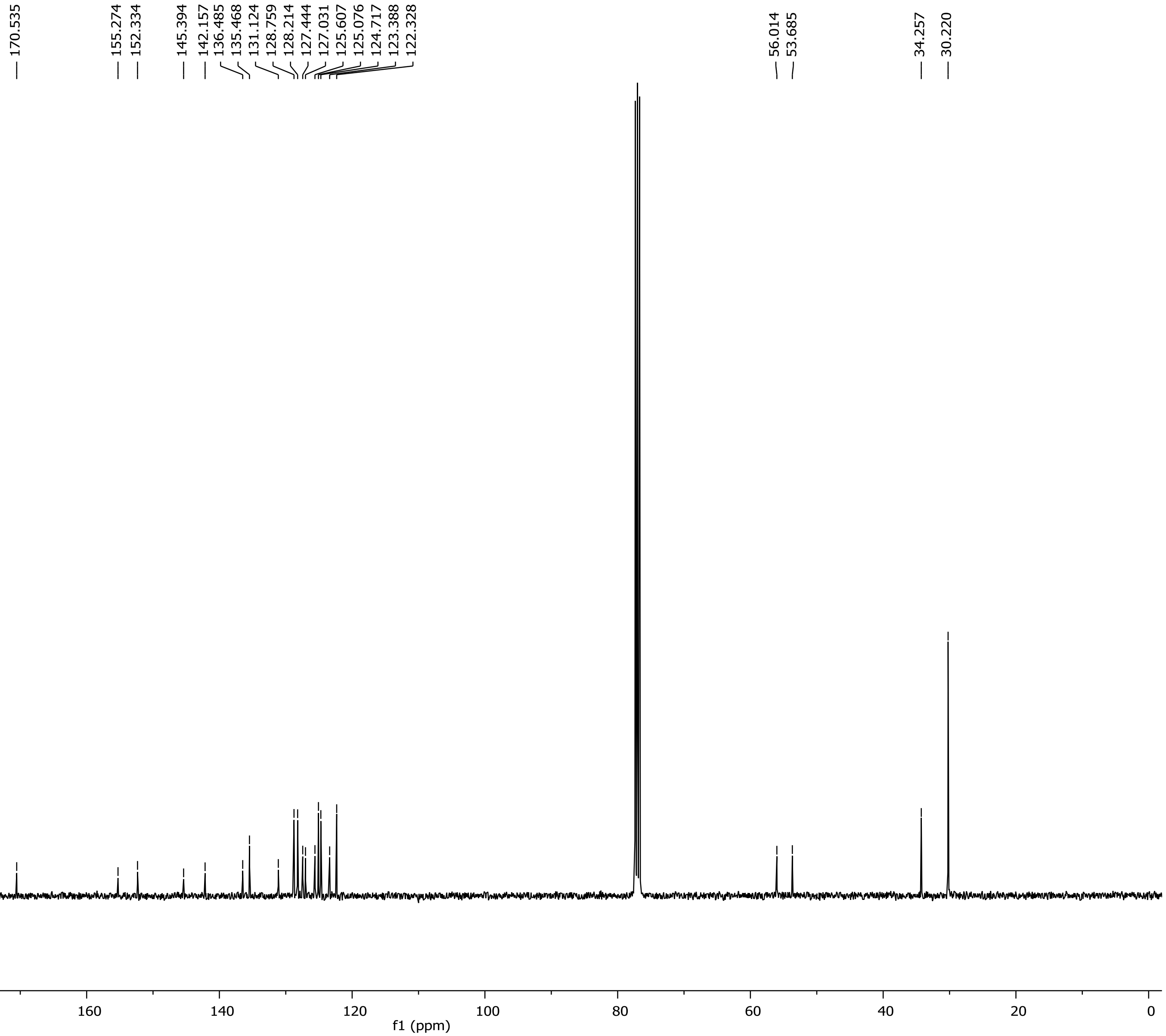

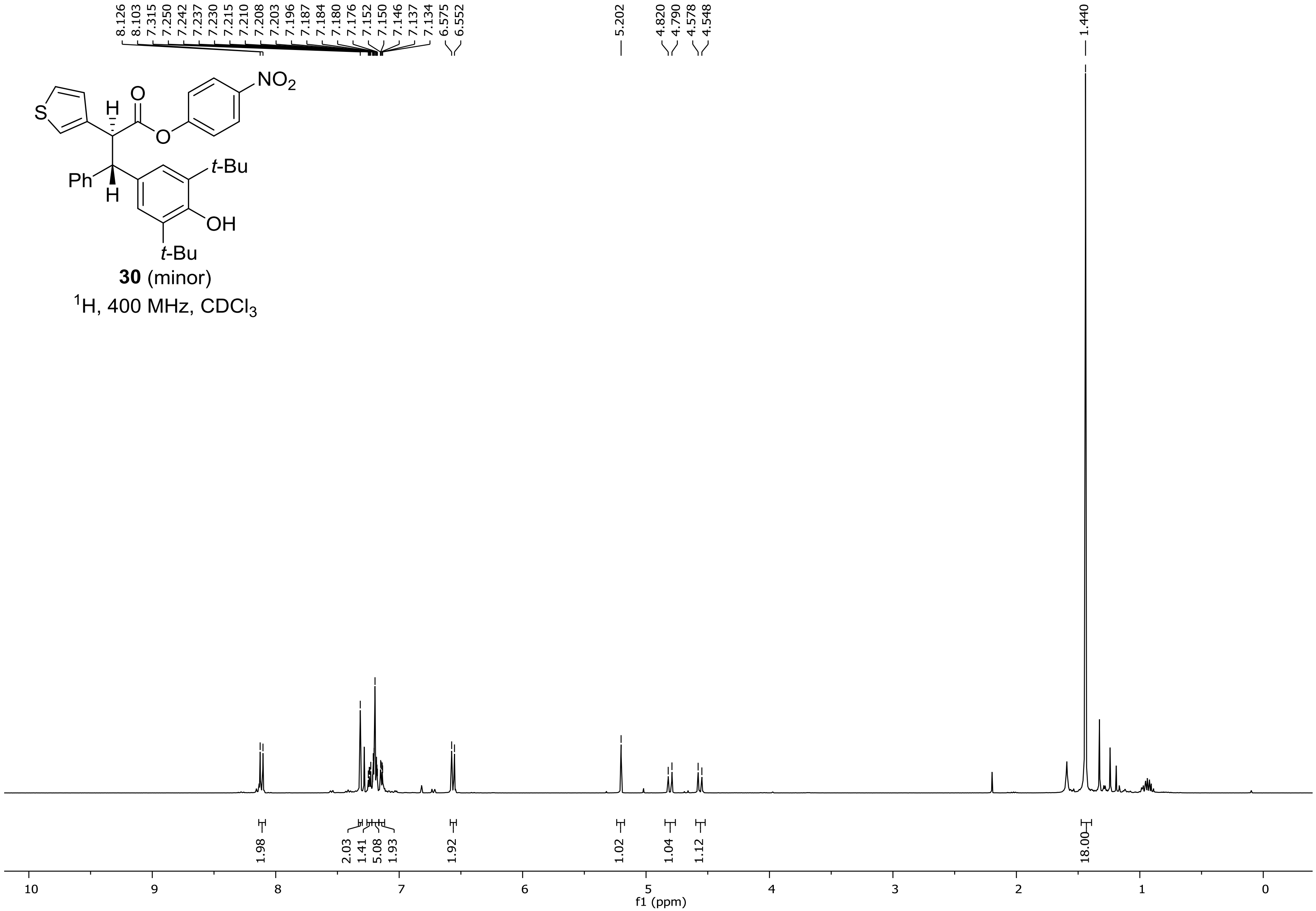
NO2
O
H
S
O
Ph
H
t-Bu
OH
t-Bu
30 (minor)
1H, 400 MHz, CDCl3
8.126 8.103 7.315 7.250 7.242 7.237 7.230 7.215 7.210 7.208 7.203 7.196 7.187 7.184 7.180 7.176 7.152 7.150 7.146 7.137 7.134 6.575 6.552
5.202
4.820 4.790 4.578 4.548
1.440
1.98
2.03 1.41 5.08 1.93
1.92
1.02
1.04
1.12
18.00
10 9 8 7 6 5 4 3 2 1 0
f1 (ppm)

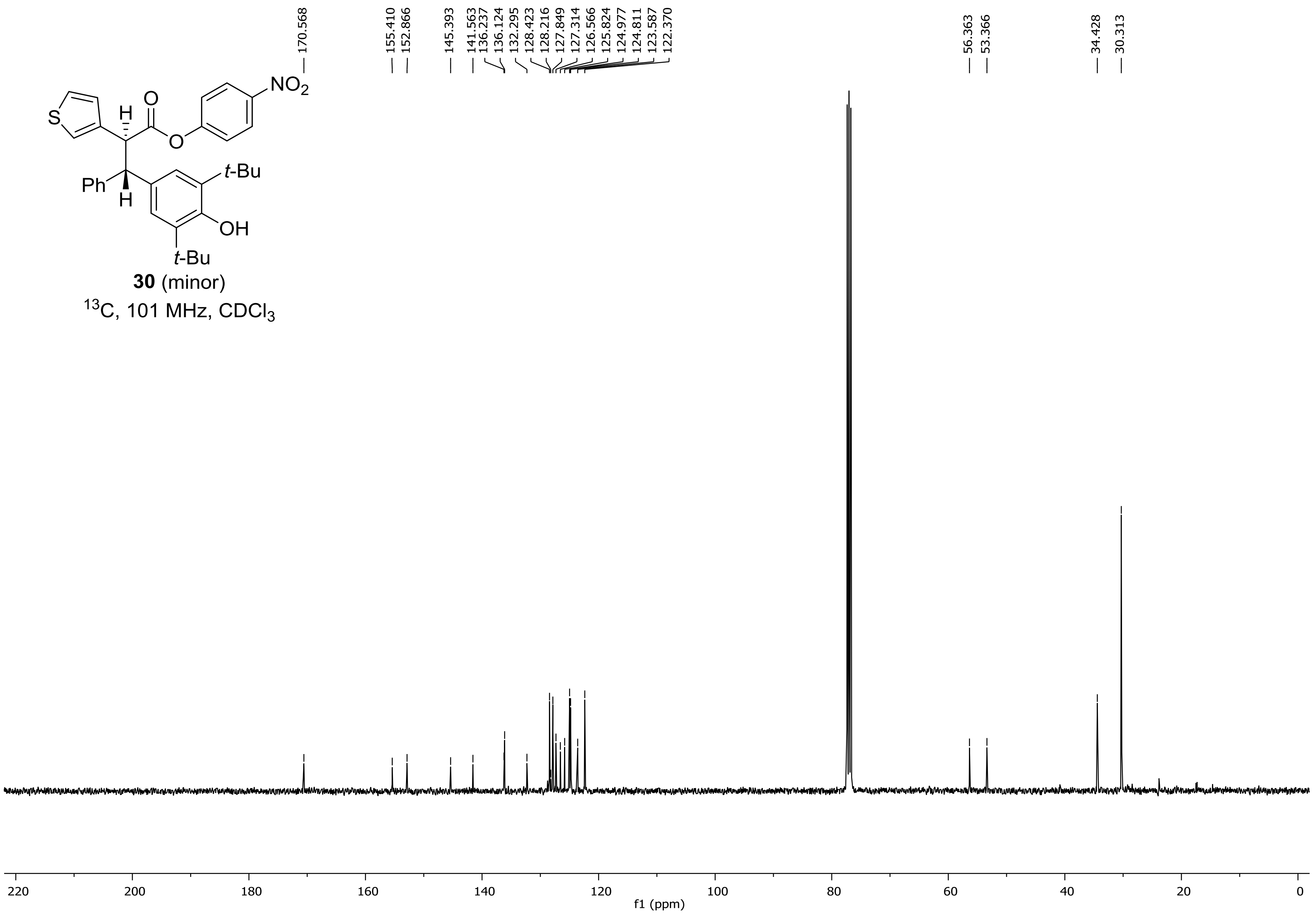

170.568
155.410
152.866
145.393
141.563
136.237
136.124
132.295
128.423
128.216
127.849
127.314
126.566
125.824
124.977
124.811
123.587
122.370
56.363
53.366
34.428
30.313
NO2
O
H
S
Ph
H
t-Bu
OH
t-Bu
30 (minor)
13C, 101 MHz, CDCl3
220
200
180
160
140
120
100
80
60
40
20
0
f1 (ppm)
